# Development and validation of an epidemiological risk score for neonatal death in a middle-income country

**DOI:** 10.3389/fpubh.2025.1675040

**Published:** 2025-11-19

**Authors:** Kelsy N. Areco, Paulo Bandiera-Paiva, Rita C. X. Balda, Daniela T. Costa-Nobre, Ana Sílvia Scavacini Marinonio, Adriana Sanudo, Mandira D. Kawakami, Milton H. Miyoshi, Carina Nunes Vieira e Oliveira, Rosa M. V. Freitas, Monica L. P. Teixeira, Bernadette Waldvogel, Carlos Roberto V. Kiffer, Maria Fernanda de Almeida, Ruth Guinsburg, Tulio Konstantyner

**Affiliations:** 1 Escola Paulista de Medicina, Universidade Federal de São Paulo, São Paulo, Brazil; 2 Fundação Sistema Estadual de Análise de Dados, São Paulo, Brazil

**Keywords:** neonatal mortality, risk score, population studies in public health, secondary health data, death certificates, public health, congenital abnormalities

## Abstract

**Introduction:**

Despite global reductions in neonatal mortality, significant disparities remain between regions and population groups. Identifying newborns at higher risk at birth may help direct preventive actions and enhance health planning.

**Objective:**

To develop an epidemiological risk score for neonatal death based on individual and contextual factors.

**Methods:**

A cohort study was conducted using data from over 5.6 million live births in 645 municipalities of São Paulo State, Brazil, between 2009 and 2018. The outcome was neonatal death. Risk weights were calculated from adjusted odds ratios obtained through multilevel logistic regression, with coefficients transformed using the natural logarithm and scaled from 1 to 10. Internal validation was performed within the cohort; external validation used data from 2008.

**Results:**

Points were assigned to congenital anomalies (4, 7, or 10 depending on severity), preterm vaginal birth (4), preterm cesarean (4), birthweight <2,500 g (4), and fewer than seven prenatal visits (3). Conditions assigned 1 point included male sex, maternal age <17 or ≥40 years, term cesarean, birth in spring/summer, multiple pregnancy, low municipal nurse density in public services, and low municipal health insurance coverage. The area under the ROC curve (AUC) was 0.83 for internal and 0.81 for external validation. Risk stratification thresholds were proposed based on total points.

**Conclusion:**

This score combines routinely collected individual and municipal-level data in Brazil to classify neonatal death risk. It may support clinical prioritization, resource allocation, and identification of low-risk deaths, complementing individualized clinical assessment.

## 1 Introduction

Neonatal mortality (0 to 27 days) has decreased worldwide in recent decades, but large disparities remain between countries and regions. Between 1990 and 2019, the global neonatal mortality rate (NMR) dropped by half, from 36.7 to 17.7 deaths per 1,000 live births (‰ LB). In 2017, Japan had the lowest NMR (0.9‰ LB), while Pakistan had one of the highest (44.2‰ LB), highlighting the gap between high- and low-income countries. Similar inequalities are also observed within middle-income countries like Brazil, where regional differences in access to neonatal care persist (1–4).

In Brazil, neonatal mortality has been the main component of infant mortality since the 1990s. Between 2009 and 2018, the national NMR fell from 11.5 to 9.1‰ LB. Despite this progress, important challenges remain, especially in areas with fewer resources for pregnancy, delivery, and neonatal care, which leads to overcrowded public neonatal intensive care units (NICUs). Studies report difficulties in referring high-risk pregnancies and an uneven distribution of specialized services, which affect the quality of care for newborns. Although policies such as “Stork Network” and the Pact for Reducing Maternal and Neonatal Mortality have improved access to maternal and child healthcare, gaps in prenatal care, delays in receiving assistance, and births in hospitals without such units remain major barriers. Over half of neonatal deaths, mostly occurring in the first week of life, could be prevented through better access to and quality of care during pregnancy, delivery, and the neonatal period (5–15).

These disparities are also clear across Brazil, with significant differences among regions, states, and municipalities. Between 2009 and 2018, the North and Northeast — less developed regions — had the highest NMRs, with states such as Amazonas, Pará, Amapá, and Roraima exceeding 10‰ LB. In contrast, states such as Santa Catarina, Paraná, the Federal District, and São Paulo maintained rates between 7 and 8‰ LB. São Paulo accounted for around 20% of neonatal deaths in Brazil and is also one of the most developed states, with a Municipal Human Development Index (HDI-M), a local version of the global HDI adapted to the Brazilian context, ranging from 0.812 to 0.837 between 2012 and 2018. In São Paulo State, the neonatal mortality rate declined from 11.7 per 1,000 live births in 1990 to 7.4 per 1,000 in 2018, representing a 37% reduction over the period. However, the pace of reduction has slowed in recent years, with rates stabilizing since 2018. In 2022, São Paulo reported an NMR of 7.7‰ LB, while the national average was 8.7‰ LB (6, 16–23).

Neonatal risk scores are tools used to identify newborns at higher risk of morbidity and mortality. These instruments help assess clinical severity, predict adverse outcomes, and guide interventions. They are applied both at birth and during the neonatal follow-up. Most commonly used risk scores rely on clinical and laboratory data and rarely include demographic or contextual variables related to the mother or her environment (24–28).

An epidemiological risk score based on birth data and characteristics of the municipality of residence—including demographic, structural, and healthcare-related aspects—may complement existing tools. This approach aims to identify vulnerable groups at birth, allowing immediate preventive actions and helping to map high-risk areas.

The objective of this study is to develop an epidemiological risk score based on factors associated with neonatal death at different levels in a middle-income country context, including birth characteristics, the presence and severity of congenital malformations and anomalies, and the contextual characteristics of the mothers’ municipalities of residence.

## 2 Methods

### 2.1 Study design and population

This study was designed as a historical, population-based cohort including all live births in the state of São Paulo, Brazil, from 2009 to 2018, whose mothers resided in one of the 645 municipalities of the state. The unit of analysis was the live birth, and the primary outcome was neonatal death (0–27 days of life).

Five complementary analyses were conducted within this cohort to capture different dimensions of risk: (1) a cohort analysis restricted to live births with a single congenital anomaly, used to estimate the relative severity of conditions based on the observed proportion of neonatal deaths; (2) an ecological analysis using municipality- and year-aggregated data to identify contextual health indicators associated with neonatal mortality; (3) a multilevel analysis integrating individual- and municipal-level variables to estimate adjusted risk of neonatal death and construct the Epidemiological Risk Score for Neonatal Death; (4) an internal validation of the score’s discrimination and calibration within the 2009–2018 cohort; and (5) an external temporal validation assessing reproducibility and stability in an independent 2008 cohort.

Each analytical component is described in more detail in the following subsections.

### 2.2 Data source

The data used in this study were obtained from the State System for Data Analysis Foundation (SEADE, acronym in Portuguese) (29) and the Program for the Evaluation of the Health System’s Performance (PROADESS, acronym in Portuguese) (30). Individual-level information included records of live births and infant deaths, extracted from the Live Birth Certificates (LBC) and Death Certificates (DC), stored in annual digital files (.xlsx) provided by SEADE (29). These records covered all live births to mothers residing in the 645 municipalities of São Paulo State between 2009 and 2018, with approximately 600,000 births and 8,000 infant deaths per year (31, 32). Birth information was already linked to infant death records through deterministic linkage performed annually by the institution itself (33, 34). Municipal-level data were obtained from the PROADESS (30) indicator matrix, organized by municipality and year. Indicators were collected individually, with metadata available on the digital platform (35), and municipality codes were standardized according to the official Brazilian Institute of Geography and Statistics (IBGE, acronym in Portuguese) database (36).

### 2.3 Data selection criteria

#### 2.3.1 Individual data

All live births between 2009 and 2018 whose mothers resided in the state of São Paulo were included. Records lacking information on the municipality of residence were excluded. Variables with more than 10% missing values were excluded from the analysis. Incomplete records were disregarded in multivariable analyses.

#### 2.3.2 Municipal data

Municipal indicators were included if they met the following criteria: availability for the period from 2009 to 2018; presented as rates or proportions; relevant to neonatal or maternal health; enabled spatial and temporal comparisons; and did not overlap with information already available in the Live Birth Certificate or include neonatal mortality in their composition. Indicators with more than 10% missing values were excluded.

### 2.4 Data consolidation and integration

#### 2.4.1 Individual data

Annual files of live births and infant deaths were consolidated separately. Neonatal deaths were identified within the infant death records and then matched to the live birth records to determine which newborns had died. This allowed the construction of the variable “neonatal death” (yes/no). Annual totals of consolidated records were presented, including cases with missing information on the mother’s municipality of residence, with the corresponding number of neonatal deaths in those cases.

#### 2.4.2 Municipal data

Municipal indicators from PROADESS (30) were merged, and official municipality codes from IBGE (36) were assigned, since the original records were identified only by name. The final dataset included 6,450 records (645 municipalities × 10 years), structured with one row per municipality per year, and columns representing each included indicator.

### 2.5 Study variables

The variables originally collected are described below, along with those generated during the study for the development of the Neonatal Mortality Risk Score.

#### 2.5.1 Individual data

The outcome was neonatal death (Yes/No), derived from the age-at-death variable.

Original variables included:

- Neonate: birth weight, race/ethnicity, sex, Apgar (1st and 5th minute), congenital malformations coded according to the International Classification of Diseases – 10th Revision (ICD-10) (37)
- Mother: age, education, marital status, previous live/deceased children, prenatal visits
- Pregnancy: gestational age, pregnancy type, delivery type location and date: residence and birth municipality, date of birth

Derived variables:

- Binary: low birth weight, maternal age <17 or ≥40, <7 prenatal visits, multiple pregnancy, delivery outside residence municipality, spring/summer birth
- Combined delivery and gestational age: vaginal/cesarean x < 37/≥37 weeks (38)
- Malformation-related derived variables: A set of variables was derived from individual-level data on congenital malformations and anomalies recorded at birth, including the total number of anomalies and binary indicators for each diagnostic category (Q00–Q99, Chapter XVII of the ICD-10) (37). These variables supported the construction of a severity classification system, described in detail in the next section.

#### 2.5.2 Municipal data

Derived outcome variables:

- Neonatal mortality rate
- Standardized neonatal mortality rate: the variable was standardized (z-score transformation) to have a mean of zero and a standard deviation of one

##### 2.5.2.1 Planned and derived independent municipal variables

The municipal variables correspond to performance indicators of the Brazilian health system, available from the referenced source, and were included based on predefined selection criteria (35, 39).

Derived independent municipal variables:

*Standardized variables*: All selected municipal indicators were converted into z-scores, with a mean of zero and a standard deviation of one.
*Dichotomous variables*: For each indicator included in the multiple regression analysis, a binary version was created using the mean as the cutoff (< mean / ≥ mean).

#### 2.5.3 Final variable: neonatal mortality risk score

Ordinal risk score combining individual and municipal predictors, based on adjusted coefficients from the final multivariable analysis to classify live births according to their risk of neonatal death.

### 2.6 Procedures prior to score modeling

The initial selection of independent variables was based on univariate analysis, using *p* < 0.20 for screening. Variables meeting this criterion were tested in multivariable models, with multicollinearity assessed using the Variance Inflation Factor (VIF), considering acceptable values between 1 and 2 (40).

The results of descriptive and inferential analyses were presented in text, tables, or figures, and included as Supplementary material when necessary.

#### 2.6.1 Descriptive analysis and completeness of individual and municipal data

After applying exclusion criteria, individual data were unified and checked for consistency (41), with completeness assessed by the frequency of missing values; individual and municipal variables were described in both their original and derived forms using appropriate descriptive statistics, and the outcome (neonatal death) was analyzed by year of birth.

#### 2.6.2 Selection of independent individual variables

Derived variables related to the newborn, mother, pregnancy, delivery, and geographic and temporal context were created to support the selection of independent variables for the model. Each variable was individually analyzed using univariate logistic regression, with odds ratios and 95% confidence intervals estimated. Variables with *p* < 0.20 were selected for multilevel modeling, which was used to construct the risk score. The absence of multicollinearity between the selected derived variables and the severity classification of malformations was confirmed by the Variance Inflation Factor (VIF) (40, 42).

#### 2.6.3 Severity classification of congenital malformations and anomalies

The severity classification of congenital malformations and anomalies was based on diagnoses recorded at birth. An ordinal variable was created for inclusion in the risk score. The classification followed these steps:

##### 2.6.3.1 Separation and validation of recorded codes

Congenital malformations and anomalies were recorded in a single text field containing comma-separated ICD-10 codes (categories or subcategories). For analysis, only the first three characters of each code were considered, grouping subcategories under the same category (e.g., Q00.0 was grouped as Q00, which includes all diagnoses related to anencephaly and similar malformations). Codes were split and validated according to Chapter XVII of the ICD-10, with only one occurrence per category retained in cases of duplication (37).

##### 2.6.3.2 Creation of derived variables

The total number of congenital malformations and anomalies recorded at birth was calculated for each individual. In addition, 100 binary variables were created to indicate the presence (1) or absence (0) of each diagnostic category from Q00 to Q99, based on Chapter XVII of the ICD-10 (37).

##### 2.6.3.3 Selection of unique cases

A subset of live births with only one malformation or anomaly category recorded was selected, based on the count of distinct ICD-10 categories assigned to each individual (37).

##### 2.6.3.4 Calculation of frequencies and proportions

For each category present in the subset of unique cases, the absolute frequency and the proportion of neonatal deaths were calculated.

##### 2.6.3.5 Assignment of severity grades

Each category was classified into a severity grade according to the following criteria:

- Grade 1: Fewer than 10 cases or neonatal death proportion < 20%
- Grade 2: Neonatal death proportion between 20 and 40%
- Grade 3: Neonatal death proportion ≥ 40%

This empirical classification was subsequently reviewed to ensure clinical coherence and biological plausibility.

##### 2.6.3.6 Assignment of severity to unique categories

The variable *Severity of unique malformation or anomaly category* was assigned exclusively to live births with a single recorded category, according to the severity criteria (Grades 1 to 3).

##### 2.6.3.7 Association analysis with the outcome

The association between the severity of congenital malformations and anomalies and the risk of neonatal death was analyzed using univariate logistic regression. Odds ratios (OR) and 95% confidence intervals were estimated. This analysis was performed only in the subset of live births with a single malformation or anomaly category recorded.

For application in the risk score, the variable *Severity of congenital malformations and anomalies* was assigned to all live births based on the most severe category recorded. Live births without any malformation or anomaly were assigned a value of 0, and those with a malformation or anomaly present but without a specified category were assigned Grade 1.

#### 2.6.4 Selection of independent municipal indicators

##### 2.6.4.1 Construction of the municipal outcome variable

To identify municipal indicators associated with neonatal mortality, an aggregated outcome variable was created. Based on individual data, the total number of live births and neonatal deaths was calculated for each year and municipality of maternal residence. From these totals, an estimated Neonatal Mortality Rate (NMR) was computed by year and municipality.

This estimate considered neonatal deaths among live births in the same calendar year but did not capture cross-year deaths (i.e., newborns from the previous year who died in the current year were not included, while those born at the end of the year and who died in the following year were). Despite this limitation, the estimate was used as a proxy for the rate, assuming relative stability in annual birth and mortality patterns. The municipal neonatal mortality rate variable was merged with the corresponding indicators by year and municipality.

##### 2.6.4.2 Analysis and selection of indicators

Municipal indicators were first described using measures of central tendency and variability. To identify independent indicators associated with NMR, all variables were standardized, and Pearson correlation was used to screen those with *p* < 0.20 for further analysis. Correlated indicators (r ≥ 0.50) were grouped, and only one indicator per group was retained to reduce multicollinearity.

Multiple linear regression models were performed using a stepwise procedure, retaining variables with *p* < 0.05 and Variance Inflation Factor (VIF) between 1 and 2. The model with the highest adjusted R^2^ was selected as the final model (40).

##### 2.6.4.3 Integration of municipal indicators into individual-level data

After selection, the municipal indicators were merged into the individual-level dataset by matching the year of birth and the mother’s municipality of residence, allowing contextual data to be incorporated into multilevel models.

### 2.7 Generation of the neonatal mortality risk score

The score was developed using individual-level data from live births and municipal indicators. It resulted in an ordinal variable representing increasing levels of neonatal mortality risk, also analyzed in dichotomous format, with different cutoff points for application.

### 2.8 Statistical modeling

The statistical modeling of the neonatal mortality risk score was based on a retrospective cohort of approximately 6 million live births in the state of São Paulo (2009–2018), with individual data linked to previously selected municipal indicators from PROADESS (30). Variables were organized into two levels (individual and municipal).

Given the hierarchical structure of the data, multilevel logistic regression was used to estimate odds ratios (OR) associated with neonatal death. The model was fitted using the ENTER method, including individual variables with *p* < 0.20 and municipal indicators with *p* < 0.05, retaining only those with statistical significance (*p* < 0.05) and VIF between 1 and 2. Adjusted ORs with 95% confidence intervals were estimated (40, 42).

### 2.9 Score calculation

The approach adopted for constructing the Epidemiological Neonatal Mortality Risk Score was based on methodologies previously applied in epidemiological studies assessing aggregated risk in infant populations, which use adjusted odds ratios (OR) from logistic regression models to estimate individual and combined risks (43). In this study, a multilevel logistic regression model was applied to a retrospective, population-based cohort of approximately 6 million live births in São Paulo State (2009–2018), linking individual-level birth and neonatal death records to municipal health indicators. The model incorporated two analytical levels — individual (level 1) and municipal (level 2) — to account for the hierarchical data structure.

Adjusted ORs with 95% confidence intervals were estimated for all predictors retained in the final model, using the ENTER method, which included individual variables with *p* < 0.20 and municipal indicators with *p* < 0.05, and excluded those that were not statistically significant (*p* ≥ 0.05). Multicollinearity was assessed through variance inflation factors (VIF), maintaining variables within acceptable limits (VIF between 1 and 2). The ORs derived from the multilevel model were transformed into scaled weights ranging from 1 to 10 by applying a linear transformation to their natural logarithms (lnOR). The transformation followed the equation:


Weight=a×ln(OR)+b

Where a and b are scaling constants defined to map the minimum ln(OR) to weight 1 and the maximum ln(OR) to weight 10, using:


$$\left\{ \begin{array}{c} a=(10–1)/[\text{maximum}\;\ln(\text{OR})–\text{minimum}\;\ln(\text{OR})]\\ b=1–a\times \text{minimum}\;\ln(\text{OR}) \end{array}\right.$$

This transformation was applied to each variable category, and the resulting values were rounded to the nearest integer. A value of zero was assigned to the reference categories, representing the baseline risk.

The final score for each live birth corresponded to the sum of all category scores, reflecting the cumulative contribution of each factor to the neonatal mortality risk. The resulting variable was treated as ordinal, representing increasing levels of neonatal death risk, and was also analyzed in a dichotomous format with defined cutoffs for practical application.

### 2.10 Score validation

The score was validated in two stages: internal validation, using the development cohort (2009–2018), and temporal external validation, using an independent dataset of live births from 2008 in the state of São Paulo, from the same target population. In both stages, score distribution, discriminatory ability, and calibration were evaluated.

#### 2.10.1 Internal validation

Internal validation was performed by applying the score to the development cohort. The ordinal score distribution was described using absolute and relative frequencies.

Discrimination was assessed using the receiver operating characteristic (ROC) curve, area under the curve (AUC), sensitivity, specificity, positive predictive value (PPV), and negative predictive value (NPV). The optimal cutoff point was identified using the Youden index (Sensitivity + Specificity – 100) (44–46).

The association between score and neonatal death was explored using three plots: deaths by score, PPV by score, and ln(OR) by score, estimated via simple logistic regression, which also provided the pseudo *R*^2^ (47).

Four score ranges were defined to reflect increasing risk levels. The classification, based on score distribution and the observed pattern of deaths, aimed to create distinct groups with consistent risk, adequate sample size, and increasing PPVs, supporting practical score application.

Calibration was assessed by comparing observed death rates with the mean score per group. A grouped calibration curve was plotted (mean score on X-axis, observed death rate on Y-axis) to illustrate agreement between predicted and observed values.

#### 2.10.2 External validation

External validation was performed in an independent 2008 cohort of live births from São Paulo state, covering the same 645 municipalities. The score was applied using the same risk categories and cutoff points defined previously.

Distribution, discrimination (ROC curve, AUC, sensitivity, specificity, PPV, NPV), and calibration were evaluated using the same metrics as in internal validation. Calibration was based on the comparison between observed neonatal death rates and the mean score per risk group.

Score distribution and neonatal death proportions were also compared between cohorts to assess consistency across datasets.

### 2.11 Computational resources

The following software was used in the development of the study: Software: Microsoft® Office Professional 2010® (48), Microsoft® SQL Server 2012 Express® (49), Stat/Transfer v14® (50), and StataSE v17 (64-bit)® (51).

### 2.12 Ethical considerations

The study was approved on November 9, 2021, by the Research Ethics Committee of the Federal University of São Paulo, under opinion number 0838/2021 (CAAE: 49897521.1.0000.5505), with informed consent waived.

## 3 Results

### 3.1 Study population

Between 2009 and 2018, a total of 6,114,624 live births and 68,128 infant deaths were recorded, of which 47,185 (70%) occurred in the neonatal period. Among these, 47,044 neonatal deaths (99.7%) were successfully linked to the corresponding live birth records of infants born to mothers residing in the state of São Paulo (Supplementary material 1). During the same period, 1,446 live births (0.02%) lacked information on the mothers’ municipality of residence, including eight neonatal deaths. After excluding cases with missing information on the mother’s municipality, the study cohort comprised 6,113,178 live births and 47,036 neonatal deaths (Supplementary material 2). This cohort was used for the descriptive and exploratory analyses presented in the study.

The following variables presented more than 10% missing values and were excluded from the analysis: Child’s Race/Color, Mother’s Schooling, Apgar Score at 1 and 5 min, Mother’s Marital Status, and Number of Living and Deceased Children from previous pregnancies. Among the variables retained, the following proportions of missing values were observed: Birthweight (0.9%), Maternal Age (0.02%), Gestational Age (3.6%), Prenatal Visits (3.7%), Type of Delivery (0.4%), and Presence of Congenital Malformations and/or Chromosomal Anomalies (2.7%). No missing values were found for Child’s Sex, Type of Pregnancy, Date of Birth, or Municipality of Birth (Supplementary material 3).

### 3.2 Outcome variable

The neonatal mortality rate decreased from 8.66 to 7.33 per 1,000 live births between 2009 and 2018. For the full period, the overall neonatal mortality rate was 7.69 per 1,000 live births (data not shown). Annual rates and the number of deaths are presented in Supplementary material 4.

### 3.3 Characteristics of live births

Table 1 presents the distribution of live birth characteristics, with variables dichotomized or categorized for analysis. Most newborns were female (51.2%) and had a birthweight ≥2,500 g (90.8%). The majority of mothers were aged 17 to 39 years (93.4%), had attended seven or more prenatal visits (78.0%), and experienced a singleton pregnancy (97.6%). Cesarean deliveries accounted for 59.2% of births. Prematurity (<37 weeks) was observed in 10.5% of cases, and 23.5% of deliveries occurred in a municipality different from the mother’s place of residence. Most births took place during autumn and winter (52.2%). The presence of congenital malformations or chromosomal anomalies was reported in 1.0% of live births. The complete distribution of variables in their original format is provided in Supplementary material 5.

**Table 1:** Distribution of live births’ characteristics, State of São Paulo, 2009–2018.

Variable	Category	*N*	%
Sex	Female	2,983,146	51.2
	Male	3,129,933	48.8
	Total	6,113,079	100.0
Birth weight	≥2,500 g	5,498,292	90.8
	<2,500 g	557,786	9.2
	Total	6,056,078	100.0
Maternal age	17–39 years	5,706,587	93.4
	<17 or ≥40 years	405,495	6.7
	Total	6,112,082	100.0
Delivery type	Vaginal	2,481,066	40.8
	Cesarean	3,606,273	59.2
	Total	6,087,339	100.0
Prenatal visits	<7	1,296,751	22.0
	≥7	4,591,290	78.0
	Total	5,888,041	100.0
Multiple pregnancy	No	5,965,570	97.6
	Yes	146,082	2.4
	Total	6,111,652	100.0
Gestational age <37 weeks	No	5,274,160	89.5
	Yes	615,691	10.5
	Total	5,889,851	100.0
Moved for delivery	No	4,675,418	76.5
	Yes	1,437,740	23.5
	Total	6,113,158	100.0
Delivery type and gestational age	Vaginal delivery, ≥37 weeks	2,154,913	36.6
	Cesarean delivery, ≥37 weeks	3,114,980	52.9
	Cesarean delivery, <37 weeks	380,517	6.5
	Vaginal delivery, <37 weeks	234,542	4.0
	Total	5,884,952	100.0
Season of birth	Autumn/Winter	3,194,132	52.2
	Spring/Summer	2,918,838	47.8
	Total	6,112,970	100.0
Congenital malformation or chromosomal anomaly	Yes	60,120	1.0
	No	5,787,636	99.0
	Total	5,847,756	100.0

### 3.4 Classification of congenital malformations and anomalies

Among the 6,113,178 live births in the development cohort, 5,847,756 records (95.7%) contained valid information regarding the presence or absence of congenital malformations and/or chromosomal anomalies. Among these, 60,120 cases (1.02%) indicated the presence of at least one anomaly, with 8.7% of them lacking specification of the anomaly type (Table 2; Supplementary material 5).

**Table 2:** Univariate logistic regression: individual variables and association with neonatal death, State of São Paulo, 2009–2018.

Variable	Odds ratio	95% CI (Lower)	95% CI (Upper)	*N*	Pseudo *R*^2^
Male sex	1.21	1.19	1.23	6,113,079	0.0008
Birth weight <2,500 g	34.76	34.01	35.53	6,056,078	0.2270
Maternal age <17 or ≥40 years	1.62	1.57	1.67	6,112,082	0.0016
<7 prenatal visits	6.22	6.10	6.34	5,888,041	0.0671
Multiple pregnancy	5.48	5.33	5.64	6,111,652	0.0163
Moved for delivery	1.30	1.27	1.32	6,113,158	0.0011
Birth in Spring/Summer	1.08	1.06	1.09	6,112,970	0.0001
Cesarean delivery and gestational age ≥37 weeks	1.18	1.13	1.22	5,884,952	0.2056
Cesarean delivery and gestational age <37 weeks	24.19	23.38	25.04		
Vaginal delivery and gestational age <37 weeks	40.67	39.29	42.09		
Congenital anomalies classification				5,847,756	0.0756
Severity grade 1	11.88	11.46	12.32		
Severity grade 2	73.61	70.30	77.07		
Severity grade 3	374.76	341.45	411.32		

Of the 54,883 specified anomalies, 84% were classified as isolated and 16% as multiple (Supplementary material 6). Among the isolated cases, 84% (46,102) were classified as severity grade 1, 13.1% as grade 2, and 2.9% as grade 3. The detailed distribution of severity classification is presented in Supplementary material 7.

### 3.5 Variables associated with neonatal death

Univariate logistic regression showed statistically significant associations (*p* < 0.001) between neonatal death and the following characteristics: male sex (OR = 1.21; 95%CI: 1.19–1.23), birthweight <2,500 g (OR = 34.76; 95%CI: 34.01–35.53), maternal age <17 or ≥40 years (OR = 1.62; 95%CI: 1.57–1.67), fewer than seven prenatal visits (OR = 6.22; 95%CI: 6.10–6.34), multiple pregnancy (OR = 5.48; 95%CI: 5.33–5.64), and intermunicipal migration for delivery (OR = 1.30; 95%CI: 1.27–1.32). Births occurring in spring/summer were also associated with higher risk of neonatal death (OR = 1.08; 95%CI: 1.06–1.09). In the combined analysis of delivery type and gestational age, the highest risks were observed for preterm vaginal deliveries (OR = 40.67; 95%CI: 39.29–42.09), using term vaginal deliveries (≥37 weeks) as the reference category (Table 2).

The analysis also included the classification of congenital malformations and anomalies according to severity. A progressive increase in neonatal death risk was observed across severity levels: OR = 11.88 (95%CI: 11.46–12.32) for grade 1, OR = 73.61 (95%CI: 70.30–77.07) for grade 2, and OR = 374.76 (95%CI: 341.45–411.32) for grade 3. Pseudo *R*^2^ values ranged from 0.0001 (birth during spring/summer) to 0.2270 (birthweight <2,500 g), indicating varying explanatory power of the predictors. All variables were statistically associated with the outcome (*p* < 0.001; Table 2). Variance Inflation Factor values ranged from 1.00 to 1.28, with a mean of 1.07 (data not shown).

### 3.6 Municipal data

A total of 24 municipal indicators were obtained from the PROADESS-Fiocruz platform, resulting in 6,450 records (FRONT31). Most indicators had complete data, except for the percentage of municipal health funding and the maternal mortality rate, which were excluded due to more than 10% missing data (data not shown). The average municipal NMR was 7.69 per 1,000 live births (SD = 9.78), ranging from 0 to 142.86 per 1,000. In 2009, the rate was 9.57‰ (SD = 11.89), decreasing to 7.09‰ (SD = 8.71) in 2018 (data not shown). Descriptive analysis of the indicators included measures of central tendency and dispersion. Pearson’s correlation identified 12 indicators with *p* < 0.20, of which six were statistically significant (*p* < 0.05), as detailed in Supplementary material 8.

The pre-selected indicators were grouped based on intercorrelations (Supplementary materials 9, 10). Within each group of highly correlated indicators (r ≥ 0.50), only one was retained for multiple regression modeling to avoid multicollinearity. The multiple linear regression identified three indicators significantly associated with the neonatal mortality rate: the number of nurses in the public health system per 100,000 inhabitants (*β* = −0.028; *p* = 0.029), the number of ultrasound devices in the public health system per 100,000 inhabitants (*β* = −0.030; *p* = 0.017), and percentage of the population covered by private health insurance (β = −0.033; *p* = 0.008). The final model had a mean VIF of 1.02 and an adjusted R^2^ of 0.0023 (Table 3). For inclusion in the epidemiological risk score, these indicators were dichotomized based on the mean of each continuous variable. (Supplementary material 11). Supplementary materials 12, 13 present, respectively, the distribution of public system nurse density and private health insurance coverage across municipalities during the study period.

**Table 3:** Multiple linear regression of municipal indicators associated with neonatal mortality rate, State of São Paulo, 2009–2018.

Variable	*β*	*p*-value	95% CI (Lower)	95% CI (Upper)	VIF
Nurses in public health system per 100,000 inhabitants	−0.028	0.029	−0.052	−0.003	1.03
Ultrasound devices in public health system per 100,000 inhabitants	−0.030	0.017	−0.054	−0.005	1.02
Population covered by private health insurance (%)	−0.033	0.008	−0.058	−0.009	1.01
Mean VIF = 1,02; *R*^2^ ajustado = 0,0023					

### 3.7 Neonatal mortality risk score

For the risk score modeling, the cohort was filtered to include only records with complete information on all variables selected in the final model. After this step, a total of 5,668,011 live births across 645 municipalities in the state of São Paulo were analyzed.

Multilevel logistic regression identified 11 independent risk factors significantly associated with neonatal death. Increased risk was observed for male sex, low birth weight (< 2,500 g), maternal age < 17 or ≥ 40 years, presence and severity of congenital anomalies (scores 1 to 3), specific combinations of delivery type and gestational age (cesarean at term, preterm cesarean, and preterm vaginal birth), fewer than seven prenatal visits, birth in spring or summer, multiple pregnancy, lower nurse density in the public health system, and lower private health insurance coverage.

The model was statistically significant [Wald *χ*^2^ (14) = 126,140.39; *p* < 0.001], with better fit than the model without random effects [*χ*^2^ (1) = 833.28; *p* < 0.001]. Detailed results are presented in Table 4.

**Table 4:** Results of the multilevel logistic regression and score points assigned to variable categories for the construction of the epidemiological neonatal mortality risk score, based on odds ratio (OR) scaling, State of São Paulo, 2009–2018.

Variable	OR	*p*-value	95% CI (Lower)	95% CI (Upper)	ln (OR)	Score
Male sex	1.2491	<0.001	1.2229	1.2759	0.22	1
Birth weight <2,500 g	8.4592	<0.001	8.2091	8.7170	2.14	4
Maternal age <17 or ≥40 years	1.0733	<0.001	1.0358	1.1121	0.07	1
Congenital anomaly – Grade 1	9.1751	<0.001	8.7763	9.5920	2.22	4
Congenital anomaly – Grade 2	58.620	<0.001	55.048	62.423	4.07	7
Congenital anomaly – Grade 3	284.78	<0.001	252.62	321.03	5.65	10
Cesarean delivery, gestational age ≥37 weeks	1.2742	<0.001	1.2221	1.3285	0.24	1
Cesarean delivery, gestational age <37 weeks	5.0770	<0.001	4.8691	5.2938	1.63	4
Vaginal delivery, gestational age <37 weeks	8.4771	<0.001	8.1324	8.8363	2.14	4
<7 prenatal visits	2.7434	<0.001	2.6802	2.8082	1.01	3
Season: Spring/Summer	1.0412	<0.001	1.0195	1.0633	0.04	1
Multiple pregnancy	1.0967	<0.001	1.0601	1.1345	0.10	1
Nurse density in public system (<mean)	1.0618	<0.001	1.0305	1.0941	0.06	1
Private health insurance coverage (<mean)	1.1765	<0.001	1.1157	1.2405	0.17	1
Constant	0.0007	<0.001	0.0006	0.0007	–	–
Municipality of residence - var.(_cons)	0.0560	—	0.0442	0.0709	–	–

### 3.8 Score calculation

As described in the Methods, adjusted odds ratios (OR) were transformed using the natural logarithm and scaled to a 1–10 range with the formula: Score = ROUND(a × ln(OR) + b), where a = 1.6039 and b = 0.9353. Scores were rounded to the nearest whole number. The scale was applied to 14 conditions from 11 variables, with scores ranging from 1 to 10 (Table 4).

A score of 1 was assigned to male sex, maternal age <17 or ≥40, term cesarean, spring/summer birth, and multiple pregnancy. Municipal-level indicators—nurse density in the public health system and private health insurance coverage—were scored when values were below the state average. These were labeled as lower nurse density and lower private coverage in the score. Premature births (cesarean or vaginal) scored 4. Congenital anomalies scored 4 (grade 1), 7 (grade 2), and 10 (grade 3). Low birth weight received 4 points, and fewer than seven prenatal visits received 3. Reference categories scored zero (Table 4).

### 3.9 Score validation

#### 3.9.1 Internal validation

**Figure 1:**
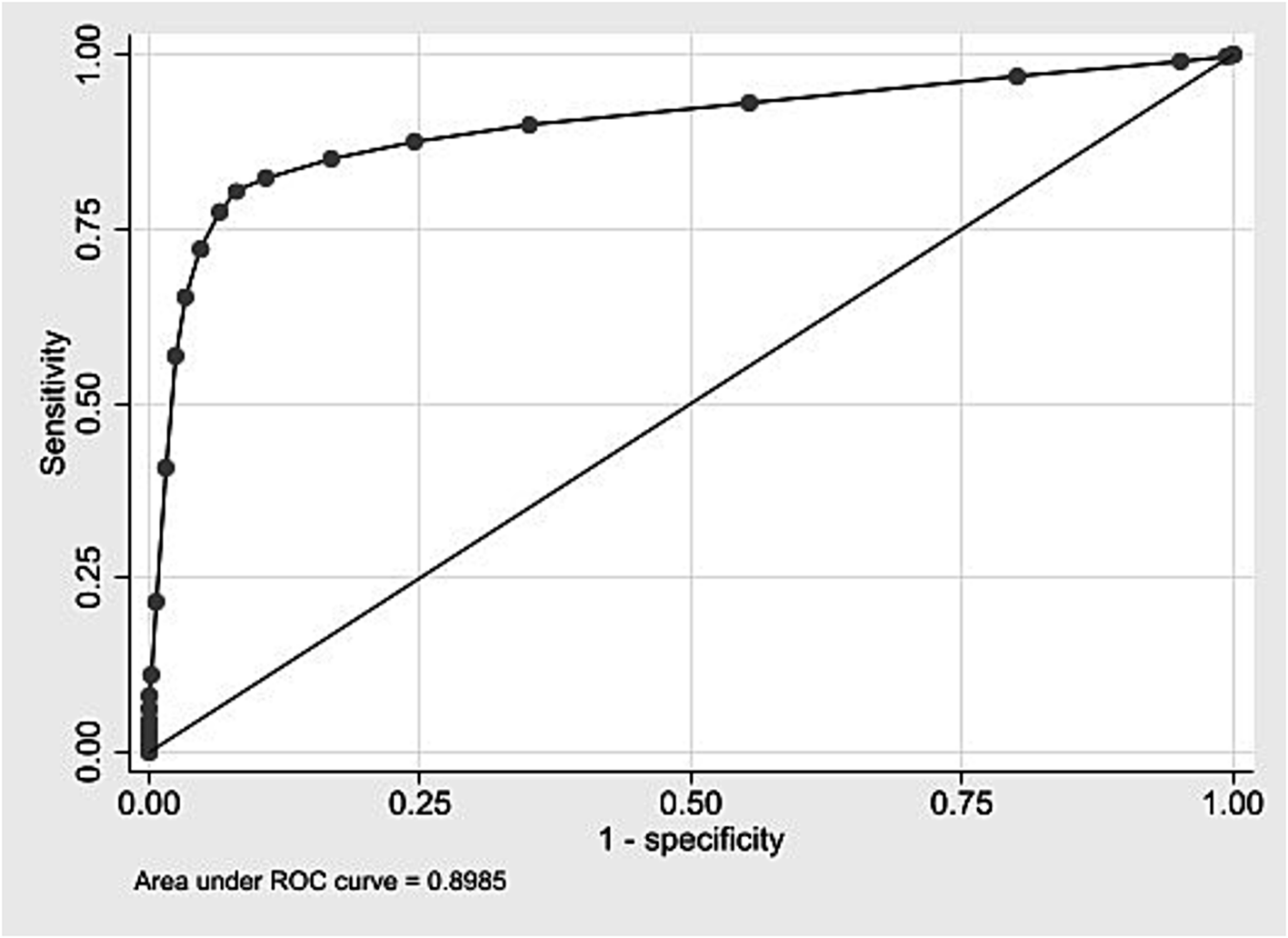
ROC curve for internal validation of the neonatal mortality risk score. State of São Paulo, 2009–2018.

In the development cohort (2009–2018), the score ranged from 0 to 26, with a mean of 4.5 (SD 2.6). The ROC curve (Figure 1) showed an AUC of 0.8985 (95% CI: 0.8965–0.9005). Supplementary material 14 presents the distribution of the risk score among live births in the internal validation cohort. At the cutoff ≥9, it showed 80.4% sensitivity, 91.9% specificity, 6.8% PPV, and 99.8% NPV (Table 5). PPVs increased with the score, exceeding 50% from score 18 onward. Odds ratios also increased, surpassing 100 from score 14 (Supplementary material 15). The logistic model had a pseudo R^2^ of 32.25%.

**Table 5:** Performance of the neonatal mortality risk score by cutoff, State of São Paulo, 2009–2018.

Cut point	Sensitivity (%)	Specificity (%)	PPV (%)	NPV (%)	Correctly Classified (%)	Youden’s index (%)
≥0	100.00	0.00	0.72	-	0.72	0.00
≥1	99.90	0.50	0.73	99.85	1.22	0.40
≥2	99.18	4.79	0.75	99.88	5.47	3.97
≥3	96.91	19.88	0.87	99.89	20.44	16.79
≥4	93.14	44.63	1.21	99.89	44.98	37.77
≥5	89.93	64.90	1.83	99.89	65.08	54.83
≥6	87.64	75.51	2.54	99.88	75.59	63.15
≥7	85.14	83.21	3.56	99.87	83.23	68.35
≥8	82.44	89.22	5.28	99.86	89.17	71.66
≥9	80.41	91.95	6.79	99.85	91.87	72.36
≥10	77.57	93.46	7.95	99.83	93.34	71.03
≥11	72.07	95.20	9.87	99.79	95.04	67.28
≥12	65.31	96.69	12.56	99.74	96.46	61.99
≥13	56.82	97.61	14.78	99.68	97.32	54.43
≥14	40.73	98.51	16.59	99.56	98.09	39.24
≥15	21.46	99.40	20.78	99.43	98.84	20.87
≥16	10.96	99.85	34.42	99.35	99.20	10.81
≥17	8.03	99.94	48.41	99.33	99.27	7.97
≥18	6.13	99.96	52.52	99.32	99.28	6.09
≥19	4.43	99.98	58.70	99.31	99.29	4.41
≥20	3.23	99.99	64.47	99.30	99.29	3.21
≥21	2.35	99.99	69.41	99.29	99.29	2.34
≥22	1.34	100.00	74.32	99.29	99.28	1.34
≥23	0.83	100.00	82.28	99.28	99.28	0.83
≥24	0.48	100.00	84.26	99.28	99.28	0.48
≥25	0.19	100.00	85.56	99.28	99.28	0.19
>25	0.03	100.00	100.00	99.28	99.28	0.03

**Figure 2:**
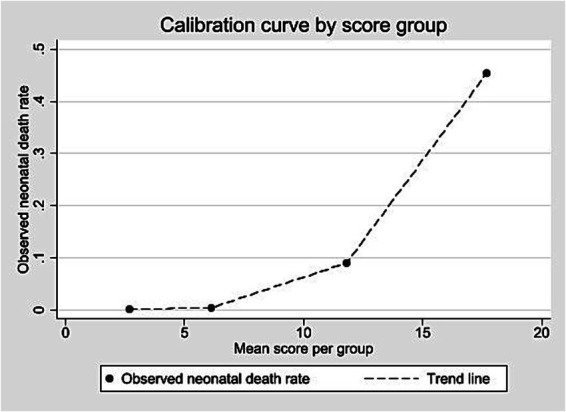
Calibration curve according to score group – internal validation, State of São Paulo, 2009–2018.

Scores were grouped into four risk categories. The 0–4 range included 64.5% of live births and 0.11% of neonatal deaths (PPV up to 1.2%). The 5–9 range covered 28.4% of births and 0.31% of deaths (PPV up to 6.8%). The 10–15 group represented 6.8% of births and 7.06% of deaths (PPV up to 20.8%). The highest-risk group (≥16) comprised 0.23% of births and accounted for 34.42% of deaths (PPV above 34.4%). Table 6 shows the classification of live births into four risk groups based on the score distribution, along with neonatal deaths and predictive values. The calibration curve (Figure **2**) showed rising neonatal death rates with higher scores.

**Table 6:** Proposal for classifying live births into risk groups using the score – internal validation, State of São Paulo, 2009–2018.

Group	Score	Live Births (N)	Live Births (%)	PPV (%)	Neonatal Deaths (N)	%
1 - Very Low Risk	0 to 4	3,655,902	64.50	0.7 to 1.2	4,129	0,11
2 - Low to Moderate Risk	5 to 9	1,612,024	28.44	1.8 to 6.8	5,067	0,31
3 - High Risk	10 to 15	387,030	6.83	8.0 to 20.8	27,316	7,06
4 - Very High Risk	≥16	13,055	0.23	≥34.4	4,493	34,42
Total		5,668,011	100.00	–	41,005	0,72

#### 3.9.2 External validation

**Figure 3:**
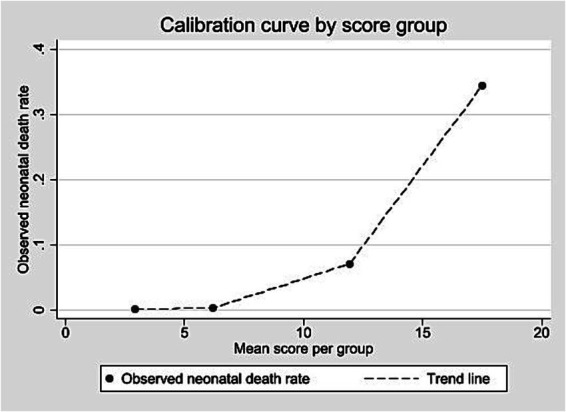
ROC curve for external validation of the neonatal mortality risk score. State of São Paulo, 2008.

**Figure 4:**
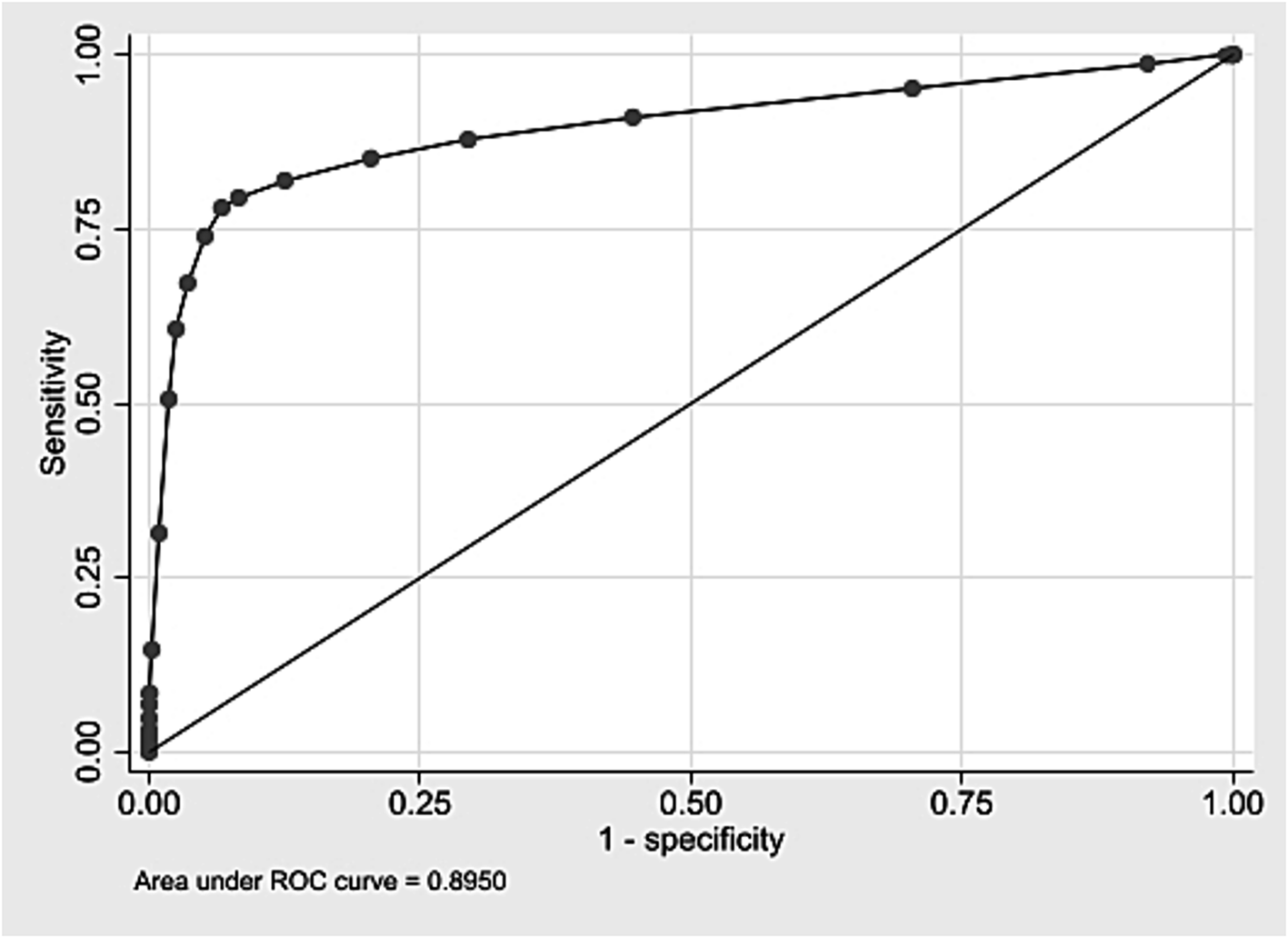
Calibration curve according to score group – external validation, State of São Paulo, 2008.

External validation was conducted using a cohort of 508,237 live births in São Paulo in 2008. Score values ranged from 0 to 25, with a median of 3 and an interquartile range of 2–5 (data not shown). Most births were concentrated between scores 2 and 4 (Supplementary material 16). Discrimination was assessed using the ROC curve (Figure **3**), with an AUC of 0.8950 (95% CI: 0.8884–0.9017). At the predefined cut-off ≥9, sensitivity was 78.0% and specificity 93.4%; positive and negative predictive values were 8.3 and 99.8%, respectively (Table 7). The logistic regression model using the score yielded a pseudo R^2^ of 31.5% (data not shown). Supplementary material 17 presents the number of neonatal deaths by score value and corresponding odds ratios. Higher scores were associated with increasing predictive values mortality proportions, and odds ratios (Table 7; Supplementary material 17). Grouped score categories showed a progressive concentration of neonatal deaths: scores 0–4 included 70.1% of births but only 0.13% of deaths; scores 5–9 included 24.2% of births and 0.43% of deaths; scores 10–15 included 5.5% of births and 9.03% of deaths; and scores ≥16, just 0.14% of births, accounted for 45.35% of neonatal deaths (Table 8). Calibration was assessed by comparing observed mortality across score levels, showing increasing proportions with higher scores (Figure 4).

**Table 7:** Performance measures of the neonatal mortality risk score by different cut-off points – external validation, State of São Paulo, 2008.

Cut point	Sensitivity (%)	Specificity (%)	PPV (%)	NPV (%)	Correctly classified (%)	Youden’s index (%)
> = 1	99.97	0.55	0.77	99.96	1.31	0.52
> = 2	98.84	7.87	0.82	99.89	8.56	6.71
> = 3	95.22	29.58	1.03	99.88	30.08	24.80
> = 4	91.15	55.38	1.54	99.88	55.65	46.53
> = 5	87.84	70.52	2.24	99.87	70.65	58.36
> = 6	85.26	79.51	3.10	99.86	79.55	64.77
> = 7	82.03	87.52	4.81	99.84	87.48	69.56
> = 8	79.53	91.78	6.92	99.83	91.69	71.31
> = 9	78.03	93.36	8.28	99.82	93.24	71.39
> = 10	74.03	94.84	9.93	99.79	94.69	68.88
> = 11	67.22	96.50	12.85	99.74	96.28	63.72
> = 12	60.69	97.48	15.60	99.69	97.20	58.17
> = 13	50.67	98.16	17.45	99.62	97.80	48.83
> = 14	31.39	99.06	20.48	99.47	98.55	30.45
> = 15	14.69	99.73	29.36	99.35	99.08	14.42
> = 16	8.44	99.92	45.35	99.30	99.22	8.36
> = 17	6.71	99.96	54.39	99.29	99.25	6.67
> = 18	4.75	99.98	60.33	99.27	99.25	4.73
> = 19	3.12	99.99	66.85	99.26	99.25	3.11
> = 20	2.27	99.99	70.97	99.25	99.25	2.26
> = 21	1.39	100.00	75.00	99.25	99.24	1.39
> = 22	0.70	100.00	79.41	99.24	99.24	0.70
> = 23	0.39	100.00	93.75	99.24	99.24	0.39
> = 24	0.23	100.00	100.00	99.24	99.24	0.23
>24	0.05	100.00	100.00	99.24	99.24	0.05

**Table 8:** Classification of live births by neonatal mortality risk groups – external validation, State of São Paulo, 2008.

Group	Score	Live Births (N)	Live Births (%)	PPV (%)	Neonatal Deaths (N)	%
1 - Very Low Risk	0 to 4	356,156	70.08	0.8 to 1.6	471	0.13
2 - Low to Moderate Risk	5 to 9	123,209	24.24	2.2 to 8.3	535	0.43
3 - High Risk	10 to 15	28,151	5.54	9.9 to 29.4	2,541	9.03
4 - Very High Risk	≥16	721	0.14	≥45.4	327	45.35
Total		508,237	100.00		3,874	0.76

## 4 Discussion

This study, conducted in a middle-income country setting, identified 11 factors associated with neonatal death at both individual and municipal levels in the state of São Paulo (2009–2018). A risk score was developed based on these factors using a large population-based cohort of over 5.6 million live births, with data from birth certificates (Fundação SEADE) and municipal health indicators (PROADESS). Congenital anomalies were classified by severity, considering frequency and lethality.

The score includes nine individual variables and two municipal indicators: male sex, birth weight <2,500 g, maternal age <17 or ≥40 years, fewer than 7 prenatal visits, vaginal or cesarean delivery before 37 weeks, cesarean delivery at term (≥37 weeks), multiple pregnancy, birth during spring or summer, congenital anomaly with severity grade (1–3, or), lower nurse density in the public health system, and lower private health insurance coverage.

Designed as an epidemiological score, it integrates both individual and contextual factors, reflecting not only biological vulnerability but also healthcare and structural aspects of the local health system. This aligns with the use of risk scores as technical tools for collective health planning (52).

### 4.1 Risk score development

The risk score was developed using multilevel logistic regression, an extension of multiple logistic regression that accounts for hierarchical data structure (individuals nested within municipalities). Multiple logistic regression is widely used in neonatal mortality prediction studies due to its transparency, lower complexity, and clinical interpretability (53). Estimated coefficients [ln(OR)] were proportionally scaled to a 1–10 range using observed minimum and maximum ln(OR) values, reflecting each variable’s association strength with neonatal death. This approach aligns with established methods for converting logistic regression coefficients into proportional scoring systems (54–56).

The multilevel model integrated individual and contextual variables, acknowledging that individuals are grouped by municipality. This allowed estimation of the territory’s effect on neonatal mortality risk and captured unexplained differences between municipalities. A statistically significant random intercept indicated that birthplace influenced outcomes even after controlling for individual characteristics, justifying context-aware models (42).

The development cohort included 5.5 million live births in São Paulo (2009–2018), with over 40,000 neonatal deaths (rate: 7–8 per 1,000 births), consistent with national estimates. The large size of this dataset represents a methodological strength, especially considering that many studies rely on substantially smaller samples. Including a high number of births and deaths supports the consistency of findings and enhances reproducibility. Smaller samples are a well-documented limitation in predictive modeling, as they may compromise model validity. In this sense, the use of a large population-based cohort aligns with current methodological recommendations (57, 58).

External validation used a retrospective temporal cohort (2008 data), ensuring chronological independence between development and validation samples. This practice minimizes overfitting risks and tests model generalizability, as demonstrated in prior studies (54, 56).

The study adhered to TRIPOD guidelines for transparent reporting of predictive models, detailing population, predictors, outcomes, statistical methods, external validation, and performance evaluation (59). Records with incomplete data were excluded without imputation.

Predictive scores, often derived from cohort or case–control designs, translate complex statistical models into interpretable tools for public health. This study employed a cohort design, contrasting with case–control approaches used elsewhere (54, 56).

### 4.2 .Comparison with other neonatal mortality risk scores

The risk score developed in this study differs fundamentally from clinical neonatal scores (e.g., Clinical Risk Index for Babies [CRIB], CRIB-II, Score for Neonatal Acute Physiology with Perinatal Extension-II [SNAPPE-II], and neonatal Sequential Organ Failure Assessment [nSOFA]) in purpose, variables, and context. While clinical scores rely on dynamic physiological data (e.g., blood pH, blood pressure, PaO_2_/FiO_2_ ratio) and specialized NICU monitoring, the proposed score is static, using only variables from Live Birth Certificates and municipal health indicators (e.g., nurse density, private health insurance coverage). This design allows immediate application after birth, even outside hospital settings (25–28, 31).

Although it shares some variables with clinical scores (e.g., birth weight, sex, congenital anomalies), this model uniquely incorporates non-clinical factors such as delivery type, prenatal care visits, maternal age, and municipal health system performance. For example, clinical scores include parameters like urine output, seizures, or platelet counts, which require laboratory tests and intensive care monitoring. In contrast, this score excludes such variables, prioritizing simplicity and scalability for population-level use (25–28).

By avoiding reliance on clinical exams or specialized infrastructure, the score offers a feasible tool for large-scale neonatal risk stratification. While clinical scores focus on individual patient management in NICUs, this model aims to classify risk immediately after birth using nationally available data, bridging a critical gap in public health surveillance (25–28).

### 4.3 Performance and validation of the neonatal mortality risk score

The score showed similar results in identifying the risk of neonatal death in both the development and external validation cohorts, with AUC values close to 0.90. The best threshold (score ≥9), based on the Youden index, had 80.4% sensitivity and 91.9% specificity in the development cohort, and 78.0% sensitivity and 93.4% specificity in the validation cohort (44, 45).

Although neonatal death was a rare event (~0.8%), the model reached a high negative predictive value (NPV) (99.8%) in both cohorts. The chance of correctly predicting a death (PPV) increased with higher scores: 6.8% in the development cohort and 8.3% in the validation cohort for scores ≥9, surpassing 30% in the highest score ranges. This pattern is expected in the prediction of rare outcomes, where NPV tends to be high and PPV more modest, even when the model shows good discriminatory performance. As described in the literature, predictive values vary with outcome prevalence, unlike other metrics such as positive and negative likelihood ratios (LR + and LR–), which may be useful in estimating individual probabilities in prediction-focused studies (46). In this study, PPV was used to support risk stratification by ranking the probability of neonatal death across score ranges. Metrics typically used in individual-level prediction were thus applied to develop a population-based risk classification.

Dividing the score into four risk categories helped show risk levels. In the lowest category (0–4 points), the chance of death was about the same as the average rate, meaning the score did not add much value there. But in the higher categories, the chance of death increased clearly, and this pattern appeared in both datasets. This shows that the score works in different populations, including the smaller validation cohort (~508,000 births). The score thresholds can be adjusted depending on the goal: lower thresholds are better for finding more possible cases, while higher ones are better for focusing on the babies most at risk.

### 4.4 Performance comparison with other scores

The score developed in this study showed AUC values close to 0.90, comparable to those reported for other neonatal mortality scores. CRIB showed an AUC of 0.877 in a study with preterm newborns, while CRIB-II and SNAPPE-II showed values ranging from 0.79 to 0.90. Another study reported AUCs of 0.89 for SNAPPE-II and 0.87 for CRIB. These values are similar to those observed in both the development and external validation of the proposed score. Additionally, AUCs reported for models using machine learning, such as neural networks and random forest, ranged from 58.3 to 97.0% (25–28, 53, 60).

### 4.5 Score components

#### 4.5.1 Sex

Male sex was included in the score due to its consistent association with higher neonatal mortality, particularly among preterm and low birth weight newborns. This pattern has been observed across different countries and income levels. In Brazil, studies with opposite-sex twins found higher mortality rates among boys, as well as greater frequency of low Apgar scores and congenital anomalies. The literature relates this vulnerability to slower lung maturation and higher risk of respiratory and neurological complications (6, 61–66).

#### 4.5.2 Birth weight

Low birth weight (<2,500 g) is a widely recognized risk factor for neonatal death and was included in the score. Within this group, very low birth weight (≤1,500 g) represents a subgroup with markedly higher risk, often concentrated among the most severe clinical cases. These findings support the clinical and epidemiological value of the selected cutoff and highlight the need to consider heterogeneity within this category (6, 66–69).

#### 4.5.3 Maternal age

Maternal age <17 or ≥40 years was included as a risk factor. While some studies consider ≥35 years, the evidence suggests that risk increases more clearly from age 40. Among adolescents, risk rises especially before age 16, with regional variation. Large multicenter studies confirm higher perinatal and neonatal mortality at the extremes of maternal age, and low maternal education may exacerbate this risk (70–73).

#### 4.5.4 Congenital malformations, deformities, and chromosomal anomalies

The presence of congenital anomalies was one of the main factors associated with neonatal death, as also reported in several previous studies (6, 66). In the Brazilian context, there is a national list focused on the surveillance of anomalies that can be diagnosed at birth and have potential for intervention (74). The classification proposed in this study for neonates with one or more anomalies complements this surveillance effort by introducing a severity criterion based on the observed impact on neonatal mortality. This criterion was constructed based on the proportion of neonatal deaths by ICD-10 category (Chapter XVII – Q00–Q99) (37), and is useful for analytical purposes and risk stratification.

#### 4.5.5 Type of delivery and gestational age

The combined variable of gestational age and delivery type was included in the score, with term vaginal delivery (≥37 weeks) as the reference. Preterm births, especially preterm vaginal deliveries, received higher scores due to their strong association with neonatal mortality. Prior studies showed cesarean section reduced neonatal mortality between 22 and 31 weeks but increased risk at 32–41 weeks. Gestational age remains the main predictor of early neonatal death in Brazil. Both national and international research indicate cesarean may lower neonatal morbidity and mortality in preterm births, particularly for breech presentations or <34 weeks, though such details were not available in this dataset (38, 75–77).

In this cohort, 52.9% of births were term cesareans, 36.6% term vaginal, and 10.5% preterm (cesarean 6.5%, vaginal 4.0%). High cesarean rates are seen nationwide; the “Nascer no Brasil” study found a 45.5% prevalence among low-risk pregnancies. Globally, rising cesarean use - even without clinical indication - coexists with limited access in some regions, both contributing to preventable mortality and resource waste. The WHO recommends cesarean only when clinically indicated, including for preterm births, as indiscriminate use may increase neonatal and maternal morbidity, especially where resources are limited. National guidelines stress the need for adequate birth care infrastructure, including skilled neonatal resuscitation, particularly for preterm infants (6, 78–81).

#### 4.5.6 Prenatal care

Inadequate prenatal care, defined as fewer than seven visits, was associated with higher neonatal mortality and included in the score. National recommendations suggest about 14 visits for term pregnancies. International studies show that even a single qualified visit can reduce risk, especially in low-resource settings (6, 66, 82–84).

#### 4.5.7 Season of birth

The inclusion of “birth in spring or summer” in the score was associated with a small but consistent increase in neonatal mortality risk, even after adjusting for sociodemographic and contextual factors. Two main explanations are proposed. First, exposure to high temperatures during early pregnancy may affect fetal development, particularly increasing the risk of congenital heart defects. This has been suggested by U. S. and Swedish studies, with higher vulnerability observed among male infants. Second, seasonal viral infections—more frequent during spring and summer—may also contribute to the increased risk, as shown by recent data linking respiratory infections to child mortality. Although the present study lacked data on environmental temperature or viral circulation, the association may reflect unmeasured exposures. Future studies integrating environmental data and health service availability could help clarify these seasonal effects (85–87).

#### 4.5.8 Multiple pregnancy

Multiple gestations were associated with increased neonatal mortality and were included in the score. This relationship is well-documented in both high-income and low-resource settings (66, 67, 69, 82).

#### 4.5.9 Municipal-level variables

Two municipal-level indicators were included in the score: the number of nurses working in the public health system per 100,000 inhabitants and the percentage of private health insurance coverage. These indicators were calculated using data from the National Register of Health Facilities (CNES, acronym in Portuguese), IBGE, the National Household Sample Survey (PNAD, acronym in Portuguese), and the National Health Survey (PNS, acronym in Portuguese) (35).

Health insurance coverage serves as a proxy for socioeconomic status. However, only 11.7% of women of reproductive age have coverage for childbirth, and many plans exclude hospital care (88).

In this study, a higher municipal-level density of nurses in the public health system was associated with lower neonatal mortality. Other studies have also identified an association between greater numbers of nursing professionals and lower neonatal mortality rates, especially when adequate training and continuous presence in care units are ensured (89–91).

Both indicators were part of a broader set of variables significantly associated with neonatal mortality, with important correlations observed among human resource indicators, suggesting that other healthcare professionals may also influence the outcome.

### 4.6 Strengths

The development of risk scores has gained prominence with the advancement of electronic health records and the increased availability of large datasets. In this study, a broad population-based cohort of approximately 5.6 million live births was used to construct the neonatal mortality risk score, with a subset employed to classify the severity of congenital anomalies. The use of data from Fundação SEADE (29), obtained from Live Birth Declarations and linked to neonatal death records, enabled the study due to its availability and coverage in the state of São Paulo (33, 34).

The proposed score presents important advantages in terms of applicability, as it can be calculated using individual information routinely collected at birth, without the need for specialized tests or complex hospital infrastructure, and incorporates contextual variables from public databases. The PROADESS platform provides municipality-level data for the entire country, which can be used to adapt the score to other states (35).

### 4.7 Limitations and future directions

Despite the use of a large dataset in the development of the score and the satisfactory performance in terms of discrimination and calibration for neonatal death risk, some methodological limitations and opportunities for improvement should be considered.

#### 4.7.1 Limitation of anomaly classification

An important limitation is that the classification of congenital anomalies was based on an empirical approach using the proportion of neonatal deaths observed among live births within each ICD-10 category. This classification and the grading logic were subsequently reviewed to ensure clinical coherence and biological plausibility. The objective was to represent gradients of severity among different types of anomalies in an epidemiologically interpretable and biologically coherent manner, avoiding dichotomization.

This approach involves partial methodological circularity, since the outcome (neonatal death) was used both to define severity and to model risk, with both derived from the same live birth cohort. To minimize possible effects of this dependence, only isolated anomalies were considered, and the most severe grade was assigned per individual to prevent artificial summation of severity. However, the classification did not account for the interaction between multiple malformations, which may limit the assessment of severity in neonates presenting more than one anomaly.

This strategy addressed an analytical need in the absence of standardized severity classifications applicable to large population-based datasets. The score also depends on accurate anomaly recording by healthcare professionals, and less frequent categories were underexplored, which may limit the representativeness of risk (Supplementary material 7). For these categories, additional studies are needed to better estimate their association with neonatal mortality (37). Further refinement by subcategory and outcome-based analyses could improve the severity classification, considering the heterogeneity within broad ICD-10 categories.

In a broader sense, the severity of congenital anomalies also relates to survival with sequelae, prolonged dependence on medical care, use of health technologies, and the socioeconomic impacts on families and healthcare systems. Although developed within the same cohort used for model construction, the score showed similar performance in external validation, indicating consistency in representing the risk associated with congenital anomalies.

#### 4.7.2 Potential for ecological fallacy in individual risk attribution

Although the score was derived from a multilevel model, the coefficients of contextual variables (e.g., nurse density) were converted into points and assigned equally to all individuals in the same municipality, as if all were equally exposed to the average conditions of that location. This simplification may lead to ecological fallacy, that is, the error of assuming that aggregated data (municipal-level) represent individual reality, ignoring internal variations among residents of the same area (92, 93).

This risk does not invalidate the use of contextual variables in the score’s construction but requires cautious interpretation. The points assigned based on these variables should be understood as contextual approximations of the individual’s environment, not as direct measures of their actual exposure or individual risk (93).

#### 4.7.3 Other limitations and future directions

The univariate model showed a pseudo *R*^2^ of 32.25% in the development cohort and 31.5% in external validation, values comparable to established scores like CRIB (28–35%). These results highlight the complexity of the outcome and the presence of unexplained variability (26, 28, 60). The risk estimate generated is limited to the included variables and does not represent an absolute risk. Unmeasured factors, such as the quality of perinatal care, may influence the outcome and could be incorporated into future versions of the score, especially those related to perinatal care and the quality of healthcare services, for example, characteristics of the healthcare facility, such as physical resources (infrastructure, equipment, and beds) and other relevant aspects.

The absence of the CNES code prevented the inclusion of information on healthcare facilities, such as service type or care structure. This data could strengthen future analyses and help reduce the risk of ecological fallacy (6, 93, 94).

The use of secondary data involves risks of underreporting and variability in data quality. Key variables, such as maternal education, marital status, and Apgar scores, were excluded due to high rates of missing data, limiting the model’s explanatory power. Studies using data from the Live Birth Information System (SINASC, acronym in Portuguese) indicate that socioeconomic variables still exhibit low completeness and reliability (31, 32, 73, 94, 95).

### 4.8 Applications

The classification of live births into risk strata based on the proposed score enables applications in clinical practice, public health surveillance, and healthcare management. The score stratifies newborns according to their risk of neonatal death and can be used for screening, clinical protocols, as an objective criterion for inclusion or exclusion in scientific studies, and as a complement to clinical evaluation— especially in limited-resource settings in middle-income countries. Since it was developed from a comprehensive population-based dataset, it reflects the distribution of live births by risk levels in the state of São Paulo. This allows for the comparison of risk profiles between municipalities, regions, or hospitals; the identification of priority areas for intervention; the monitoring of trends over time; and the assessment of public health initiatives. The identification of deaths among newborns classified as low risk may also indicate preventable failures in care. In summary, the score supports clinical decision-making, guides surveillance actions, improves mortality audits, and strengthens protocols and public policies aimed at reducing neonatal mortality.

### 4.9 Model reproducibility in other contexts

The score was developed using high-quality population-based data from the state of São Paulo, where the completeness and reliability of the Live Birth (DNV) and Death Certificates (DO) are among the best in Brazil. Its direct application is appropriate only in contexts with comparable data structure and quality, similar epidemiological profiles, equivalent health system performance, and matching levels of human development (HDI-M) and neonatal mortality rates (NMR). Under these conditions, it is assumed that the risk determinants and their respective weights would behave similarly. This scenario is observed in states such as Santa Catarina, Paraná, and the Federal District, which show development and neonatal mortality patterns close to those of São Paulo (16, 18).

In states with higher mortality rates or marked differences in health-service structure and data quality — as still found in parts of the North and Northeast regions — local validation is recommended to verify whether the model’s performance metrics remain equivalent. This step may include adjusting the variable weights if the magnitude of associations differs substantially from those estimated for São Paulo.

Validation is technically feasible through linkage between the Live Birth (DNV) and Death (DO) Certificates, available in the national information systems (SINASC and SIM), which allows reconstruction of comparable population-based cohorts at the state level. The linkage became operationally possible after the inclusion, in the Death Certificate, of a specific field for the DNV number — mandatory for deaths of children under 1 year of age — as established in the Ministry of Health’s Instruction Manual (32).

Thus, the proposed methodology can be applied in other settings, allowing the review and adjustment of individual and contextual factors according to the availability and quality of local data. This approach highlights the possibility of reproducing the analytical process while maintaining the conceptual coherence of the model, without assuming its automatic generalization.

## 5 Conclusion

The proposed score integrates routinely collected individual birth data with municipal-level indicators to stratify the risk of neonatal death. This stratification supports the prioritization of care, the allocation of resources, and the implementation of preventive measures focused on the most vulnerable groups. The score can be applied both in clinical practice and in population-based surveillance, including the geographic monitoring of birth-risk distribution and the screening of deaths among newborns classified as low risk, thereby contributing to audit strategies and to the continuous improvement of neonatal care quality. Because it relies exclusively on information already available in official databases, the score enables retrospective, large-scale use. Finally, its application should complement — not replace — individualized clinical judgment, always considering the specific characteristics of each newborn and the local context of care.

## Data Availability

The data analyzed in this study is subject to the following licenses/restrictions: The dataset used in this study is not publicly available. Access is restricted and was granted under institutional agreements between Fundação SEADE and the Federal University of São Paulo (UNIFESP). Data use was authorized solely for the purposes of this research. Requests for access must be directed to Fundação SEADE (https://www.seade.gov.br).
