# Supplementary material for: Development and validation of an epidemiological risk score for neonatal death in a middle-income country
Source: Front Public Health. 2025 Nov 19;13:1675040. doi: 10.3389/fpubh.2025.1675040 (PMC12672502; doi:10.3389/fpubh.2025.1675040)
Supplement: Supplementary file 1 [file Table_1.docx]

### ****Supplementary Material 1.** Annual distribution of integrated records of live births, infant deaths, and neonatal deaths among children of mothers residing in the State of São Paulo, 2009–2018.**

| **Year of birth** | **Live births** | **Infant deaths** | **Neonatal deaths** | **Neonatal deaths among live births** |
| --- | --- | --- | --- | --- |
| 2009 | 598,909 | 7,522 | 5,192 | 5,184 |
| 2010 | 601,561 | 6,959 | 4,834 | 4,820 |
| 2011 | 610,492 | 6,633 | 4,444 | 4,371 |
| 2012 | 617,084 | 6,962 | 4,846 | 4,808 |
| 2013 | 611,300 | 7,047 | 4,783 | 4,780 |
| 2014 | 625,750 | 6,914 | 4,820 | 4,819 |
| 2015 | 632,407 | 6,741 | 4,717 | 4,713 |
| 2016 | 599,950 | 6,285 | 4,500 | 4,500 |
| 2017 | 611,541 | 6,669 | 4,610 | 4,610 |
| 2018 | 605,630 | 6,396 | 4,439 | 4,439 |
| Total | 6,114,624 | 68,128 | 47,185 | 47,044 |
