# Supplementary material for: Development and validation of an epidemiological risk score for neonatal death in a middle-income country
Source: Front Public Health. 2025 Nov 19;13:1675040. doi: 10.3389/fpubh.2025.1675040 (PMC12672502; doi:10.3389/fpubh.2025.1675040)
Supplement: Supplementary file 2 [file Table_2.docx]

### Supplementary Material 2. Annual distribution of live births and neonatal deaths by availability of maternal municipality of residence. State of São Paulo, 2009–2018.

| **Year of Birth** | **Municipality of residence not reported** | | |  | **Municipality of residence reported** | |
| --- | --- | --- | --- | --- | --- | --- |
|  | **Live births (N)** | **Neonatal death** | **%*** |  | **Live births** | **Neonatal deaths** |
| 2009 | 52 | 0 | 0.01 |  | 598,857 | 5,184 |
| 2010 | 57 | 0 | 0.01 |  | 601,504 | 4,820 |
| 2011 | 11 | 0 | 0.00 |  | 610,481 | 4,371 |
| 2012 | 53 | 1 | 0.01 |  | 617,031 | 4,807 |
| 2013 | 74 | 0 | 0.01 |  | 611,226 | 4,780 |
| 2014 | 68 | 2 | 0.01 |  | 625,682 | 4,817 |
| 2015 | 335 | 2 | 0.05 |  | 632,072 | 4,711 |
| 2016 | 286 | 2 | 0.05 |  | 599,664 | 4,498 |
| 2017 | 279 | 1 | 0.05 |  | 611,262 | 4,609 |
| 2018 | 231 | 0 | 0.04 |  | 605,399 | 4,439 |
| Total | 1,446 | 8 | 0.02 |  | 6,113,178 | 47,036 |

* Percentages refer to neonatal deaths among live births with missing residence data.
