# Supplementary material for: Development and validation of an epidemiological risk score for neonatal death in a middle-income country
Source: Front Public Health. 2025 Nov 19;13:1675040. doi: 10.3389/fpubh.2025.1675040 (PMC12672502; doi:10.3389/fpubh.2025.1675040)
Supplement: Supplementary file 3 [file Table_3.docx]

### Supplementary Material 3. Missing data for selected individual variables among live births with maternal municipality reported. State of São Paulo, 2009–2018.

| **Variable** | **N** | **%** |
| --- | --- | --- |
| Child’s sex | 99 | <0.002 |
| Birth weight | 57,100 | 0.93 |
| Mother’s age | 1,096 | 0.02 |
| Gestational age | 223,327 | 3.65 |
| Number of prenatal care visits | 225,137 | 3.68 |
| Type of pregnancy | 1,526 | 0.03 |
| Type of delivery | 25,839 | 0.42 |
| Presence of congenital malformation and/or chromosomal abnormality | 265,422 | 4.34 |
| Date of birth | 208 | <0.003 |
| Municipality of birth | 0 | 0.00 |
