# Supplementary material for: Development and validation of an epidemiological risk score for neonatal death in a middle-income country
Source: Front Public Health. 2025 Nov 19;13:1675040. doi: 10.3389/fpubh.2025.1675040 (PMC12672502; doi:10.3389/fpubh.2025.1675040)
Supplement: Supplementary file 4 [file Table_4.docx]

### Supplementary Material 4. Annual distribution of neonatal deaths among live births with reported maternal municipality. State of São Paulo, 2009–2018.

| Year of Birth |  | Neonatal Death = No | Neonatal Death = Yes | **Total** |
| --- | --- | --- | --- | --- |
| 2009 | N | 593,673 | 5,184 | 598,857 |
|  | % | 99.13 | 0.87 | 100.00 |
| 2010 | N | 596,684 | 4,820 | 601,504 |
|  | % | 99.20 | 0.80 | 100.00 |
| 2011 | N | 606,110 | 4,371 | 610,481 |
|  | % | 99.28 | 0.72 | 100.00 |
| 2012 | N | 612,224 | 4,807 | 617,031 |
|  | % | 99.22 | 0.78 | 100.00 |
| 2013 | N | 606,446 | 4,780 | 611,226 |
|  | % | 99.22 | 0.78 | 100.00 |
| 2014 | N | 620,865 | 4,817 | 625,682 |
|  | % | 99.23 | 0.77 | 100.00 |
| 2015 | N | 627,361 | 4,711 | 632,072 |
|  | % | 99.25 | 0.75 | 100.00 |
| 2016 | N | 595,166 | 4,498 | 599,664 |
|  | % | 99.25 | 0.75 | 100.00 |
| 2017 | N | 606,653 | 4,609 | 611,262 |
|  | % | 99.25 | 0.75 | 100.00 |
| 2018 | N | 600,960 | 4,439 | 605,399 |
|  | % | 99.27 | 0.73 | 100.00 |
| Total | N | 6,066,142 | 47036 | 6,113,178 |
|  | % | 99.23 | 0.77 | 100.00 |
