# Supplementary material for: Development and validation of an epidemiological risk score for neonatal death in a middle-income country
Source: Front Public Health. 2025 Nov 19;13:1675040. doi: 10.3389/fpubh.2025.1675040 (PMC12672502; doi:10.3389/fpubh.2025.1675040)
Supplement: Supplementary file 5 [file Table_5.docx]

**Supplementary Material 5.** Absolute and relative frequencies of categorical variables, and summary statistics for numerical variables selected for analysis. State of São Paulo, 2009–2018.

| **Variable** | | **Category** | | | **N** | | **%** | |  |
| --- | --- | --- | --- | --- | --- | --- | --- | --- | --- |
| Child’s sex | | 1 - Male | | | 3,129,933 | | 51.2 | |  |
|  | | 2 - Female | | | 2,983,146 | | 48.8 | |  |
|  | | 3 - Undetermined | | | 99 | | 0.0 | |  |
|  | | Total | | | 6,113,178 | | 100.0 | |  |
| Prenatal care visits | | 1 - None | | | 49,714 | | 0.8 | |  |
|  | | 2 - 1 to 3 visits | | | 220,860 | | 3.8 | |  |
|  | | 3 - 4 to 6 visits | | | 1,026,177 | | 17.4 | |  |
|  | | 4 - 7 or more visits | | | 4,591,290 | | 78.0 | |  |
|  | | Total | | | 5,888,041 | | 100.0 | |  |
| Gestational age | | 1 - Less than 22 weeks | | | 2,277 | | 0.1 | |  |
|  | | 2 - 22 to 27 weeks | | | 30,368 | | 0.5 | |  |
|  | | 3 - 28 to 31 weeks | | | 59,643 | | 1.0 | |  |
|  | | 4 - 32 to 36 weeks | | | 523,403 | | 8.9 | |  |
|  | | 5 - 37 to 41 weeks | | | 5,207,111 | | 88.4 | |  |
|  | | 6 - 42 weeks or more | | | 67,049 | | 1.1 | |  |
|  | | Total | | | 5,889,851 | | 100.0 | |  |
| Type of pregnancy | | 1 - Singleton | | | 5,965,570 | | 97.6 | |  |
|  | | 2 - Twin | | | 142,007 | | 2.3 | |  |
|  | | 3 - Triplet or more | | | 4,075 | | 0.1 | |  |
|  | | Total | | | 6,111,652 | | 100.0 | |  |
| Type of delivery | | 1 - Vaginal | | | 2,481,066 | | 40.8 | |  |
|  | | 2 - Cesarean | | | 3,606,273 | | 59.2 | |  |
|  | | Total | | | 6,087,339 | | 100.0 | |  |
| Presence of congenital malformation or chromosomal anomaly | | Yes | | | 60,120 | | 1.0 | |  |
|  | | No | | | 5,787,636 | | 99.0 | |  |
|  | | Total | | | 5,847,756 | | 100.0 | |  |
|  | | **Numerical variables** | | |  | |  | |  |
|  | **N** | | **Mean** | **Std. dev** | | **Min** | | **Max** | |
| Mother's age | 6,112,082 | | 27.2 | 6.6 | | 10 | | 65 | |
| Birth weight (g) | 6,056,078 | | 3,149.4 | 547.9 | | 140 | | 6,741 | |
