# Supplementary material for: Development and validation of an epidemiological risk score for neonatal death in a middle-income country
Source: Front Public Health. 2025 Nov 19;13:1675040. doi: 10.3389/fpubh.2025.1675040 (PMC12672502; doi:10.3389/fpubh.2025.1675040)
Supplement: Supplementary file 6 [file Table_6.docx]

**Supplementary Material 6.** Distribution of the number of congenital anomalies reported at birth. State of São Paulo, 2009–2018.

| **Number of anomalies** | **N** | **%** |
| --- | --- | --- |
| 1 | 46,102 | 84.0 |
| 2 | 5,675 | 10.3 |
| 3 | 1,731 | 3.2 |
| 4 | 793 | 1.4 |
| 5 | 412 | 0.8 |
| 6 | 101 | 0.2 |
| 7 | 45 | 0.1 |
| 8 | 14 | 0.0 |
| 9 | 8 | 0.0 |
| 10 | 2 | 0.0 |
| Total | 54,883 | 100.0 |
