# Supplementary material for: Development and validation of an epidemiological risk score for neonatal death in a middle-income country
Source: Front Public Health. 2025 Nov 19;13:1675040. doi: 10.3389/fpubh.2025.1675040 (PMC12672502; doi:10.3389/fpubh.2025.1675040)
Supplement: Supplementary file 7 [file Table_7.docx]

**Supplementary Material 7.** Classification of Congenital Anomalies by Severity in Single Anomaly Cases: Criteria, Case Distribution, and Neonatal Mortality. State of São Paulo, 2009–2018**.**

| ICD-10 Category Description | **Criterion** | **Assigned Classification** | **Total Observed Cases** | **Neonatal Deaths (%)** |
| --- | --- | --- | --- | --- |
| **Q51**: Other congenital malformations of uterus and cervix | < 10 cases or neonatal mortality proportion <20% | 1 | 1 | 0 |
| **Q97**: Other sex chromosome abnormalities, male phenotype | < 10 cases or neonatal mortality proportion <20% | 1 | 7 | 0 |
| **Q85**: Phakomatoses, not elsewhere classified | < 10 cases or neonatal mortality proportion <20% | 1 | 7 | 0 |
| **Q14**: Other congenital malformations of eyeball | < 10 cases or neonatal mortality proportion <20% | 1 | 5 | 20 |
| **Q95**: Translocations and inversions of chromosomes | < 10 cases or neonatal mortality proportion <20% | 1 | 2 | 50 |
| **Q86**: Congenital malformation syndromes due to known exogenous causes | < 10 cases or neonatal mortality proportion <20% | 1 | 9 | 11 |
| **Q93**: Other deletions of parts of chromosomes | < 10 cases or neonatal mortality proportion <20% | 1 | 8 | 13 |
| **Q83**: Congenital malformations of breast | < 10 cases or neonatal mortality proportion <20% | 1 | 246 | 0 |
| **Q53**: Cryptorchidism | < 10 cases or neonatal mortality proportion <20% | 1 | 823 | 1 |
| **Q69**: Absence of finger(s) | < 10 cases or neonatal mortality proportion <20% | 1 | 6,967 | 1 |
| **Q65**: Congenital hip dislocation | < 10 cases or neonatal mortality proportion <20% | 1 | 233 | 1 |
| **Q54**: Hypospadias | < 10 cases or neonatal mortality proportion <20% | 1 | 1,962 | 1 |
| **Q71**: Longitudinal reduction defects of upper limb | < 10 cases or neonatal mortality proportion <20% | 1 | 506 | 1 |
| **Q27**: Other congenital malformations of veins | < 10 cases or neonatal mortality proportion <20% | 1 | 581 | 1 |
| **Q70**: Congenital radioulnar synostosis | < 10 cases or neonatal mortality proportion <20% | 1 | 706 | 1 |
| **Q82**: Other congenital malformations of skin | < 10 cases or neonatal mortality proportion <20% | 1 | 230 | 2 |
| **Q38**: Other congenital malformations of tongue, mouth, and pharynx | < 10 cases or neonatal mortality proportion <20% | 1 | 453 | 2 |
| **Q52**: Other congenital malformations of female genital organs | < 10 cases or neonatal mortality proportion <20% | 1 | 108 | 2 |
| **Q84**: Other congenital malformations of skin | < 10 cases or neonatal mortality proportion <20% | 1 | 93 | 2 |
| **Q17**: Other congenital malformations of ear | < 10 cases or neonatal mortality proportion <20% | 1 | 1,740 | 2 |
| **Q36**: Unilateral cleft lip | < 10 cases or neonatal mortality proportion <20% | 1 | 1,046 | 3 |
| **Q90**: Down syndrome | < 10 cases or neonatal mortality proportion <20% | 1 | 2,231 | 3 |
| **Q66**: Congenital deformities of feet | < 10 cases or neonatal mortality proportion <20% | 1 | 5,262 | 3 |
| **Q16**: Congenital hearing loss | < 10 cases or neonatal mortality proportion <20% | 1 | 217 | 3 |
| **Q68**: Other congenital malformations of the musculoskeletal and connective system | < 10 cases or neonatal mortality proportion <20% | 1 | 455 | 4 |
| **Q37**: Cleft lip and palate | < 10 cases or neonatal mortality proportion <20% | 1 | 1,290 | 4 |
| **Q35**: Cleft palate | < 10 cases or neonatal mortality proportion <20% | 1 | 852 | 4 |
| **Q72**: Longitudinal reduction defects of lower limb | < 10 cases or neonatal mortality proportion <20% | 1 | 166 | 4 |
| **Q15**: Other congenital malformations of eye | < 10 cases or neonatal mortality proportion <20% | 1 | 46 | 4 |
| **Q55**: Other congenital malformations of male genital organs | < 10 cases or neonatal mortality proportion <20% | 1 | 241 | 5 |
| **Q76**: Congenital malformations of spine and bony thorax | < 10 cases or neonatal mortality proportion <20% | 1 | 211 | 5 |
| **Q50**: Congenital malformations of ovaries, fallopian tubes, and broad ligaments | < 10 cases or neonatal mortality proportion <20% | 1 | 20 | 5 |
| **Q43**: Other congenital malformations of intestine | < 10 cases or neonatal mortality proportion <20% | 1 | 214 | 5 |
| **Q18**: Other congenital malformations of face and neck | < 10 cases or neonatal mortality proportion <20% | 1 | 432 | 5 |
| **Q21**: Congenital malformations of cardiac septa | < 10 cases or neonatal mortality proportion <20% | 1 | 1,187 | 5 |
| **Q05**: Spina bifida | < 10 cases or neonatal mortality proportion <20% | 1 | 1,120 | 5 |
| **Q62**: Other congenital malformations of urinary system | < 10 cases or neonatal mortality proportion <20% | 1 | 348 | 6 |
| **Q02**: Microcephaly | < 10 cases or neonatal mortality proportion <20% | 1 | 433 | 6 |
| **Q41**: Atresia of small intestine | < 10 cases or neonatal mortality proportion <20% | 1 | 108 | 7 |
| **Q12**: Congenital lens malformations | < 10 cases or neonatal mortality proportion <20% | 1 | 29 | 7 |
| **Q74**: Other congenital malformations of limbs | < 10 cases or neonatal mortality proportion <20% | 1 | 1,127 | 7 |
| **Q06**: Other congenital malformations of spinal cord | < 10 cases or neonatal mortality proportion <20% | 1 | 48 | 8 |
| **Q96**: Turner syndrome | < 10 cases or neonatal mortality proportion <20% | 1 | 47 | 9 |
| **Q73**: Other reduction defects of limbs | < 10 cases or neonatal mortality proportion <20% | 1 | 68 | 9 |
| **Q13**: Congenital malformations of anterior segment of eye | < 10 cases or neonatal mortality proportion <20% | 1 | 20 | 10 |
| **Q11**: Other congenital malformations of eye | < 10 cases or neonatal mortality proportion <20% | 1 | 30 | 10 |
| **Q64**: Other congenital malformations of urinary system | < 10 cases or neonatal mortality proportion <20% | 1 | 155 | 10 |
| **Q42**: Atresia of large intestine | < 10 cases or neonatal mortality proportion <20% | 1 | 358 | 10 |
| **Q30**: Congenital malformations of nose | < 10 cases or neonatal mortality proportion <20% | 1 | 94 | 11 |
| **Q56**: True hermaphroditism and male pseudohermaphroditism | < 10 cases or neonatal mortality proportion <20% | 1 | 278 | 11 |
| **Q81**: Epidermolysis bullosa | < 10 cases or neonatal mortality proportion <20% | 1 | 16 | 13 |
| **Q40**: Other congenital malformations of stomach | < 10 cases or neonatal mortality proportion <20% | 1 | 39 | 13 |
| **Q67**: Other skull and facial deformities | < 10 cases or neonatal mortality proportion <20% | 1 | 124 | 13 |
| **Q26**: Other congenital malformations of pulmonary veins | < 10 cases or neonatal mortality proportion <20% | 1 | 23 | 13 |
| **Q22**: Congenital malformations of pulmonary and tricuspid valves | < 10 cases or neonatal mortality proportion <20% | 1 | 146 | 13 |
| **Q07**: Other congenital malformations of nervous system | < 10 cases or neonatal mortality proportion <20% | 1 | 137 | 13 |
| **Q39**: Other congenital malformations of esophagus | < 10 cases or neonatal mortality proportion <20% | 1 | 337 | 14 |
| **Q75**: Other congenital malformations of skull and facial bones | < 10 cases or neonatal mortality proportion <20% | 1 | 338 | 14 |
| **Q98**: Klinefelter syndrome | < 10 cases or neonatal mortality proportion <20% | 1 | 13 | 15 |
| **Q45**: Other congenital malformations of the digestive system | < 10 cases or neonatal mortality proportion <20% | 1 | 36 | 17 |
| **Q03**: Congenital hydrocephalus | < 10 cases or neonatal mortality proportion <20% | 1 | 839 | 17 |
| **Q10**: Other congenital malformations of eyelid, lacrimal apparatus, and orbit | < 10 cases or neonatal mortality proportion <20% | 1 | 56 | 18 |
| **Q20**: Congenital malformations of cardiac chambers and connections | < 10 cases or neonatal mortality proportion <20% | 1 | 336 | 19 |
| **Q89**: Other congenital malformations, not elsewhere classified | < 10 cases or neonatal mortality proportion <20% | 1 | 3,463 | 19 |
| **Q04**: Other congenital malformations of the brain | neonatal mortality proportion ≥20% and <40% | 2 | 371 | 20 |
| **Q99**: Other chromosome abnormalities | neonatal mortality proportion ≥20% and <40% | 2 | 89 | 20 |
| **Q25**: Other congenital malformations of great arteries | neonatal mortality proportion ≥20% and <40% | 2 | 518 | 21 |
| **Q87**: Other specified syndromes with multiple congenital malformations | neonatal mortality proportion ≥20% and <40% | 2 | 351 | 21 |
| **Q63**: Other congenital malformations of kidney | neonatal mortality proportion ≥20% and <40% | 2 | 123 | 22 |
| **Q44**: Other congenital malformations of bile ducts | neonatal mortality proportion ≥20% and <40% | 2 | 35 | 23 |
| **Q01**: Encephalocele | neonatal mortality proportion ≥20% and <40% | 2 | 226 | 25 |
| **Q79**: Other congenital malformations of the musculoskeletal system | neonatal mortality proportion ≥20% and <40% | 2 | 2,304 | 27 |
| **Q28**: Other congenital malformations of the circulatory system | neonatal mortality proportion ≥20% and <40% | 2 | 38 | 29 |
| **Q31**: Congenital malformations of trachea and bronchi | neonatal mortality proportion ≥20% and <40% | 2 | 10 | 30 |
| **Q77**: Osteochondrodysplasia with dwarfism | neonatal mortality proportion ≥20% and <40% | 2 | 202 | 31 |
| **Q80**: Congenital ichthyosis | neonatal mortality proportion ≥20% and <40% | 2 | 32 | 31 |
| **Q33**: Pulmonary hypoplasia or aplasia | neonatal mortality proportion ≥20% and <40% | 2 | 205 | 32 |
| **Q23**: Other congenital malformations of cardiac valves | neonatal mortality proportion ≥20% and <40% | 2 | 257 | 32 |
| **Q24**: Other congenital malformations of heart | neonatal mortality proportion ≥20% and <40% | 2 | 1,230 | 33 |
| **Q34**: Other congenital malformations of the respiratory system | neonatal mortality proportion ≥20% and <40% | 2 | 12 | 33 |
| **Q92**: Other trisomies and monosomies, not elsewhere classified | neonatal mortality proportion ≥20% and <40% | 2 | 22 | 36 |
| **Q32**: Other congenital malformations of larynx | neonatal mortality proportion ≥20% and <40% | 2 | 26 | 39 |
| **Q78**: Other osteochondrodysplasias | neonatal mortality proportion ≥40% | 3 | 76 | 42 |
| **Q61**: Congenital cystic kidney disease | neonatal mortality proportion ≥40% | 3 | 170 | 42 |
| **Q91**: Edwards syndrome | neonatal mortality proportion ≥40% | 3 | 238 | 48 |
| **Q60**: Renal agenesis | neonatal mortality proportion ≥40% | 3 | 190 | 57 |
| **Q00**: Anencephaly | neonatal mortality proportion ≥40% | 3 | 654 | 92 |
