# Supplementary material for: Development and validation of an epidemiological risk score for neonatal death in a middle-income country
Source: Front Public Health. 2025 Nov 19;13:1675040. doi: 10.3389/fpubh.2025.1675040 (PMC12672502; doi:10.3389/fpubh.2025.1675040)
Supplement: Supplementary file 8 [file Table_8.docx]

**Supplementary Material 8.** Descriptive statistics of municipal indicators and their correlation with neonatal mortality rate. State of São Paulo, 2009–2018.

|  |  | **Measures of Central Tendency and Variability** | | | | | | | | |  | **Correlation with NMR** | |
| --- | --- | --- | --- | --- | --- | --- | --- | --- | --- | --- | --- | --- | --- |
| **Thematic Block and Indicator** | **mean** | **SD** | **p25** | **p50** | **p75** | **p10** | **p90** | **min** | **max** | **N** |  | **r** | **p** |
| **Access to the Health System** |  |  |  |  |  |  |  |  |  |  |  |  |  |
| Estimated population coverage by primary health care teams (%) | 74.7 | 29.1 | 53.2 | 86.3 | 100 | 31.6 | 100 | 0 | 100 | 6,450 |  | 0.023 | 0.063 |
| Percentage of the population covered by community-based primary care teams (%) | 58.8 | 38.0 | 23.7 | 66.2 | 100 | 0 | 100 | 0 | 100 | 6,450 |  | 0.019 | 0.134 |
| Percentage of the population covered by private health insurance plans (%)* | 21.2 | 15.0 | 9.2 | 18.7 | 30.2 | 5.2 | 41.2 | 0.3 | 248.5 | 6,450 |  | -0.030 | 0.015 |
| **Demographic** |  |  |  |  |  |  |  |  |  |  |  |  |  |
| Population density (inhabitants per km²) | 322.0 | 1,265.9 | 20.6 | 40.2 | 117.1 | 13.2 | 409 | 3.7 | 13,945.2 | 6,450 |  | - | 0.975 |
| Health System Management |  |  |  |  |  |  |  |  |  |  |  |  |  |
| Percentage of municipal funds allocated to public health services (%) | 24.7 | 4.7 | 21.2 | 24.4 | 27.9 | 18.8 | 31.1 | 13.5 | 50.1 | 6,445 |  | -0.022 | 0.072 |
| **Physical Resources** |  |  |  |  |  |  |  |  |  |  |  |  |  |
| MRI machines (units per 100,000 inhabitants) | 0.2 | 0.6 | 0 | 0 | 0 | 0 | 0.7 | 0 | 6.16 | 6,450 |  | - | 0.850 |
| MRI machines available in the public health system (units per 100,000 inhabitants) | 0.1 | 0.4 | 0 | 0 | 0 | 0 | 0 | 0 | 4.15 | 6,450 |  | - | 0.840 |
| Ultrasound machines (units per 100,000 inhabitants) | 11.0 | 10.9 | 0 | 9.3 | 16.9 | 0 | 25.4 | 0 | 80.4 | 6,450 |  | -0.023 | 0.068 |
| Ultrasound machines available in the public health system (units per 100,000 inhabitants) | 7.3 | 8.8 | 0 | 5.3 | 10.8 | 0 | 17.6 | 0 | 80.4 | 6,450 |  | -0.034 | 0.007 |
| Hospital beds available in the public health system (beds per 1,000 inhabitants) | 0.7 | 0.9 | 0 | 0.4 | 1.2 | 0 | 2 | 0 | 7.7 | 6,450 |  | - | 0.272 |
| Total hospital beds (beds per 1,000 inhabitants) | 1.1 | 1.4 | 0 | 0.6 | 1.9 | 0 | 3 | 0 | 10.1 | 6,450 |  | - | 0.895 |
| CT scanners available in the public sector (units per 100,000 inhabitants) | 0.4 | 1.0 | 0 | 0 | 0 | 0 | 1.6 | 0 | 11.5 | 6,450 |  | - | 0.885 |
| Total CT scanners (units per 100,000 inhabitants) | 0.6 | 1.5 | 0 | 0 | 0 | 0 | 2.6 | 0 | 14.4 | 6,450 |  | - | 0.909 |
| **Human Resources** |  |  |  |  |  |  |  |  |  |  |  |  |  |
| Nurses available in the public sector (per 100,000 inhabitants) | 76.8 | 44.6 | 47.7 | 67 | 94.9 | 33.7 | 128.7 | 0 | 458.5 | 6,450 |  | -0.028 | 0.025 |
| Total nurses (per 100,000 inhabitants) | 79.6 | 46.0 | 49.5 | 69.9 | 98.3 | 34.5 | 134.3 | 0 | 458.5 | 6,450 |  | -0.029 | 0.020 |
| Physicians available in the public sector (per 1,000 inhabitants) | 0.9 | 0.8 | 0.4 | 0.7 | 1.2 | 0.2 | 1.7 | 0 | 15.6 | 6,450 |  | -0.017 | 0.168 |
| Total physicians (per 1,000 inhabitants) | 1.0 | 1.0 | 0.4 | 0.8 | 1.4 | 0.2 | 2 | 0 | 15.7 | 6,450 |  | -0.018 | 0.155 |
| Intensive care physicians available in the public sector (per 100,000 inhabitants) | 0.2 | 0.9 | 0 | 0 | 0 | 0 | 0 | 0 | 16.9 | 6,450 |  | - | 0.768 |
| Total intensive care physicians (per 100,000 inhabitants) | 0.2 | 1.0 | 0 | 0 | 0 | 0 | 0 | 0 | 16.9 | 6,450 |  | - | 0.902 |
| OB-GYNs, mastologists, and breast surgeons available in the public sector (per 100,000 inhabitants) | 3.1 | 6.5 | 0 | 0 | 3.9 | 0 | 10.7 | 0 | 121.4 | 6,450 |  | - | 0.312 |
| Total OB-GYNs, mastologists, and breast surgeons (per 100,000 inhabitants) | 4.0 | 7.6 | 0 | 0 | 5.4 | 0 | 13.7 | 0 | 121.4 | 6,450 |  | - | 0.319 |
| Pediatricians, adolescent medicine specialists, and neonatologists available in the public sector (per 100,000 inhabitants) | 8.9 | 11.8 | 0 | 5.2 | 14 | 0 | 23.7 | 0 | 120.5 | 6,450 |  | -0.027 | 0.031 |
| Total pediatricians, adolescent medicine specialists, and neonatologists (per 100,000 inhabitants) | 10.2 | 13.3 | 0 | 6.1 | 16.4 | 0 | 27.2 | 0 | 125 | 6,450 |  | -0.028 | 0.024 |

(*) Values greater than 100% indicate more health insurance affiliations than the total population in that area/year (<https://www.ans.gov.br/anstabnet/notas_taxa_cobertura.htm>)

• **CT**: Computed Tomography

• **OB-GYN**: Obstetrician-Gynecologist

• *Public sector*: Refers to services and resources available through the Brazilian public health system (“Sistema Único de Saúde” – SUS).

• *Community-based primary care teams*: Multidisciplinary teams providing primary health care at the community level, including physicians, nurses, and community health workers, in line with the structure of the “Family Health Strategy”.
