# Supplementary material for: Development and validation of an epidemiological risk score for neonatal death in a middle-income country
Source: Front Public Health. 2025 Nov 19;13:1675040. doi: 10.3389/fpubh.2025.1675040 (PMC12672502; doi:10.3389/fpubh.2025.1675040)
Supplement: Supplementary file 9 [file Table_9.docx]

**Supplementary Material 9.** Correlation matrix of standardized municipal indicators associated with neonatal mortality rate. State of São Paulo, 2009–2018.

|  |  | **(1)** | **(2)** | **(3)** | **(4)** | **(5)** | **(6)** | **(7)** | **(8)** | **(9)** | **(10)** | **(11)** |
| --- | --- | --- | --- | --- | --- | --- | --- | --- | --- | --- | --- | --- |
| Primary Health Care | **(1)** | 1.00 |  |  |  |  |  |  |  |  |  |  |
| Community-Based Primary Care Teams | **(2)** | 0.83 | 1.00 |  |  |  |  |  |  |  |  |  |
| Private Health Insurance Coverage | **(3)** | -0.28 | -0.35 | 1.00 |  |  |  |  |  |  |  |  |
| Municipal Funds Allocated to Health | **(4)** | -0.05 | -0.11 | 0.09 | 1.00 |  |  |  |  |  |  |  |
| Ultrasound Machines | **(5)** | 0.08 | 0.06 | 0.01 | 0.05 | 1.00 |  |  |  |  |  |  |
| Ultrasound Machines (Public Health System) | **(6)** | -0.07 | -0.09 | 0.19 | 0.11 | 0.82 | 1.00 |  |  |  |  |  |
| Nurses (Public Health System) | **(7)** | 0.28 | 0.30 | -0.11 | 0.04 | 0.13 | 0.16 | 1.00 |  |  |  |  |
| Total Nurses | **(8)** | 0.24 | 0.26 | -0.04 | 0.05 | 0.12 | 0.19 | 0.98 | 1.00 |  |  |  |
| Physicians (Public Health System) | **(9)** | -0.07 | -0.12 | 0.23 | 0.09 | 0.14 | 0.29 | 0.41 | 0.44 | 1.00 |  |  |
| Total Physicians | **(10)** | -0.13 | -0.17 | 0.34 | 0.11 | 0.11 | 0.33 | 0.37 | 0.43 | 0.97 | 1.00 |  |
| Pediatricians (Public Health System) | **(11)** | -0.09 | -0.15 | 0.23 | 0.12 | 0.06 | 0.16 | 0.22 | 0.25 | 0.52 | 0.54 | 1.00 |
| Total Pediatricians | **(12)** | -0.13 | -0.18 | 0.31 | 0.13 | 0.04 | 0.19 | 0.21 | 0.26 | 0.54 | 0.60 | 0.97 |

Note: All correlations >= 0.40 are statistically significant (p < 0.001).

Parte superior do formulário

Parte inferior do formulário
