# Supplementary material for: Development and validation of an epidemiological risk score for neonatal death in a middle-income country
Source: Front Public Health. 2025 Nov 19;13:1675040. doi: 10.3389/fpubh.2025.1675040 (PMC12672502; doi:10.3389/fpubh.2025.1675040)
Supplement: Supplementary file 10 [file Table_10.docx]

Parte superior do formulário

Parte inferior do formulário

### Supplementary Material 10. Correlation groups of standardized municipal indicators associated with neonatal mortality rate. State of São Paulo, 2009–2018.

| **Correlation Group** | **Indicator Pairs and Correlation Coefficients** |
| --- | --- |
| Group 1 | Primary Health Care and Community-Based Primary Care Teams: 0.83 |
| Group 2 | Nurses (Public Health System) and Total Nurses: 0.98 |
| Group 3 | Ultrasound Machines (Public Health System) and Total Ultrasound Machines: 0.82 |
| Group 4 | Physicians (Public Health System) and Total Physicians: 0.97 |
|  | Pediatricians (Public Health System) and Total Pediatricians: 0.97 |
|  | Total Pediatricians and Total Physicians: 0.60 |
|  | Pediatricians (Public Health System) and Total Physicians: 0.54 |
|  | Total Pediatricians and Physicians (Public Health System): 0.54 |
|  | Pediatricians (Public Health System) and Physicians (Public Health System): 0.52 |
