# Supplementary material for: Development and validation of an epidemiological risk score for neonatal death in a middle-income country
Source: Front Public Health. 2025 Nov 19;13:1675040. doi: 10.3389/fpubh.2025.1675040 (PMC12672502; doi:10.3389/fpubh.2025.1675040)
Supplement: Supplementary file 11 [file Table_11.docx]

Parte superior do formulário

Parte inferior do formulário

### Supplementary Material 11. ****Absolute and relative f****requencies of dichotomous variables for independent municipal indicators associated with neonatal mortality rate. State of São Paulo, 2009–2018.

| **Indicator** | **Category** | **N** | **%** |
| --- | --- | --- | --- |
| Percentage of the population covered by private health insurance plans (%) | <21.2 | 3,694 | 57.3 |
|  | >=21.2 | 2,756 | 42.7 |
| Number of ultrasound machines available in the public health system (per 100,000 inhabitants) | <11.0 | 3,976 | 61.6 |
|  | >=11.0 | 2,474 | 38.4 |
| Number of nurses available in the public health system (per 100,000 inhabitants) | <76.8 | 3,902 | 60.5 |
|  | >=76.8 | 2,548 | 39.5 |
