# Supplementary material for: Development and validation of an epidemiological risk score for neonatal death in a middle-income country
Source: Front Public Health. 2025 Nov 19;13:1675040. doi: 10.3389/fpubh.2025.1675040 (PMC12672502; doi:10.3389/fpubh.2025.1675040)
Supplement: Supplementary file 12 [file Table_12.docx]

**Parte superior do formulário**

**Parte inferior do formulário**

### Supplementary Material 12. Nurses available in the public health system per 100,000 inhabitants. State of São Paulo, 2008–2018.

| Municipality | Code | 2008 | 2009 | 2010 | 2011 | 2012 | 2013 | 2014 | 2015 | 2016 | 2017 | 2018 |
| --- | --- | --- | --- | --- | --- | --- | --- | --- | --- | --- | --- | --- |
| Adamantina-SP | 350010 | 86.8 | 89.6 | 92.4 | 121.1 | 132.4 | 135.1 | 140.7 | 137.6 | 148.9 | 154.4 | 159.9 |
| Adolfo-SP | 350020 | 27.3 | 27.4 | 54.9 | 55 | 55.1 | 82.9 | 83.1 | 83.4 | 83.6 | 111.7 | 112 |
| Aguaí-SP | 350030 | 24.9 | 27.7 | 21.2 | 18 | 38.6 | 38.1 | 31.9 | 37.3 | 45.4 | 39.3 | 64 |
| Águas da Prata-SP | 350040 | 26.1 | 25.9 | 38.6 | 38.4 | 38.1 | 37.9 | 37.7 | 62.5 | 62.1 | 61.8 | 61.4 |
| Águas de Lindóia-SP | 350050 | 63 | 51.2 | 45.2 | 44.9 | 61.4 | 72.1 | 82.6 | 82.1 | 76.2 | 64.9 | 86 |
| Águas de Santa Bárbara-SP | 350055 | 70.7 | 35.1 | 69.7 | 69.2 | 51.6 | 51.2 | 50.9 | 84.3 | 150.8 | 66.6 | 149 |
| Águas de São Pedro-SP | 350060 | 76.7 | 74.3 | 72.1 | 70.2 | 102.5 | 199.7 | 162.3 | 189.9 | 92.7 | 60.5 | 118.3 |
| Agudos-SP | 350070 | 31.5 | 34.1 | 48 | 56.2 | 67 | 61.1 | 77.3 | 68.6 | 101 | 95 | 94.5 |
| Alambari-SP | 350075 | 21 | 61.4 | 79.9 | 78.1 | 114.5 | 130.8 | 109.7 | 107.4 | 105.3 | 120.5 | 118.3 |
| Alfredo Marcondes-SP | 350080 | 76 | 75.6 | 50.1 | 49.9 | 74.5 | 74.1 | 73.7 | 73.4 | 73 | 72.7 | 72.3 |
| Altair-SP | 350090 | 77.9 | 77.3 | 76.7 | 76.2 | 151.2 | 150.2 | 149.1 | 148 | 147 | 146.1 | 120.9 |
| Altinópolis-SP | 350100 | 62.7 | 81.4 | 112.5 | 106.1 | 93.5 | 93.4 | 105.7 | 136.6 | 148.8 | 142.5 | 142.3 |
| Alto Alegre-SP | 350110 | 70.9 | 71.1 | 47.6 | 47.7 | 119.6 | 95.9 | 168.4 | 168.9 | 169.4 | 169.8 | 194.6 |
| Alumínio-SP | 350115 | 41.4 | 40.9 | 52.1 | 45.9 | 51.2 | 50.8 | 50.3 | 49.9 | 49.5 | 54.5 | 70.3 |
| Álvares Florence-SP | 350120 | 73.7 | 49.6 | 50.1 | 50.5 | 76.5 | 25.7 | 51.9 | 78.6 | 79.4 | 106.8 | 80.8 |
| Álvares Machado-SP | 350130 | 58.6 | 66.7 | 53.9 | 62 | 65.9 | 65.6 | 65.4 | 65.1 | 77.1 | 76.8 | 80.5 |
| Álvaro de Carvalho-SP | 350140 | 85.9 | 84.9 | 62.9 | 83 | 82.1 | 81.3 | 100.5 | 139.2 | 137.8 | 97.5 | 115.9 |
| Alvinlândia-SP | 350150 | 32.9 | 32.7 | 32.5 | 64.7 | 64.4 | 64 | 63.7 | 63.3 | 63 | 62.7 | 62.4 |
| Americana-SP | 350160 | 33.8 | 37.1 | 46.8 | 50.3 | 53.8 | 63 | 71.9 | 64.1 | 57.3 | 49.5 | 46 |
| Américo Brasiliense-SP | 350170 | 38.1 | 80.7 | 116 | 197.6 | 208.1 | 250.7 | 244.2 | 279.5 | 247.1 | 258.8 | 240.2 |
| Américo de Campos-SP | 350180 | 85.9 | 85.7 | 51.3 | 51.2 | 51.1 | 67.9 | 84.7 | 84.5 | 84.3 | 101 | 100.7 |
| Amparo-SP | 350190 | 73.9 | 82.2 | 97.8 | 101.4 | 100.7 | 108.6 | 117.8 | 111.2 | 113.2 | 125 | 139.5 |
| Analândia-SP | 350200 | 23.5 | 23.1 | 22.7 | 22.4 | 44.1 | 43.4 | 107.1 | 126.6 | 145.7 | 102.7 | 121.6 |
| Andradina-SP | 350210 | 49.5 | 58.2 | 75.8 | 95.1 | 103.8 | 96.7 | 100.1 | 101.8 | 108.7 | 110.4 | 122.6 |
| Angatuba-SP | 350220 | 18 | 26.7 | 30.7 | 34.7 | 47.2 | 50.8 | 46.1 | 37.2 | 45 | 52.6 | 56.1 |
| Anhembi-SP | 350230 | 71.9 | 88 | 86.3 | 101.7 | 99.9 | 98.2 | 112.6 | 94.9 | 93.4 | 76.6 | 60.4 |
| Anhumas-SP | 350240 | 53.1 | 52.7 | 52.2 | 51.8 | 51.3 | 50.9 | 50.5 | 75.1 | 74.6 | 74 | 73.4 |
| Aparecida-SP | 350250 | 44.7 | 55.8 | 58.5 | 52.9 | 69.5 | 77.8 | 97.2 | 91.6 | 108.1 | 94.2 | 99.6 |
| Aparecida d'Oeste-SP | 350260 | 64.5 | 65.1 | 109.6 | 88.5 | 134 | 112.7 | 159.2 | 160.8 | 162.3 | 187.2 | 188.9 |
| Apiaí-SP | 350270 | 53.5 | 53.8 | 62 | 85.7 | 94.1 | 102.6 | 119.2 | 104 | 100.7 | 113.4 | 142.7 |
| Araçariguama-SP | 350275 | 55.2 | 59.1 | 45.7 | 49.8 | 48.4 | 73.1 | 71 | 78.9 | 72.1 | 112.5 | 109.8 |
| Araçatuba-SP | 350280 | 56.7 | 61.7 | 71.5 | 82.2 | 102.3 | 98 | 108.9 | 123.8 | 126.1 | 124.8 | 124.6 |
| Araçoiaba da Serra-SP | 350290 | 22.7 | 25.7 | 25 | 20.9 | 20.4 | 33.2 | 52 | 85.8 | 80.8 | 82.2 | 71.6 |
| Aramina-SP | 350300 | 38.5 | 19.1 | 56.8 | 75.2 | 74.7 | 92.7 | 73.6 | 73.1 | 108.9 | 162.2 | 125.3 |
| Arandu-SP | 350310 | 47.9 | 95.7 | 79.7 | 31.8 | 79.4 | 79.3 | 95 | 110.7 | 110.6 | 157.7 | 157.5 |
| Arapeí-SP | 350315 | 116.4 | 77.9 | 117.4 | 78.6 | 157.7 | 197.9 | 198.6 | 159.6 | 200.2 | 201 | 201.8 |
| Araraquara-SP | 350320 | 49.9 | 50.7 | 51 | 61 | 78.6 | 80.8 | 89.3 | 92.7 | 100.9 | 99.8 | 115.1 |
| Araras-SP | 350330 | 53 | 60.7 | 57.5 | 56.8 | 81 | 84.1 | 93.4 | 106.3 | 102.9 | 104.1 | 109.8 |
| Arco-SPÍris-SP | 350335 | 99.1 | 100.3 | 101.4 | 102.4 | 103.5 | 104.7 | 105.8 | 107 | 108.1 | 109.3 | 110.4 |
| Arealva-SP | 350340 | 37.9 | 62.7 | 62.2 | 61.8 | 61.3 | 73 | 108.8 | 120 | 119.1 | 130.2 | 141.1 |
| Areias-SP | 350350 | 26.6 | 26.5 | 26.4 | 26.3 | 105 | 104.7 | 78.3 | 104.1 | 129.7 | 155.2 | 154.8 |
| Areiópolis-SP | 350360 | 18.6 | 18.5 | 36.9 | 55.2 | 45.8 | 27.4 | 27.3 | 45.4 | 63.4 | 54.2 | 72.1 |
| Ariranha-SP | 350370 | 46.8 | 46.2 | 45.7 | 45.1 | 33.5 | 44.1 | 54.5 | 64.7 | 64 | 52.8 | 62.7 |
| Artur Nogueira-SP | 350380 | 39.5 | 52.1 | 39.7 | 49.7 | 50.7 | 43.4 | 52.6 | 69.3 | 71.9 | 70.5 | 71.1 |
| Arujá-SP | 350390 | 27.2 | 17.3 | 35.2 | 29.4 | 57.7 | 57.9 | 62.9 | 66.5 | 63 | 59.7 | 59.9 |
| Aspásia-SP | 350395 | 107.4 | 107.6 | 107.9 | 108.1 | 162.4 | 271.3 | 271.9 | 163.4 | 163.8 | 164 | 219.2 |
| Assis-SP | 350400 | 103.3 | 102.4 | 116.9 | 126.1 | 143.3 | 148.2 | 161 | 184.4 | 176.1 | 172 | 166.9 |
| Atibaia-SP | 350410 | 20.5 | 30.4 | 30.8 | 33.5 | 46.7 | 47.7 | 47.9 | 49.6 | 62 | 58.6 | 58.7 |
| Auriflama-SP | 350420 | 34.7 | 55.2 | 54.9 | 82 | 95.2 | 101.5 | 107.8 | 120.7 | 113.4 | 119.6 | 119 |
| Avaí-SP | 350430 | 20 | 39.6 | 39.3 | 78.1 | 77.6 | 77 | 38.3 | 57 | 56.6 | 75 | 55.9 |
| Avanhandava-SP | 350440 | 36.1 | 35.3 | 69 | 50.7 | 49.8 | 48.8 | 79.8 | 78.4 | 53.9 | 60.6 | 44.7 |
| Avaré-SP | 350450 | 46.6 | 45.1 | 42.3 | 51.4 | 59.1 | 59.8 | 67.4 | 78.2 | 87.8 | 88.3 | 98.8 |
| Bady Bassitt-SP | 350460 | 41.8 | 47.7 | 40.1 | 52.4 | 64.3 | 63.2 | 62.1 | 73.1 | 83.9 | 94.3 | 98.6 |
| Balbinos-SP | 350470 | 30.1 | 28.1 | 26.4 | 74.7 | 47.2 | 44.9 | 128.4 | 102.1 | 97.8 | 112.7 | 126.5 |
| Bálsamo-SP | 350480 | 12.2 | 12.1 | 23.9 | 23.7 | 35.2 | 34.9 | 34.6 | 22.8 | 33.9 | 78.5 | 66.7 |
| Bananal-SP | 350490 | 38.6 | 57.6 | 76.3 | 66.5 | 66.1 | 47 | 56.1 | 65.2 | 64.8 | 64.5 | 64.2 |
| Barão de Antonina-SP | 350500 | 32 | 63.3 | 62.6 | 62 | 61.4 | 60.8 | 60.3 | 59.7 | 59.2 | 58.6 | 58.1 |
| Barbosa-SP | 350510 | 60.6 | 59.9 | 59.2 | 58.5 | 43.4 | 57.3 | 99.3 | 84.2 | 69.5 | 82.6 | 81.8 |
| Bariri-SP | 350520 | 28.4 | 40.6 | 43.2 | 67.3 | 63.6 | 77.9 | 86.1 | 70.5 | 75.7 | 77.9 | 68.6 |
| Barra Bonita-SP | 350530 | 49.8 | 52.6 | 55.4 | 58.1 | 58.1 | 58.1 | 63.7 | 60.9 | 55.4 | 55.4 | 77.5 |
| Barra do Chapéu-SP | 350535 | 37.8 | 75 | 74.4 | 55.4 | 55 | 54.6 | 54.2 | 53.8 | 71.3 | 106.2 | 123.1 |
| Barra do Turvo-SP | 350540 | 62.6 | 100.6 | 75.7 | 88.7 | 76.3 | 102.1 | 141 | 154.4 | 155 | 181.5 | 182.1 |
| Barretos-SP | 350550 | 110.5 | 161.3 | 177.5 | 193.6 | 227.4 | 217.3 | 290.3 | 324.4 | 400.7 | 359.1 | 367.6 |
| Barrinha-SP | 350560 | 35.3 | 34.8 | 34.2 | 33.8 | 33.3 | 29.6 | 45.4 | 44.8 | 50.5 | 53 | 55.5 |
| Barueri-SP | 350570 | 32.1 | 87.9 | 107.8 | 96 | 104.7 | 109.7 | 125.4 | 126.2 | 134.1 | 138.2 | 162.5 |
| Bastos-SP | 350580 | 76.3 | 81.1 | 81.1 | 42.9 | 47.7 | 47.7 | 52.5 | 57.3 | 66.8 | 90.7 | 124.1 |
| Batatais-SP | 350590 | 54.6 | 62.8 | 67.4 | 53.1 | 62.8 | 70.6 | 75 | 81 | 83.6 | 94.3 | 88.7 |
| Bauru-SP | 350600 | 90.6 | 99.8 | 108.6 | 115.1 | 119.8 | 124.7 | 146.3 | 149 | 146.6 | 148.8 | 154.4 |
| Bebedouro-SP | 350610 | 39.1 | 45.5 | 59.8 | 58.5 | 66.2 | 66.1 | 70 | 79 | 80.2 | 85.3 | 103.3 |
| Bento de Abreu-SP | 350620 | 74.6 | 73.7 | 109.4 | 108.4 | 107.3 | 106.3 | 70.2 | 69.5 | 68.9 | 102.4 | 101.5 |
| Bernardino de Campos-SP | 350630 | 45.4 | 72.5 | 54.3 | 72.4 | 72.3 | 90.2 | 72.1 | 63 | 63 | 44.9 | 62.9 |
| Bertioga-SP | 350635 | 37.5 | 36.1 | 30.7 | 67.4 | 76.8 | 80 | 79.5 | 42.1 | 64.8 | 61.5 | 61.6 |
| Bilac-SP | 350640 | 71.2 | 70.2 | 83 | 95.7 | 81 | 93.3 | 92.2 | 104.1 | 102.9 | 139.9 | 151 |
| Birigui-SP | 350650 | 48.9 | 60 | 64.6 | 61.2 | 61.3 | 64 | 64.1 | 65.9 | 65.1 | 77.7 | 81.7 |
| Biritiba-SPMirim-SP | 350660 | 31.6 | 38.1 | 41 | 37.1 | 46.6 | 46 | 48.7 | 54.5 | 44.4 | 47 | 49.6 |
| Boa Esperança do Sul-SP | 350670 | 43.6 | 36 | 57.2 | 49.7 | 63.4 | 62.9 | 83.3 | 89.5 | 82.1 | 74.7 | 74.2 |
| Bocaina-SP | 350680 | 55.4 | 45.5 | 44.9 | 62.1 | 61.4 | 60.7 | 51.4 | 50.8 | 41.9 | 49.7 | 57.4 |
| Bofete-SP | 350690 | 31.9 | 41.5 | 40.6 | 39.7 | 38.9 | 38.1 | 28 | 54.9 | 53.9 | 52.9 | 34.7 |
| Boituva-SP | 350700 | 30 | 24.9 | 22.2 | 35.4 | 38.4 | 43.1 | 51.2 | 66 | 68 | 61.5 | 66.9 |
| Bom Jesus dos Perdões-SP | 350710 | 31.7 | 20.4 | 34.6 | 43.3 | 46.7 | 50 | 48.7 | 56.1 | 42.1 | 45.2 | 44.2 |
| Bom Sucesso de Itararé-SP | 350715 | 27.9 | 27.6 | 54.6 | 27.1 | 26.8 | 26.6 | 26.4 | 26.1 | 51.8 | 51.4 | 51 |
| Borá-SP | 350720 | 243.3 | 242.7 | 363.6 | 242.1 | 362.3 | 361.9 | 361.4 | 240.4 | 240.1 | 239.8 | 239.2 |
| Boracéia-SP | 350730 | 46.9 | 46.3 | 68.6 | 67.8 | 67 | 66.3 | 87.4 | 64.8 | 64.1 | 63.5 | 83.8 |
| Borborema-SP | 350740 | 54.7 | 54.2 | 67.1 | 73.2 | 79.2 | 85.1 | 90.8 | 96.5 | 89.3 | 82.3 | 81.6 |
| Borebi-SP | 350745 | 43.9 | 43.2 | 85.1 | 125.8 | 41.3 | 81.5 | 120.6 | 39.7 | 78.2 | 38.6 | 38.2 |
| Botucatu-SP | 350750 | 206.9 | 209.2 | 212.2 | 191.2 | 202 | 204.5 | 261.3 | 269.3 | 266.6 | 275.3 | 294.2 |
| Bragança Paulista-SP | 350760 | 56.2 | 62.8 | 63.2 | 72.8 | 73.8 | 74.7 | 84.4 | 94.5 | 95.2 | 92.2 | 112.7 |
| Braúna-SP | 350770 | 79.8 | 59 | 58.3 | 57.6 | 75.9 | 93.8 | 92.7 | 91.7 | 90.7 | 71.8 | 88.8 |
| Brejo Alegre-SP | 350775 | 38.7 | 38.3 | 37.9 | 37.5 | 37.2 | 36.8 | 36.5 | 72.3 | 71.6 | 71 | 70.4 |
| Brodowski-SP | 350780 | 28.8 | 37.7 | 41.6 | 50 | 40.2 | 87.9 | 95.1 | 93.6 | 83.7 | 90.8 | 97.6 |
| Brotas-SP | 350790 | 46.4 | 45.8 | 49.7 | 49.2 | 57.4 | 69.9 | 69.1 | 55.5 | 67.6 | 66.9 | 66.2 |
| Buri-SP | 350800 | 42.5 | 26.4 | 42 | 47.1 | 41.6 | 46.6 | 51.5 | 56.4 | 51 | 45.7 | 55.6 |
| Buritama-SP | 350810 | 71.1 | 95.9 | 88.6 | 125.3 | 124.2 | 110.7 | 115.8 | 120.7 | 131.6 | 124.6 | 123.5 |
| Buritizal-SP | 350820 | 49.1 | 72.9 | 72.2 | 47.7 | 70.9 | 93.8 | 69.7 | 69.1 | 68.5 | 68 | 45 |
| Cabrália Paulista-SP | 350830 | 44.2 | 22.2 | 67 | 67.4 | 67.8 | 68.1 | 91.3 | 68.9 | 46.2 | 69.6 | 70 |
| Cabreúva-SP | 350840 | 36.7 | 33.5 | 30.5 | 32.2 | 29.4 | 31.1 | 28.4 | 36.4 | 54.8 | 60.2 | 79.6 |
| Caçapava-SP | 350850 | 44.7 | 37.2 | 43.7 | 36.5 | 51.9 | 58.2 | 59.9 | 68.1 | 65.3 | 64.7 | 63.1 |
| Cachoeira Paulista-SP | 350860 | 49.6 | 49.1 | 58.4 | 54.6 | 57.3 | 66.3 | 65.7 | 74.4 | 61.5 | 88.4 | 84.7 |
| Caconde-SP | 350870 | 21.4 | 32 | 32 | 47.9 | 53.2 | 53.1 | 74.2 | 68.8 | 68.7 | 68.6 | 68.6 |
| Cafelândia-SP | 350880 | 47.5 | 53.2 | 52.9 | 52.6 | 58.2 | 63.7 | 63.4 | 74.5 | 97 | 107.9 | 124.4 |
| Caiabu-SP | 350890 | 71.9 | 71.9 | 71.9 | 71.8 | 71.8 | 71.8 | 71.7 | 71.7 | 71.7 | 71.6 | 71.6 |
| Caieiras-SP | 350900 | 30.4 | 29.8 | 33.8 | 32.2 | 26.2 | 36.5 | 33.9 | 61.5 | 66.7 | 80 | 87.9 |
| Caiuá-SP | 350910 | 100.2 | 98.4 | 96.8 | 95.3 | 93.9 | 55.5 | 54.7 | 71.8 | 53.1 | 69.9 | 69 |
| Cajamar-SP | 350920 | 36.5 | 40.4 | 38 | 41.8 | 29.3 | 36 | 52.3 | 58.3 | 77.8 | 83.3 | 63.5 |
| Cajati-SP | 350925 | 34.2 | 34.3 | 30.9 | 44.8 | 51.8 | 62.3 | 72.8 | 76.4 | 62.7 | 73.3 | 73.4 |
| Cajobi-SP | 350930 | 60.7 | 60.3 | 49.9 | 49.6 | 39.5 | 39.2 | 39 | 38.8 | 67.5 | 86.3 | 95.4 |
| Cajuru-SP | 350940 | 29.9 | 46.4 | 66.8 | 74.4 | 94 | 93.1 | 92.2 | 91.2 | 90.3 | 97.3 | 92.5 |
| Campina do Monte Alegre-SP | 350945 | 35.5 | 35.3 | 52.6 | 52.2 | 69.2 | 51.6 | 51.3 | 67.9 | 67.5 | 67.1 | 83.5 |
| Campinas-SP | 350950 | 113.3 | 117.5 | 117.2 | 121.6 | 133.4 | 132.3 | 137 | 141.3 | 146.6 | 147.1 | 142.7 |
| Campo Limpo Paulista-SP | 350960 | 25.7 | 22.7 | 19.8 | 23.4 | 29.5 | 35.5 | 33.8 | 47 | 42.8 | 49.5 | 46.6 |
| Campos do Jordão-SP | 350970 | 99.5 | 72 | 85.7 | 87.2 | 98.6 | 97.9 | 89.3 | 84.7 | 80.2 | 95.3 | 96.6 |
| Campos Novos Paulista-SP | 350980 | 65.5 | 43.3 | 86 | 85.3 | 84.7 | 105.1 | 104.3 | 82.8 | 82.2 | 61.2 | 60.8 |
| Cananéia-SP | 350990 | 71.8 | 111.7 | 71.8 | 111.7 | 135.6 | 135.6 | 127.6 | 143.6 | 143.6 | 143.6 | 135.6 |
| Canas-SP | 350995 | 46.1 | 45.3 | 44.5 | 21.9 | 21.5 | 42.4 | 41.8 | 20.6 | 20.3 | 40 | 39.4 |
| Cândido Mota-SP | 351000 | 36.1 | 39.3 | 45.7 | 55.4 | 52 | 51.9 | 51.7 | 54.8 | 51.5 | 61 | 60.9 |
| Cândido Rodrigues-SP | 351010 | 73.5 | 73.3 | 146.3 | 145.9 | 145.6 | 145.2 | 144.9 | 144.5 | 144.1 | 143.8 | 143.5 |
| Canitar-SP | 351015 | 23.3 | 22.8 | 22.3 | 21.9 | 21.5 | 21.1 | 62.3 | 61.2 | 60.2 | 79.1 | 38.9 |
| Capão Bonito-SP | 351020 | 40.1 | 35.9 | 40.1 | 40.2 | 38.1 | 42.3 | 44.4 | 50.8 | 57.2 | 53 | 74.2 |
| Capela do Alto-SP | 351030 | 23.1 | 34 | 50.1 | 65.6 | 64.5 | 74.1 | 46.9 | 66.6 | 90.8 | 94.4 | 93.1 |
| Capivari-SP | 351040 | 24.8 | 24.5 | 32.1 | 37.6 | 39.1 | 56 | 51.4 | 47 | 52 | 51.4 | 63.5 |
| Caraguatatuba-SP | 351050 | 41.4 | 51.4 | 60.9 | 77.8 | 78.1 | 104.1 | 128.1 | 126.7 | 129.6 | 136.8 | 150.5 |
| Carapicuíba-SP | 351060 | 26 | 25.3 | 40.4 | 44.1 | 47.9 | 50.5 | 49.9 | 53.9 | 56.6 | 41.1 | 55.4 |
| Cardoso-SP | 351070 | 33.2 | 33.1 | 33.1 | 74.2 | 74.1 | 73.9 | 73.8 | 90 | 89.8 | 97.7 | 97.5 |
| Casa Branca-SP | 351080 | 115 | 128.2 | 155.1 | 161.1 | 146.6 | 149.2 | 168.7 | 171.2 | 157 | 166.2 | 155.4 |
| Cássia dos Coqueiros-SP | 351090 | 72.9 | 36.7 | 37 | 37.3 | 37.6 | 75.8 | 114.5 | 115.4 | 77.5 | 156.2 | 118 |
| Castilho-SP | 351100 | 50.5 | 66.1 | 75.9 | 64 | 63 | 77.6 | 86.7 | 95.5 | 94.1 | 97.7 | 91.6 |
| Catanduva-SP | 351110 | 105.1 | 107.9 | 103.8 | 103.1 | 120.4 | 125.7 | 151.9 | 182.9 | 186.9 | 198.3 | 207.9 |
| Catiguá-SP | 351120 | 27.8 | 27.6 | 41.1 | 54.3 | 53.9 | 40.1 | 39.8 | 39.5 | 39.2 | 52 | 51.6 |
| Cedral-SP | 351130 | 50.6 | 62.2 | 61.2 | 72.4 | 83.2 | 82 | 80.9 | 113.9 | 112.4 | 99.9 | 98.6 |
| Cerqueira César-SP | 351140 | 51.5 | 56.4 | 72.3 | 54.9 | 54.3 | 53.6 | 74.1 | 109.9 | 113.7 | 127.8 | 101.1 |
| Cerquilho-SP | 351150 | 46.7 | 45.5 | 49.2 | 57.8 | 58.8 | 57.5 | 72.1 | 81.6 | 73.5 | 78.4 | 77 |
| Cesário Lange-SP | 351160 | 26 | 25.5 | 31.4 | 37.1 | 48.7 | 48 | 47.2 | 64 | 86 | 67.9 | 67 |
| Charqueada-SP | 351170 | 26.6 | 39.3 | 32.3 | 38.3 | 44.2 | 62.3 | 73.9 | 66.9 | 66.1 | 59.4 | 47 |
| Clementina-SP | 351190 | 29 | 42.4 | 69 | 67.6 | 66.2 | 51.9 | 38.1 | 37.4 | 36.7 | 36 | 35.4 |
| Colina-SP | 351200 | 34 | 33.8 | 56.2 | 39.1 | 39 | 55.5 | 60.8 | 49.5 | 32.9 | 32.7 | 54.4 |
| Colômbia-SP | 351210 | 97.9 | 146.7 | 146.5 | 130 | 129.9 | 162.2 | 178.2 | 145.6 | 145.4 | 161.4 | 161.2 |
| Conchal-SP | 351220 | 51.3 | 54.7 | 69.6 | 57.4 | 45.5 | 63.9 | 63.3 | 70.1 | 65.8 | 72.5 | 79.1 |
| Conchas-SP | 351230 | 61 | 66.5 | 77.9 | 77.2 | 76.6 | 70.2 | 58 | 74.8 | 108.5 | 90.7 | 101.3 |
| Cordeirópolis-SP | 351240 | 38.3 | 37.6 | 50.9 | 45.6 | 44.9 | 44.2 | 48 | 55.9 | 55.1 | 62.7 | 78.4 |
| Coroados-SP | 351250 | 76.9 | 94.6 | 93.1 | 91.8 | 90.5 | 71.4 | 70.4 | 69.4 | 68.5 | 67.7 | 66.8 |
| Coronel Macedo-SP | 351260 | 0 | 19.3 | 39 | 39.4 | 59.7 | 60.3 | 60.9 | 164.1 | 165.8 | 146.6 | 169.2 |
| Corumbataí-SP | 351270 | 50.6 | 75.7 | 75.5 | 75.4 | 100.3 | 125 | 124.7 | 124.4 | 124.1 | 123.8 | 173 |
| Cosmópolis-SP | 351280 | 22.7 | 20.4 | 31.5 | 37.3 | 36.5 | 41.9 | 39.6 | 40.2 | 38 | 27.3 | 26.8 |
| Cosmorama-SP | 351290 | 53.9 | 54 | 54.1 | 40.6 | 67.8 | 67.9 | 68 | 68.1 | 54.5 | 81.9 | 68.3 |
| Cotia-SP | 351300 | 29.1 | 31.8 | 29.1 | 35.1 | 61.1 | 59.8 | 62.5 | 73.3 | 73.5 | 78.7 | 83.8 |
| Cravinhos-SP | 351310 | 34.6 | 31.1 | 27.7 | 24.4 | 36.2 | 44.9 | 53.3 | 55.7 | 52.3 | 51.9 | 57.1 |
| Cristais Paulista-SP | 351320 | 52.8 | 52.1 | 77.1 | 63.5 | 75.3 | 86.8 | 98 | 60.5 | 47.9 | 71 | 82 |
| Cruzália-SP | 351330 | 125.3 | 169.3 | 128.7 | 130.3 | 132 | 178.3 | 180.7 | 183.2 | 232 | 235 | 238.1 |
| Cruzeiro-SP | 351340 | 42.2 | 47.1 | 58.2 | 65.5 | 74 | 78.7 | 83.3 | 79.2 | 60.4 | 66.2 | 72 |
| Cubatão-SP | 351350 | 83.7 | 80.4 | 101.1 | 105.1 | 107.5 | 99.4 | 102.6 | 121.4 | 122 | 124.2 | 108.7 |
| Cunha-SP | 351360 | 30.9 | 40 | 40.2 | 49.3 | 54 | 58.8 | 63.6 | 63.9 | 59.6 | 59.8 | 60.1 |
| Descalvado-SP | 351370 | 51 | 44.3 | 47.1 | 59.3 | 71.3 | 80.1 | 70.4 | 69.9 | 69.5 | 75 | 68.6 |
| Diadema-SP | 351380 | 67.1 | 72.6 | 76.6 | 81.5 | 85.8 | 81.9 | 82 | 89.1 | 89.7 | 87.6 | 88.4 |
| Dirce Reis-SP | 351385 | 116.6 | 116.1 | 115.5 | 115.1 | 114.6 | 114.2 | 113.7 | 113.3 | 112.8 | 56.2 | 56 |
| Divinolândia-SP | 351390 | 109.5 | 152.5 | 127.8 | 128.6 | 155.2 | 164.7 | 200.6 | 210.5 | 220.6 | 204.1 | 205.2 |
| Dobrada-SP | 351400 | 62.9 | 49.7 | 49.2 | 48.6 | 48.1 | 47.6 | 58.9 | 69.9 | 69.2 | 68.5 | 79.1 |
| Dois Córregos-SP | 351410 | 40.1 | 47.7 | 47.3 | 46.9 | 54.2 | 65.3 | 60.9 | 60.4 | 52.4 | 55.8 | 66.4 |
| Dolcinópolis-SP | 351420 | 46.4 | 46.4 | 46.5 | 93.2 | 93.4 | 93.6 | 140.6 | 93.9 | 94.1 | 47.1 | 47.2 |
| Dourado-SP | 351430 | 56.7 | 68 | 79.3 | 56.6 | 34 | 90.5 | 135.6 | 90.4 | 90.3 | 79 | 90.2 |
| Dracena-SP | 351440 | 64 | 68.1 | 78.9 | 96.4 | 104.7 | 95.2 | 107.8 | 109.3 | 113 | 118.9 | 118.2 |
| Duartina-SP | 351450 | 47.7 | 47.7 | 71.7 | 39.9 | 39.9 | 39.9 | 72 | 64 | 88.1 | 96.2 | 96.3 |
| Dumont-SP | 351460 | 37.6 | 61.3 | 59.9 | 58.7 | 46 | 56.4 | 44.3 | 43.4 | 42.6 | 73.3 | 61.8 |
| Echaporã-SP | 351470 | 60.9 | 61.3 | 61.8 | 62.2 | 62.6 | 78.7 | 95.1 | 95.8 | 128.6 | 129.4 | 162.8 |
| Eldorado-SP | 351480 | 53.7 | 60.2 | 53.3 | 46.5 | 66.1 | 65.9 | 65.7 | 65.4 | 97.8 | 84.5 | 97.1 |
| Elias Fausto-SP | 351490 | 38 | 31.3 | 30.9 | 36.7 | 30.2 | 41.9 | 35.5 | 35.1 | 40.6 | 45.9 | 51.1 |
| Elisiário-SP | 351492 | 32.4 | 31.8 | 31.3 | 30.8 | 30.3 | 29.8 | 29.4 | 28.9 | 28.5 | 28.1 | 27.7 |
| Embaúba-SP | 351495 | 40.1 | 40.2 | 40.3 | 121 | 161.5 | 161.7 | 121.5 | 81.1 | 121.8 | 122 | 122.1 |
| Embu das Artes-SP | 351500 | 22.5 | 26.3 | 28.4 | 33.7 | 35.2 | 44.2 | 45.6 | 43.2 | 44.5 | 44 | 49.8 |
| Embu-SPGuaçu-SP | 351510 | 31.7 | 31.4 | 32.6 | 33.9 | 33.6 | 40.9 | 45 | 59.5 | 48.7 | 49.8 | 59.5 |
| Emilianópolis-SP | 351512 | 65.2 | 64.9 | 64.6 | 64.3 | 64 | 63.8 | 63.5 | 63.2 | 63 | 62.7 | 62.5 |
| Engenheiro Coelho-SP | 351515 | 46.7 | 64.3 | 49.6 | 54.1 | 52.4 | 56.5 | 65.8 | 64 | 57 | 60.7 | 59.2 |
| Espírito Santo do Pinhal-SP | 351518 | 68 | 60.8 | 65.2 | 69.6 | 71.6 | 71.4 | 87.2 | 93.7 | 93.4 | 99.9 | 113.2 |
| Espírito Santo do Turvo-SP | 351519 | 47.2 | 46.6 | 91.9 | 90.8 | 89.7 | 88.6 | 65.7 | 64.9 | 64.2 | 63.5 | 62.8 |
| Estrela d'Oeste-SP | 351520 | 83.2 | 95.1 | 83.2 | 95.1 | 118.8 | 106.9 | 130.7 | 130.7 | 95 | 106.9 | 106.9 |
| Estrela do Norte-SP | 351530 | 73.7 | 73.5 | 73.4 | 73.3 | 73.2 | 73 | 109.3 | 109.2 | 72.6 | 72.5 | 108.6 |
| Euclides da Cunha Paulist-SP | 351535 | 70.4 | 81 | 71.2 | 81.8 | 72 | 113.7 | 93.6 | 73.2 | 94.6 | 95.1 | 116.8 |
| Fartura-SP | 351540 | 25.6 | 19.2 | 25.5 | 50.8 | 57 | 56.9 | 69.4 | 75.5 | 69.1 | 75.2 | 81.2 |
| Fernandópolis-SP | 351550 | 68.6 | 77.3 | 113.1 | 109.6 | 147.9 | 159.1 | 196.8 | 190 | 199.4 | 197 | 188.9 |
| Fernando Prestes-SP | 351560 | 53.1 | 35.3 | 52.9 | 52.8 | 87.8 | 87.6 | 69.9 | 104.6 | 104.4 | 121.5 | 104 |
| Fernão-SP | 351565 | 63.5 | 63 | 62.4 | 123.8 | 184.3 | 182.8 | 181.5 | 180 | 178.7 | 177.3 | 117.4 |
| Ferraz de Vasconcelos-SP | 351570 | 76.6 | 71.8 | 70.1 | 78.3 | 77.2 | 85 | 82.8 | 88.2 | 95 | 89.6 | 78.1 |
| Flora Rica-SP | 351580 | 159.8 | 163.3 | 167 | 113.7 | 290.5 | 297.1 | 303.8 | 248.9 | 318.5 | 325.9 | 266.8 |
| Floreal-SP | 351590 | 32.1 | 32.3 | 32.5 | 32.7 | 65.8 | 33.1 | 33.3 | 33.5 | 33.7 | 33.9 | 34.1 |
| Flórida Paulista-SP | 351600 | 70.3 | 69.3 | 83.5 | 75 | 74.1 | 95.1 | 94 | 100 | 105.9 | 118.6 | 124.3 |
| Florínia-SP | 351610 | 169.2 | 205 | 206.9 | 208.7 | 140.4 | 177.1 | 250.1 | 252.4 | 254.7 | 330.5 | 333.5 |
| Franca-SP | 351620 | 24.4 | 26.6 | 26 | 25.2 | 38.5 | 42.9 | 46.9 | 53.3 | 54 | 55.2 | 58.5 |
| Francisco Morato-SP | 351630 | 51.9 | 55.7 | 51.2 | 56.8 | 54.8 | 59.6 | 55.3 | 57.6 | 60.5 | 57.5 | 64.4 |
| Franco da Rocha-SP | 351640 | 53 | 52.8 | 49.7 | 59.8 | 102.6 | 115.9 | 121 | 102 | 137.6 | 143 | 131.2 |
| Gabriel Monteiro-SP | 351650 | 36 | 36 | 72 | 108.1 | 108.1 | 108.1 | 108.1 | 144.1 | 144.1 | 108.1 | 108.1 |
| Gália-SP | 351660 | 68.1 | 68.8 | 55.7 | 84.3 | 85.2 | 100.4 | 101.5 | 146.5 | 103.7 | 134.6 | 151.2 |
| Garça-SP | 351670 | 61.2 | 74.7 | 79.2 | 54.3 | 58.8 | 76.8 | 74.5 | 67.7 | 72.2 | 117.3 | 96.9 |
| Gastão Vidigal-SP | 351680 | 24 | 23.6 | 46.5 | 45.9 | 45.3 | 44.7 | 44.2 | 43.6 | 43.1 | 42.6 | 42.1 |
| Gavião Peixoto-SP | 351685 | 67.2 | 22.2 | 66.2 | 65.8 | 130.7 | 129.9 | 107.6 | 128.3 | 127.5 | 126.7 | 126 |
| General Salgado-SP | 351690 | 27.4 | 36.5 | 54.9 | 54.9 | 54.9 | 82.5 | 100.9 | 100.9 | 110.2 | 110.3 | 128.7 |
| Getulina-SP | 351700 | 27.4 | 36.4 | 54.4 | 45.1 | 89.9 | 89.6 | 98.2 | 88.9 | 79.7 | 79.4 | 88 |
| Glicério-SP | 351710 | 64.6 | 85.8 | 85.5 | 85.2 | 106.1 | 105.8 | 105.5 | 105.1 | 104.8 | 104.5 | 83.3 |
| Guaiçara-SP | 351720 | 28.2 | 27.8 | 27.4 | 27.1 | 26.7 | 26.4 | 26.1 | 43 | 42.5 | 42 | 41.5 |
| Guaimbê-SP | 351730 | 72.6 | 72.3 | 71.9 | 71.6 | 71.3 | 71 | 70.8 | 70.5 | 87.7 | 104.9 | 87.1 |
| Guaíra-SP | 351740 | 31.8 | 31.5 | 36.5 | 38.8 | 43.7 | 40.8 | 58.3 | 52.9 | 40 | 54.6 | 46.9 |
| Guapiaçu-SP | 351750 | 68.4 | 55.8 | 65.5 | 75 | 84.1 | 87.7 | 81.1 | 79.6 | 78.2 | 81.8 | 71 |
| Guapiara-SP | 351760 | 37.3 | 43 | 43.4 | 38.2 | 49.6 | 55.5 | 50.4 | 62.1 | 62.6 | 68.8 | 75.2 |
| Guará-SP | 351770 | 24.8 | 54.3 | 49.1 | 53.8 | 53.5 | 53.3 | 62.7 | 67.2 | 57.3 | 57 | 61.5 |
| Guaraçaí-SP | 351780 | 80.2 | 92.1 | 92.5 | 104.5 | 105 | 93.7 | 105.9 | 106.3 | 118.7 | 119.2 | 119.7 |
| Guaraci-SP | 351790 | 30 | 29.7 | 48.9 | 48.4 | 67 | 66.3 | 84.4 | 102.1 | 101.1 | 91 | 90.2 |
| Guarani d'Oeste-SP | 351800 | 49.4 | 99 | 99.1 | 99.2 | 99.3 | 99.4 | 99.5 | 199.2 | 149.6 | 149.7 | 149.9 |
| Guarantã-SP | 351810 | 61.2 | 45.8 | 76.2 | 60.8 | 91.1 | 75.8 | 90.8 | 90.6 | 90.5 | 75.3 | 105.2 |
| Guararapes-SP | 351820 | 19.4 | 25.7 | 25.5 | 31.7 | 41 | 37.6 | 49.9 | 58.9 | 58.6 | 64.4 | 70.2 |
| Guararema-SP | 351830 | 38.9 | 42.2 | 49.1 | 52.1 | 62.4 | 65.2 | 110.8 | 116.3 | 121.8 | 120.3 | 118.8 |
| Guaratinguetá-SP | 351840 | 35.3 | 57 | 65.3 | 66.6 | 72.1 | 68.2 | 87.3 | 105.2 | 129.6 | 118 | 131.3 |
| Guareí-SP | 351850 | 42.7 | 41.4 | 46.9 | 65.2 | 50.8 | 68.1 | 60.4 | 64.8 | 63.3 | 78.8 | 77.2 |
| Guariba-SP | 351860 | 28.2 | 27.8 | 27.5 | 32.6 | 26.9 | 34.5 | 42 | 41.6 | 48.8 | 45.8 | 47.8 |
| Guarujá-SP | 351870 | 35.5 | 37.9 | 37.9 | 44.9 | 50.5 | 52 | 58.1 | 62.1 | 82 | 85.8 | 88.6 |
| Guarulhos-SP | 351880 | 56.9 | 57.5 | 63.9 | 70.6 | 73.9 | 75.6 | 82.3 | 81.6 | 77.8 | 80.4 | 87.3 |
| Guatapará-SP | 351885 | 42.8 | 42.4 | 42 | 13.9 | 13.8 | 27.3 | 54.2 | 67.3 | 80.1 | 66.3 | 78.9 |
| Guzolândia-SP | 351890 | 20.9 | 41.5 | 41 | 40.7 | 40.3 | 39.9 | 59.4 | 78.5 | 77.8 | 57.9 | 38.3 |
| Herculândia-SP | 351900 | 45.6 | 45.3 | 44.9 | 44.5 | 55.2 | 65.8 | 76.2 | 86.4 | 85.7 | 85.1 | 95.1 |
| Holambra-SP | 351905 | 65 | 53.7 | 51.8 | 41.8 | 40.5 | 62.9 | 53.4 | 66.7 | 50.5 | 49.2 | 61.7 |
| Hortolândia-SP | 351907 | 27.5 | 30 | 30.9 | 33.3 | 31.2 | 37.3 | 38.1 | 45.7 | 43.1 | 48.3 | 56.3 |
| Iacanga-SP | 351910 | 50.4 | 49.6 | 48.7 | 47.9 | 66.1 | 74.4 | 100.7 | 99.2 | 88.9 | 87.7 | 77.9 |
| Iacri-SP | 351920 | 45.2 | 45.4 | 60.8 | 61.1 | 92 | 92.4 | 139.3 | 139.9 | 140.5 | 94.1 | 126 |
| Iaras-SP | 351925 | 34.1 | 32.2 | 61.2 | 87.7 | 56 | 53.7 | 77.3 | 49.5 | 59.7 | 46.2 | 33.5 |
| Ibaté-SP | 351930 | 45.7 | 41.8 | 41.3 | 34.5 | 34 | 33.6 | 33.2 | 35.8 | 47.1 | 46.6 | 51.8 |
| Ibirá-SP | 351940 | 46 | 63.5 | 62.7 | 26.5 | 43.7 | 69.1 | 59.7 | 67.4 | 66.7 | 65.9 | 73.4 |
| Ibirarema-SP | 351950 | 29.9 | 73.6 | 72.5 | 57.2 | 70.5 | 69.6 | 96.1 | 94.8 | 93.6 | 79.3 | 65.2 |
| Ibitinga-SP | 351960 | 22.6 | 22.3 | 31.2 | 34.5 | 37.7 | 49.7 | 47.4 | 46.8 | 51.5 | 66.3 | 62.2 |
| Ibiúna-SP | 351970 | 23.8 | 29.1 | 28.8 | 40.7 | 39 | 33.3 | 37 | 31.4 | 33.8 | 32.2 | 46 |
| Icém-SP | 351980 | 40 | 26.4 | 26.1 | 25.9 | 25.7 | 50.9 | 63.1 | 62.6 | 62.1 | 98.6 | 97.8 |
| Iepê-SP | 351990 | 51.7 | 51.4 | 76.7 | 76.4 | 88.6 | 100.8 | 112.9 | 99.8 | 86.9 | 111.3 | 135.4 |
| Igaraçu do Tietê-SP | 352000 | 12.6 | 8.4 | 8.4 | 16.6 | 24.9 | 28.9 | 16.5 | 16.4 | 4.1 | 8.2 | 8.1 |
| Igarapava-SP | 352010 | 21.3 | 24.6 | 27.9 | 27.7 | 41.3 | 51.3 | 74.7 | 80.9 | 90.4 | 96.5 | 95.9 |
| Igaratá-SP | 352020 | 33.6 | 33.4 | 55.2 | 87.8 | 98.2 | 86.8 | 75.5 | 75.1 | 85.3 | 95.4 | 94.9 |
| Iguape-SP | 352030 | 65 | 64.6 | 64.3 | 63.9 | 67 | 76.7 | 73 | 102.3 | 98.5 | 101.4 | 100.9 |
| Ilhabela-SP | 352040 | 76.6 | 78.1 | 90 | 81.1 | 92.5 | 96.9 | 107.4 | 108.2 | 112 | 121.7 | 151.5 |
| Ilha Comprida-SP | 352042 | 125.2 | 55.4 | 97.3 | 95.1 | 82.6 | 111.1 | 128.5 | 135.4 | 180 | 120.8 | 173.3 |
| Ilha Solteira-SP | 352044 | 90.4 | 86 | 93.4 | 108.5 | 119.6 | 122.9 | 145.4 | 137.1 | 144.1 | 158.6 | 158 |
| Indaiatuba-SP | 352050 | 29.6 | 26.3 | 26.6 | 28.3 | 31.4 | 31.1 | 49.3 | 49.1 | 77.1 | 78.9 | 76.5 |
| Indiana-SP | 352060 | 60.5 | 60.6 | 60.7 | 60.7 | 60.8 | 60.9 | 40.7 | 81.4 | 61.2 | 61.2 | 61.3 |
| Indiaporã-SP | 352070 | 99.3 | 124.6 | 125 | 125.3 | 150.9 | 252.1 | 278.2 | 228.3 | 229 | 229.7 | 204.7 |
| Inúbia Paulista-SP | 352080 | 82.1 | 81.3 | 53.7 | 53.3 | 52.9 | 52.5 | 52 | 77.4 | 76.8 | 76.3 | 75.7 |
| Ipaussu-SP | 352090 | 58.1 | 72 | 78.5 | 70.8 | 63.3 | 48.8 | 48.5 | 34.4 | 54.6 | 67.7 | 60.5 |
| Iperó-SP | 352100 | 40.7 | 42.8 | 34.5 | 40.1 | 32.4 | 40.9 | 42.8 | 47.6 | 43.4 | 48 | 49.6 |
| Ipeúna-SP | 352110 | 68.6 | 66.7 | 48.6 | 63.3 | 46.3 | 30.1 | 73.6 | 57.5 | 42.2 | 41.4 | 94.6 |
| Ipiguá-SP | 352115 | 68.6 | 67 | 65.6 | 64.3 | 63 | 61.8 | 60.7 | 59.5 | 97.5 | 95.8 | 113.1 |
| Iporanga-SP | 352120 | 157.2 | 135.4 | 113.5 | 159.6 | 229.1 | 207.2 | 208.2 | 162.8 | 187 | 328.8 | 236 |
| Ipuã-SP | 352130 | 35.6 | 63.1 | 69 | 61.1 | 60.3 | 52.8 | 78.1 | 115.5 | 120.3 | 112.5 | 117.2 |
| Iracemápolis-SP | 352140 | 25.5 | 39.8 | 48.7 | 43 | 42.1 | 32.1 | 45 | 53 | 65.1 | 59.7 | 54.5 |
| Irapuã-SP | 352150 | 0 | 13.5 | 40.2 | 26.6 | 26.4 | 26.2 | 26 | 51.5 | 51.1 | 50.8 | 50.4 |
| Irapuru-SP | 352160 | 63.2 | 88.1 | 100.2 | 124.7 | 111.7 | 123.6 | 110.8 | 110.3 | 109.8 | 97.2 | 96.8 |
| Itaberá-SP | 352170 | 48.7 | 59.8 | 65.5 | 60.4 | 66.2 | 66.5 | 77.9 | 95.1 | 73 | 73.4 | 79.4 |
| Itaí-SP | 352180 | 37.5 | 49.4 | 44.7 | 32.1 | 27.8 | 35.3 | 50.5 | 49.9 | 53.2 | 56.4 | 63.3 |
| Itajobi-SP | 352190 | 53.9 | 47 | 60.3 | 73.5 | 100 | 79.8 | 92.9 | 99.2 | 99 | 105.3 | 98.5 |
| Itaju-SP | 352200 | 62.4 | 61.2 | 60.1 | 59.1 | 58.1 | 85.7 | 112.5 | 110.6 | 108.9 | 107.3 | 105.8 |
| Itanhaém-SP | 352210 | 39.5 | 70.7 | 63.9 | 71.7 | 76 | 79.1 | 74.8 | 79.9 | 87.9 | 89.8 | 122.4 |
| Itaóca-SP | 352215 | 60.5 | 60.5 | 30.2 | 60.4 | 60.4 | 30.2 | 30.1 | 30.1 | 30.1 | 30.1 | 60.1 |
| Itapecerica da Serra-SP | 352220 | 77.8 | 77.9 | 81.2 | 87.6 | 90.2 | 93.9 | 97.5 | 106.9 | 103.2 | 114.8 | 130.7 |
| Itapetininga-SP | 352230 | 36.8 | 38.3 | 37.8 | 47.4 | 57.4 | 54.1 | 61.9 | 67.5 | 76.2 | 83.5 | 80.7 |
| Itapeva-SP | 352240 | 46.1 | 50.3 | 68.9 | 92.9 | 107.7 | 106.1 | 117.5 | 136.3 | 134.5 | 137 | 143.8 |
| Itapevi-SP | 352250 | 76.8 | 67.8 | 70.5 | 71.2 | 68.1 | 65.5 | 68.1 | 71.9 | 67.3 | 79.2 | 80.2 |
| Itapira-SP | 352260 | 55 | 57.4 | 55.5 | 65 | 72.9 | 69.6 | 80.2 | 86.5 | 88.6 | 88.1 | 90.2 |
| Itapirapuã Paulista-SP | 352265 | 76.7 | 76 | 50.3 | 74.9 | 74.3 | 49.2 | 122.1 | 96.9 | 96.2 | 71.7 | 71.2 |
| Itápolis-SP | 352270 | 22.2 | 22.1 | 41.4 | 55.7 | 50.6 | 50.3 | 50 | 52.1 | 54.2 | 44.5 | 55.9 |
| Itaporanga-SP | 352280 | 33.7 | 26.9 | 46.9 | 33.5 | 33.4 | 66.7 | 93.2 | 93 | 92.9 | 92.7 | 86 |
| Itapuí-SP | 352290 | 90.8 | 81.3 | 88.1 | 87 | 78 | 84.7 | 83.6 | 82.5 | 81.4 | 65.8 | 57.8 |
| Itapura-SP | 352300 | 45.9 | 90.7 | 67.2 | 66.4 | 65.7 | 65 | 85.7 | 63.6 | 63 | 62.3 | 61.7 |
| Itaquaquecetuba-SP | 352310 | 31.9 | 36 | 36.1 | 34.1 | 35.7 | 34.9 | 34.4 | 40.2 | 48.1 | 43.6 | 49.7 |
| Itararé-SP | 352320 | 45.1 | 44.9 | 52.9 | 64.9 | 64.7 | 66.5 | 64.3 | 70.1 | 67.9 | 73.7 | 63.5 |
| Itariri-SP | 352330 | 45.2 | 38.3 | 44.1 | 56.1 | 55.5 | 67.1 | 60.3 | 53.7 | 59.1 | 64.3 | 75.3 |
| Itatiba-SP | 352340 | 36 | 44.1 | 49 | 54.8 | 72.3 | 69.3 | 78.8 | 88.9 | 86.6 | 79.3 | 83.1 |
| Itatinga-SP | 352350 | 27.8 | 43.9 | 37.8 | 37.3 | 42.1 | 41.6 | 51.3 | 35.4 | 30 | 39.5 | 39.1 |
| Itirapina-SP | 352360 | 32.5 | 32 | 44 | 61.9 | 60.9 | 72 | 59.1 | 64 | 57.3 | 62.2 | 61.4 |
| Itirapuã-SP | 352370 | 50.4 | 49.9 | 49.5 | 32.7 | 32.5 | 32.2 | 47.9 | 63.4 | 47.2 | 46.8 | 46.5 |
| Itobi-SP | 352380 | 13 | 12.9 | 12.9 | 38.7 | 64.4 | 64.3 | 89.9 | 77 | 76.9 | 76.7 | 63.9 |
| Itu-SP | 352390 | 49.3 | 63.4 | 65.8 | 76.3 | 76.1 | 76.5 | 87.2 | 73 | 100.1 | 76.2 | 90.6 |
| Itupeva-SP | 352400 | 33.1 | 36.3 | 39.1 | 44 | 40.4 | 46.9 | 45.4 | 43.9 | 72.7 | 74.1 | 72.1 |
| Ituverava-SP | 352410 | 76.7 | 78.7 | 73.1 | 80.2 | 92.1 | 99 | 96 | 112.5 | 109.4 | 113.6 | 134.6 |
| Jaborandi-SP | 352420 | 44.7 | 59.4 | 44.4 | 59 | 58.8 | 44 | 43.9 | 87.5 | 72.7 | 87.1 | 101.3 |
| Jaboticabal-SP | 352430 | 40 | 39.7 | 38.1 | 46 | 51.1 | 74.9 | 77.1 | 75.4 | 81.5 | 87.6 | 83.3 |
| Jacareí-SP | 352440 | 30.1 | 29.8 | 31.4 | 23.3 | 24.5 | 31 | 67.3 | 73.8 | 95.5 | 85.2 | 101.8 |
| Jaci-SP | 352450 | 200.4 | 212.6 | 189.7 | 252.4 | 230 | 224.7 | 298.1 | 245.4 | 255.2 | 294.2 | 288.4 |
| Jacupiranga-SP | 352460 | 22.7 | 34.1 | 51 | 56.6 | 62.2 | 56.4 | 56.4 | 95.7 | 73.1 | 95.4 | 89.7 |
| Jaguariúna-SP | 352470 | 51.8 | 72.8 | 66 | 70.5 | 72.7 | 96.9 | 110.1 | 103.3 | 104.4 | 103.8 | 104.9 |
| Jales-SP | 352480 | 91.7 | 95.7 | 99.6 | 101.5 | 111.6 | 121.6 | 129.6 | 141.6 | 149.5 | 190.1 | 195.9 |
| Jambeiro-SP | 352490 | 57.6 | 56.1 | 73 | 71.3 | 87.2 | 85.3 | 100.2 | 114.4 | 144.1 | 157.1 | 123.4 |
| Jandira-SP | 352500 | 24.1 | 23.8 | 35.1 | 35.5 | 36.8 | 30.2 | 30.7 | 39.5 | 31.5 | 36.9 | 38.1 |
| Jardinópolis-SP | 352510 | 37.6 | 55.4 | 54.4 | 76.4 | 60.1 | 64.1 | 67.9 | 78.8 | 84.6 | 81.1 | 91.4 |
| Jarinu-SP | 352520 | 26 | 21 | 36.8 | 23.9 | 46.7 | 72.1 | 70.4 | 79.6 | 84.9 | 79.7 | 88.3 |
| Jaú-SP | 352530 | 151 | 162.4 | 186.1 | 200.5 | 199.4 | 207.5 | 240.2 | 252.5 | 252.8 | 248.5 | 246.3 |
| Jeriquara-SP | 352540 | 61.4 | 61.6 | 61.7 | 61.9 | 62.1 | 31.1 | 31.2 | 31.3 | 62.8 | 63 | 63.1 |
| Joanópolis-SP | 352550 | 17 | 8.4 | 16.6 | 32.8 | 32.5 | 64.2 | 55.6 | 55 | 46.7 | 69.4 | 61.1 |
| João Ramalho-SP | 352560 | 47.8 | 47.4 | 117.5 | 93.4 | 92.7 | 115.1 | 91.4 | 113.5 | 112.7 | 134.3 | 155.7 |
| José Bonifácio-SP | 352570 | 36.6 | 45.2 | 38.7 | 38.3 | 46.6 | 54.7 | 51.2 | 61.9 | 55.7 | 57.9 | 60 |
| Júlio Mesquita-SP | 352580 | 66.9 | 88.7 | 110.1 | 109.5 | 108.8 | 86.6 | 86.1 | 85.6 | 85.1 | 105.8 | 105.2 |
| Jumirim-SP | 352585 | 72.8 | 71.3 | 34.9 | 34.2 | 33.5 | 98.8 | 97 | 95.2 | 93.5 | 92 | 60.3 |
| Jundiaí-SP | 352590 | 56 | 53.4 | 58.8 | 58.1 | 65.9 | 71.8 | 79 | 89.6 | 103.6 | 101.3 | 107.3 |
| Junqueirópolis-SP | 352600 | 69 | 73.6 | 93.8 | 93 | 107.5 | 111.7 | 115.8 | 109.8 | 128.7 | 152.2 | 136.4 |
| Juquiá-SP | 352610 | 50.1 | 70.6 | 81.1 | 71.3 | 87.1 | 72.1 | 82.8 | 72.9 | 73.3 | 73.7 | 63.5 |
| Juquitiba-SP | 352620 | 58.7 | 61.6 | 47.5 | 40.4 | 36.8 | 36.5 | 46.1 | 42.5 | 42.2 | 54.8 | 51.2 |
| Lagoinha-SP | 352630 | 60.3 | 80.5 | 100.7 | 100.9 | 121.3 | 121.5 | 121.6 | 121.8 | 122 | 142.6 | 142.8 |
| Laranjal Paulista-SP | 352640 | 31.7 | 27.4 | 34.8 | 30.6 | 34 | 44.8 | 59.1 | 58.4 | 47 | 46.5 | 81.4 |
| Lavínia-SP | 352650 | 48.4 | 69.5 | 77.8 | 75 | 82.7 | 79.9 | 96.6 | 112.2 | 99.8 | 96.9 | 85.7 |
| Lavrinhas-SP | 352660 | 45.2 | 44.8 | 59.2 | 58.7 | 58.2 | 72.2 | 57.3 | 56.8 | 70.4 | 83.9 | 83.3 |
| Leme-SP | 352670 | 40.3 | 42 | 48.9 | 58.9 | 67.6 | 66.9 | 65.1 | 63.4 | 76.7 | 73 | 83 |
| Lençóis Paulista-SP | 352680 | 55.2 | 57.8 | 60.3 | 58.2 | 62.3 | 72.5 | 74.9 | 84.8 | 85.5 | 84.7 | 88.4 |
| Limeira-SP | 352690 | 31.7 | 32.5 | 35.7 | 39.2 | 40.6 | 51.2 | 60.3 | 73.3 | 74.7 | 84.7 | 85.3 |
| Lindóia-SP | 352700 | 60.5 | 44.5 | 58.1 | 85.7 | 84.2 | 82.7 | 81.3 | 106.6 | 91.8 | 90.4 | 63.6 |
| Lins-SP | 352710 | 116.6 | 114.3 | 103.8 | 84 | 79.4 | 84.2 | 110.1 | 108 | 126.8 | 131.2 | 123.9 |
| Lorena-SP | 352720 | 45.5 | 51.1 | 57.9 | 48.2 | 62 | 72.1 | 93.7 | 113.9 | 106.4 | 108.2 | 120.1 |
| Lourdes-SP | 352725 | 92.9 | 92.3 | 45.9 | 45.6 | 90.7 | 90.2 | 134.5 | 89.2 | 88.7 | 44.1 | 87.8 |
| Louveira-SP | 352730 | 19.8 | 43.6 | 52.6 | 56.1 | 74.1 | 84 | 88.6 | 99.6 | 103.6 | 101 | 92.3 |
| Lucélia-SP | 352740 | 64.8 | 74.2 | 88.3 | 97.4 | 101.5 | 95.9 | 109.5 | 122.9 | 112.6 | 130.5 | 111.1 |
| Lucianópolis-SP | 352750 | 43.8 | 43.6 | 86.7 | 86.4 | 129 | 128.4 | 85.3 | 84.9 | 84.5 | 84.2 | 83.9 |
| Luís Antônio-SP | 352760 | 37.2 | 26.9 | 17.3 | 25.1 | 24.3 | 23.6 | 15.3 | 59.3 | 57.7 | 91.4 | 89.1 |
| Luiziânia-SP | 352770 | 40 | 39.4 | 38.8 | 76.5 | 56.6 | 55.9 | 55.1 | 54.4 | 53.7 | 106.1 | 104.8 |
| Lupércio-SP | 352780 | 45.1 | 67.5 | 67.2 | 89.4 | 89.1 | 66.6 | 66.4 | 66.2 | 66 | 65.8 | 65.6 |
| Lutécia-SP | 352790 | 106.6 | 107.2 | 107.8 | 144.6 | 181.7 | 182.7 | 183.7 | 184.7 | 222.9 | 224 | 225.3 |
| Macatuba-SP | 352800 | 60.4 | 48.2 | 54 | 89.7 | 89.4 | 83.2 | 71 | 59 | 70.6 | 64.5 | 76 |
| Macaubal-SP | 352810 | 25.7 | 25.6 | 25.5 | 25.4 | 25.3 | 50.3 | 75.2 | 62.5 | 99.6 | 99.2 | 111.2 |
| Macedônia-SP | 352820 | 53.1 | 53.1 | 53.2 | 106.7 | 106.9 | 107.1 | 134 | 134.3 | 161.5 | 161.7 | 162 |
| Magda-SP | 352830 | 60.3 | 60.6 | 61 | 92 | 92.5 | 93 | 31.2 | 31.4 | 31.5 | 31.7 | 63.8 |
| Mairinque-SP | 352840 | 41.3 | 45.5 | 40.6 | 17.9 | 17.8 | 35.3 | 70.2 | 67.5 | 58.4 | 66.6 | 55.5 |
| Mairiporã-SP | 352850 | 33 | 35.9 | 34.9 | 31.8 | 39.1 | 40.5 | 40.8 | 37.7 | 34.9 | 37.3 | 38.6 |
| Manduri-SP | 352860 | 11 | 32.8 | 43.4 | 43.1 | 42.7 | 42.4 | 42.1 | 52.2 | 62.2 | 61.8 | 71.6 |
| Marabá Paulista-SP | 352870 | 63.7 | 83 | 101.4 | 119.1 | 116.7 | 95.3 | 112.1 | 91.6 | 89.9 | 88.4 | 86.9 |
| Maracaí-SP | 352880 | 36.8 | 44 | 43.9 | 51.1 | 65.5 | 58.1 | 65.1 | 86.6 | 79.2 | 86.1 | 85.9 |
| Marapoama-SP | 352885 | 38.2 | 37.6 | 37.1 | 36.5 | 72.1 | 106.7 | 140.4 | 138.6 | 136.8 | 101.4 | 100.1 |
| Mariápolis-SP | 352890 | 25 | 25 | 24.9 | 24.9 | 74.4 | 99.1 | 98.9 | 98.7 | 98.5 | 98.3 | 98.1 |
| Marília-SP | 352900 | 101.8 | 107.7 | 119.7 | 123.2 | 138.5 | 154 | 170.2 | 175.7 | 198.7 | 206.1 | 204.1 |
| Marinópolis-SP | 352910 | 91.8 | 92 | 92.3 | 138.9 | 92.9 | 186.3 | 93.4 | 93.7 | 140.9 | 141.3 | 141.7 |
| Martinópolis-SP | 352920 | 61.4 | 56.8 | 60.4 | 68 | 83.3 | 82.7 | 78.2 | 77.7 | 81 | 76.6 | 79.9 |
| Matão-SP | 352930 | 37.4 | 44.8 | 50.8 | 56.8 | 67.7 | 83.5 | 86.7 | 97.2 | 105.2 | 96.1 | 107.6 |
| Mauá-SP | 352940 | 31.2 | 31.5 | 40 | 45.1 | 51.9 | 57.8 | 68.1 | 65.1 | 73.1 | 73 | 71.2 |
| Mendonça-SP | 352950 | 21.8 | 21.4 | 21 | 20.7 | 81.3 | 79.9 | 59 | 96.6 | 95.1 | 93.7 | 129.3 |
| Meridiano-SP | 352960 | 50.3 | 50.4 | 50.6 | 50.8 | 127.4 | 127.8 | 128.2 | 128.7 | 129.1 | 129.5 | 129.9 |
| Mesópolis-SP | 352965 | 0 | 103.3 | 103.5 | 103.6 | 103.8 | 104 | 104.1 | 104.2 | 104.4 | 104.5 | 157.1 |
| Miguelópolis-SP | 352970 | 19.4 | 62.5 | 66.8 | 75.8 | 94.1 | 98.2 | 111.4 | 110.7 | 68.7 | 59.2 | 58.8 |
| Mineiros do Tietê-SP | 352980 | 41 | 40.7 | 40.5 | 40.3 | 32.1 | 31.9 | 31.8 | 39.5 | 55 | 54.7 | 54.5 |
| Miracatu-SP | 352990 | 42 | 42.3 | 37.9 | 42.9 | 48.1 | 53.3 | 53.6 | 113 | 108.9 | 109.7 | 100.4 |
| Mira Estrela-SP | 353000 | 70.3 | 69.8 | 103.8 | 103 | 102.2 | 135.3 | 167.8 | 133.2 | 132.3 | 98.5 | 97.8 |
| Mirandópolis-SP | 353010 | 86.2 | 92.8 | 95.8 | 95.3 | 112.3 | 118.7 | 138.9 | 145 | 140.8 | 136.7 | 119 |
| Mirante do Paranapanema-SP | 353020 | 75.1 | 69 | 74.3 | 74 | 90.6 | 78.9 | 78.5 | 94.8 | 94.4 | 88.4 | 88 |
| Mirassol-SP | 353030 | 42.6 | 42.1 | 43.5 | 44.9 | 49.8 | 44.1 | 47.2 | 45 | 51.4 | 49.3 | 53.9 |
| Mirassolândia-SP | 353040 | 23.3 | 23 | 45.4 | 89.8 | 110.9 | 87.7 | 86.7 | 85.7 | 127.1 | 104.8 | 62.2 |
| Mococa-SP | 353050 | 34 | 41.3 | 61.8 | 64.6 | 64.5 | 65.9 | 74.6 | 77.4 | 75.8 | 74.2 | 95.9 |
| Mogi das Cruzes-SP | 353060 | 50.6 | 65.4 | 69.9 | 67.7 | 78.6 | 80.9 | 84.9 | 91.8 | 106.9 | 114.6 | 114.8 |
| Mogi Guaçu-SP | 353070 | 44.2 | 42.3 | 46.9 | 57.8 | 57.9 | 64.4 | 80.3 | 80.2 | 80.2 | 76.9 | 77 |
| Mogi Mirim-SP | 353080 | 62.8 | 72.6 | 85.7 | 86.3 | 79.2 | 98.7 | 100.3 | 97.5 | 99.2 | 91.1 | 87.4 |
| Mombuca-SP | 353090 | 60.4 | 60.1 | 59.7 | 89.2 | 88.7 | 58.9 | 58.6 | 58.3 | 58 | 57.8 | 57.5 |
| Monções-SP | 353100 | 46.1 | 45.9 | 91.5 | 91.2 | 90.8 | 90.5 | 135.2 | 134.6 | 134.2 | 133.7 | 133.2 |
| Mongaguá-SP | 353110 | 26.6 | 34.5 | 29.5 | 39.2 | 40.4 | 37.6 | 40.7 | 37.9 | 40.9 | 49.3 | 57.4 |
| Monte Alegre do Sul-SP | 353120 | 27.9 | 41.4 | 40.9 | 54 | 53.4 | 52.8 | 65.3 | 64.6 | 64 | 76.1 | 75.3 |
| Monte Alto-SP | 353130 | 44.5 | 50.5 | 50.2 | 49.9 | 57.8 | 57.5 | 49 | 46.6 | 50.4 | 52.1 | 57.8 |
| Monte Aprazível-SP | 353140 | 50.9 | 50.1 | 49.3 | 61.9 | 69.8 | 77.4 | 89.1 | 75.4 | 86.8 | 93.9 | 88.7 |
| Monte Azul Paulista-SP | 353150 | 30.8 | 25.7 | 30.9 | 31 | 46.6 | 62.3 | 52 | 62.6 | 78.4 | 89 | 99.7 |
| Monte Castelo-SP | 353160 | 72 | 72 | 96 | 96 | 96 | 96 | 96 | 96 | 120 | 96 | 96 |
| Monteiro Lobato-SP | 353170 | 48.6 | 47.9 | 23.7 | 46.8 | 138.9 | 137.3 | 158.4 | 111.9 | 110.7 | 131.5 | 108.5 |
| Monte Mor-SP | 353180 | 41.8 | 44.9 | 45.8 | 44.9 | 51.6 | 54.3 | 55 | 41.3 | 47.6 | 46.8 | 56.2 |
| Morro Agudo-SP | 353190 | 37.8 | 40.7 | 46.9 | 46.4 | 49.1 | 48.5 | 41.6 | 44.3 | 62.6 | 61.9 | 61.3 |
| Morungaba-SP | 353200 | 17.1 | 33.7 | 24.9 | 40.8 | 48.3 | 39.7 | 62.6 | 38.6 | 53.3 | 45.1 | 52 |
| Motuca-SP | 353205 | 69.6 | 68.9 | 68.2 | 67.6 | 44.6 | 88.5 | 131.6 | 108.6 | 150.8 | 128.2 | 127.1 |
| Murutinga do Sul-SP | 353210 | 141.4 | 140.6 | 116.5 | 115.9 | 115.3 | 160.7 | 91.3 | 90.9 | 113 | 180 | 156.7 |
| Nantes-SP | 353215 | 37.2 | 36.6 | 72.1 | 142 | 140 | 138 | 170 | 134.1 | 165.3 | 163.2 | 128.9 |
| Narandiba-SP | 353220 | 70 | 115.2 | 113.7 | 112.4 | 88.9 | 131.8 | 130.3 | 150.3 | 148.7 | 105.1 | 104 |
| Natividade da Serra-SP | 353230 | 14.5 | 29.1 | 29.2 | 29.3 | 44.1 | 44.2 | 59.2 | 59.3 | 44.6 | 44.8 | 59.9 |
| Nazaré Paulista-SP | 353240 | 18.3 | 12 | 11.9 | 5.9 | 5.8 | 5.7 | 5.7 | 16.9 | 16.7 | 11 | 54.5 |
| Neves Paulista-SP | 353250 | 77.7 | 66.7 | 77.8 | 66.8 | 55.7 | 66.9 | 66.9 | 67 | 67 | 55.9 | 56 |
| Nhandeara-SP | 353260 | 119.5 | 118.9 | 109.2 | 108.6 | 135.1 | 116.5 | 124.9 | 133.1 | 132.5 | 123.1 | 131.3 |
| Nipoã-SP | 353270 | 95.8 | 70.1 | 45.7 | 44.7 | 87.5 | 107.2 | 105.1 | 103 | 101.1 | 79.4 | 78 |
| Nova Aliança-SP | 353280 | 51.6 | 67.5 | 66.2 | 65.1 | 64 | 62.9 | 61.9 | 76.1 | 74.9 | 73.8 | 72.7 |
| Nova Campina-SP | 353282 | 35.4 | 34.9 | 34.4 | 56.5 | 44.6 | 44.1 | 32.6 | 43 | 53 | 62.9 | 62.2 |
| Nova Canaã Paulista-SP | 353284 | 44.7 | 45.4 | 46.1 | 46.8 | 47.6 | 48.3 | 49.1 | 99.8 | 101.4 | 154.5 | 157 |
| Nova Castilho-SP | 353286 | 266.7 | 175.6 | 260.2 | 257.3 | 254.5 | 251.7 | 166.1 | 164.2 | 162.6 | 160.9 | 159.4 |
| Nova Europa-SP | 353290 | 54.8 | 53.6 | 52.4 | 61.7 | 60.6 | 69.3 | 77.8 | 76.4 | 75.1 | 64.6 | 72.6 |
| Nova Granada-SP | 353300 | 57.3 | 66.9 | 55.9 | 65.4 | 49.8 | 59.2 | 78.1 | 67.6 | 66.9 | 61.6 | 61 |
| Nova Guataporanga-SP | 353310 | 45.2 | 90 | 89.6 | 44.6 | 44.4 | 88.5 | 88.1 | 87.7 | 87.4 | 87 | 86.7 |
| Nova Independência-SP | 353320 | 34 | 32.9 | 31.8 | 30.9 | 60 | 87.6 | 142.1 | 110.7 | 135 | 105.4 | 103 |
| Novais-SP | 353325 | 0 | 0 | 0 | 0 | 0 | 0 | 76.7 | 74.8 | 73.1 | 71.5 | 87.5 |
| Nova Luzitânia-SP | 353330 | 29.5 | 28.9 | 28.4 | 83.5 | 109.4 | 134.3 | 105.6 | 103.8 | 102.1 | 100.5 | 99 |
| Nova Odessa-SP | 353340 | 21.7 | 15.5 | 13.3 | 22.5 | 20.3 | 18.1 | 23.2 | 28.1 | 24.3 | 35.9 | 38.7 |
| Novo Horizonte-SP | 353350 | 27.3 | 32.4 | 40 | 36.9 | 39.1 | 38.7 | 61.4 | 63.3 | 72.6 | 69.5 | 68.8 |
| Nuporanga-SP | 353360 | 29.1 | 28.8 | 42.9 | 56.8 | 56.4 | 112.1 | 139.1 | 138.1 | 137.2 | 136.3 | 121.9 |
| Ocauçu-SP | 353370 | 46.9 | 70.3 | 70.3 | 70.3 | 70.2 | 70.2 | 93.5 | 46.7 | 46.7 | 46.7 | 46.7 |
| Óleo-SP | 353380 | 0 | 0 | 0 | 0 | 37.2 | 37.6 | 0 | 38.4 | 38.9 | 39.2 | 39.7 |
| Olímpia-SP | 353390 | 27.8 | 29.5 | 27.3 | 31 | 71.1 | 83.9 | 88.9 | 107 | 110 | 99.9 | 101.1 |
| Onda Verde-SP | 353400 | 51.5 | 25.4 | 25.1 | 24.8 | 49.1 | 48.6 | 72.1 | 142.6 | 164.6 | 139.6 | 92.2 |
| Oriente-SP | 353410 | 32.3 | 32.2 | 32 | 47.8 | 63.4 | 47.3 | 62.8 | 78.1 | 77.8 | 92.9 | 92.5 |
| Orindiúva-SP | 353420 | 18.1 | 35.3 | 34.4 | 33.6 | 98.3 | 112.1 | 94 | 76.6 | 75 | 102.9 | 86.5 |
| Orlândia-SP | 353430 | 42.5 | 34.7 | 34.3 | 46.2 | 53 | 62.1 | 61.5 | 58.6 | 58.2 | 53.1 | 54.9 |
| Osasco-SP | 353440 | 59.7 | 62.2 | 62.6 | 75.5 | 72.8 | 67.5 | 79.7 | 78.5 | 93.4 | 89.3 | 86.5 |
| Oscar Bressane-SP | 353450 | 76.9 | 76.9 | 115.3 | 115.3 | 115.3 | 115.3 | 230.6 | 230.6 | 230.6 | 269 | 307.5 |
| Osvaldo Cruz-SP | 353460 | 82.8 | 82.4 | 75.7 | 94.3 | 109.5 | 102.8 | 105.5 | 92.7 | 95.4 | 113.4 | 125.2 |
| Ourinhos-SP | 353470 | 47.3 | 47.8 | 51.1 | 58.2 | 59.6 | 89.6 | 97.1 | 127.1 | 126.1 | 109.1 | 117.1 |
| Ouroeste-SP | 353475 | 85.5 | 83.3 | 81.2 | 113.5 | 111 | 119.4 | 106.3 | 114.5 | 153 | 140.1 | 157.2 |
| Ouro Verde-SP | 353480 | 25.5 | 25.2 | 25 | 37.2 | 36.9 | 36.6 | 48.5 | 48.1 | 47.7 | 82.9 | 70.6 |
| Pacaembu-SP | 353490 | 82.1 | 81.6 | 81.1 | 88 | 94.9 | 101.6 | 101.1 | 100.6 | 100.1 | 135.1 | 134.5 |
| Palestina-SP | 353500 | 27.4 | 27 | 17.7 | 26.1 | 34.2 | 42.1 | 58 | 49 | 32.2 | 55.5 | 39.1 |
| Palmares Paulista-SP | 353510 | 18.7 | 18.3 | 26.8 | 43.7 | 42.8 | 42 | 32.9 | 48.5 | 47.6 | 46.7 | 38.3 |
| Palmeira d'Oeste-SP | 353520 | 30.1 | 30.3 | 61.1 | 61.5 | 61.8 | 62.2 | 104.4 | 105.1 | 116.3 | 106.4 | 64.2 |
| Palmital-SP | 353530 | 55.6 | 60 | 87.5 | 96.4 | 105.3 | 109.6 | 109.4 | 109.1 | 108.8 | 99.5 | 108.3 |
| Panorama-SP | 353540 | 40.7 | 67.3 | 73.6 | 119.7 | 132.1 | 131.3 | 117.5 | 116.7 | 128.9 | 121.8 | 140.2 |
| Paraguaçu Paulista-SP | 353550 | 49.1 | 58.1 | 60 | 71.1 | 72.9 | 77 | 78.8 | 80.6 | 93.4 | 84.1 | 92.4 |
| Paraibuna-SP | 353560 | 39.5 | 61.9 | 61.7 | 56 | 61.4 | 66.8 | 66.6 | 72 | 77.4 | 71.7 | 82.5 |
| Paraíso-SP | 353570 | 33.6 | 33.4 | 33.1 | 32.8 | 32.6 | 32.3 | 32.1 | 47.8 | 63.3 | 62.8 | 46.8 |
| Paranapanema-SP | 353580 | 50.6 | 50 | 60.3 | 37.9 | 37.4 | 42.3 | 41.8 | 46.5 | 35.8 | 40.4 | 50 |
| Paranapuã-SP | 353590 | 51.7 | 51.4 | 51.1 | 50.9 | 76 | 50.4 | 50.2 | 25 | 49.7 | 49.5 | 49.2 |
| Parapuã-SP | 353600 | 53.8 | 62.9 | 45 | 72.1 | 81.2 | 90.4 | 90.5 | 99.7 | 90.8 | 100 | 82 |
| Pardinho-SP | 353610 | 18 | 17.7 | 17.5 | 17.2 | 68 | 16.8 | 16.5 | 49 | 48.3 | 47.7 | 62.9 |
| Pariquera-SPAçu-SP | 353620 | 208.2 | 265.7 | 296.2 | 373.8 | 366.9 | 386.2 | 405.3 | 434.6 | 458.5 | 425.8 | 454.8 |
| Parisi-SP | 353625 | 96.9 | 144.7 | 96 | 47.8 | 47.6 | 47.4 | 47.2 | 94 | 93.6 | 93.2 | 92.9 |
| Patrocínio Paulista-SP | 353630 | 61.5 | 76 | 75 | 66.8 | 66 | 79.8 | 107.6 | 99.4 | 91.3 | 90.4 | 103.2 |
| Paulicéia-SP | 353640 | 31.8 | 31.3 | 30.8 | 30.3 | 29.9 | 44.2 | 58 | 85.8 | 98.8 | 153.2 | 137.5 |
| Paulínia-SP | 353650 | 53.8 | 50.5 | 51.1 | 48.2 | 47.8 | 58.2 | 75.3 | 77.1 | 121.4 | 129.7 | 123.6 |
| Paulistânia-SP | 353657 | 54.9 | 54.9 | 54.8 | 54.8 | 54.8 | 54.7 | 54.7 | 54.7 | 0 | 54.6 | 54.6 |
| Paulo de Faria-SP | 353660 | 34.2 | 56.9 | 56.8 | 45.3 | 45.3 | 67.8 | 67.7 | 67.5 | 67.4 | 67.3 | 100.8 |
| Pederneiras-SP | 353670 | 21.7 | 23.8 | 25.9 | 30.2 | 34.5 | 41 | 42.8 | 46.8 | 41.9 | 52.4 | 47.6 |
| Pedra Bela-SP | 353680 | 34 | 33.9 | 33.8 | 16.8 | 33.5 | 33.4 | 50 | 16.6 | 16.6 | 16.5 | 32.9 |
| Pedranópolis-SP | 353690 | 37.7 | 75.8 | 76.3 | 76.7 | 77.1 | 77.5 | 39 | 117.7 | 118.3 | 119 | 119.6 |
| Pedregulho-SP | 353700 | 25.1 | 25 | 24.9 | 24.7 | 18.5 | 42.9 | 54.9 | 48.6 | 72.6 | 66.2 | 54 |
| Pedreira-SP | 353710 | 31.5 | 38.1 | 42.3 | 37 | 43.4 | 42.8 | 44.4 | 48.2 | 47.6 | 44.9 | 46.5 |
| Pedrinhas Paulista-SP | 353715 | 66.8 | 99.9 | 99.5 | 99.2 | 98.9 | 65.8 | 32.8 | 130.8 | 97.8 | 65 | 97.2 |
| Pedro de Toledo-SP | 353720 | 68.3 | 67.6 | 57.4 | 66.3 | 65.7 | 83.7 | 82.9 | 54.8 | 72.4 | 80.7 | 80.1 |
| Penápolis-SP | 353730 | 42.3 | 43.7 | 38.3 | 41.4 | 47.7 | 47.4 | 52 | 54.9 | 51.4 | 62.2 | 68.2 |
| Pereira Barreto-SP | 353740 | 39.1 | 43 | 54.7 | 54.7 | 54.7 | 66.4 | 54.6 | 50.7 | 89.7 | 81.9 | 85.7 |
| Pereiras-SP | 353750 | 67.7 | 66.5 | 65.4 | 77.3 | 88.9 | 100.1 | 98.7 | 85.1 | 72 | 94.7 | 105.1 |
| Peruíbe-SP | 353760 | 35.3 | 39.7 | 45.7 | 41.9 | 42.9 | 58.1 | 57.4 | 55.2 | 51.5 | 47.9 | 63.7 |
| Piacatu-SP | 353770 | 56.8 | 56.1 | 55.4 | 91.2 | 90.1 | 107 | 105.7 | 104.5 | 103.4 | 102.4 | 101.3 |
| Piedade-SP | 353780 | 17 | 24.4 | 22.4 | 24.2 | 29.7 | 29.6 | 33.1 | 38.5 | 40.2 | 41.9 | 45.3 |
| Pilar do Sul-SP | 353790 | 22.6 | 33.6 | 44.3 | 43.9 | 43.6 | 50.4 | 46.4 | 53.1 | 42.1 | 55.7 | 55.2 |
| Pindamonhangaba-SP | 353800 | 34.8 | 35 | 38.5 | 40 | 42 | 42.8 | 45.4 | 51.6 | 52.9 | 56.5 | 60.7 |
| Pindorama-SP | 353810 | 13.3 | 13.1 | 25.9 | 25.6 | 63.3 | 43.8 | 43.3 | 48.9 | 48.4 | 47.9 | 47.4 |
| Pinhalzinho-SP | 353820 | 7.7 | 7.6 | 7.4 | 7.3 | 21.7 | 21.4 | 28.1 | 27.7 | 27.3 | 33.7 | 39.9 |
| Piquerobi-SP | 353830 | 55.4 | 55.3 | 27.6 | 27.5 | 27.5 | 27.4 | 27.4 | 27.3 | 81.7 | 81.6 | 81.4 |
| Piquete-SP | 353850 | 20.5 | 34.3 | 34.6 | 55.7 | 70 | 56.4 | 49.7 | 71.4 | 79.1 | 94 | 65.5 |
| Piracaia-SP | 353860 | 11.8 | 11.7 | 23.3 | 23.1 | 23 | 15.2 | 22.7 | 22.5 | 37.3 | 33.4 | 33.2 |
| Piracicaba-SP | 353870 | 45 | 49.2 | 50.8 | 51.7 | 73.6 | 74.5 | 108.1 | 126.1 | 137.2 | 136.8 | 128.7 |
| Piraju-SP | 353880 | 58.5 | 72.1 | 71.9 | 71.8 | 75 | 81.6 | 81.4 | 94.8 | 91.2 | 91 | 100.9 |
| Pirajuí-SP | 353890 | 48.4 | 47.8 | 47.3 | 63.8 | 63.1 | 66.6 | 74.1 | 85.6 | 104.9 | 99.9 | 102.9 |
| Pirangi-SP | 353900 | 37.2 | 36.9 | 45.9 | 45.7 | 45.4 | 63.2 | 35.9 | 35.7 | 44.4 | 53.1 | 44 |
| Pirapora do Bom Jesus-SP | 353910 | 45.3 | 44.3 | 37.2 | 42.6 | 35.8 | 52.7 | 80.5 | 90.4 | 88.8 | 76.5 | 64.5 |
| Pirapozinho-SP | 353920 | 60.6 | 67.9 | 71.1 | 62.6 | 69.7 | 80.6 | 72.2 | 60.2 | 63.4 | 66.5 | 76.9 |
| Pirassununga-SP | 353930 | 48.1 | 67.3 | 68.2 | 65 | 79.6 | 83.1 | 89.3 | 108.8 | 106.7 | 107.4 | 106.7 |
| Piratininga-SP | 353940 | 41.4 | 40.9 | 64.6 | 79.9 | 110.6 | 109.3 | 100.4 | 99.3 | 105.8 | 104.7 | 111.1 |
| Pitangueiras-SP | 353950 | 56.6 | 61.5 | 58 | 60.1 | 62.2 | 66.9 | 66.2 | 81.2 | 80.3 | 77 | 81.3 |
| Planalto-SP | 353960 | 45.3 | 66.8 | 87.4 | 86 | 84.7 | 83.3 | 82.1 | 80.8 | 99.5 | 137.4 | 135.5 |
| Platina-SP | 353970 | 62.4 | 92.6 | 61.1 | 60.6 | 60 | 59.4 | 58.9 | 87.5 | 86.7 | 85.9 | 113.6 |
| Poá-SP | 353980 | 21.6 | 19.5 | 28.5 | 28.3 | 28 | 26 | 33.7 | 29.9 | 33.1 | 33.7 | 32.6 |
| Poloni-SP | 353990 | 18.5 | 18.3 | 18.1 | 17.9 | 17.7 | 17.5 | 52 | 51.4 | 50.9 | 50.4 | 50 |
| Pompéia-SP | 354000 | 64.7 | 64.1 | 68.4 | 87.2 | 91.3 | 85.7 | 89.8 | 84.3 | 102.2 | 101.5 | 91.5 |
| Pongaí-SP | 354010 | 27.7 | 55.8 | 28 | 56.3 | 56.6 | 56.9 | 57.2 | 57.5 | 57.7 | 58 | 58.3 |
| Pontal-SP | 354020 | 38.3 | 37.3 | 26.7 | 23.7 | 30.1 | 33.9 | 39.8 | 47.6 | 40.3 | 37.4 | 42.8 |
| Pontalinda-SP | 354025 | 49.2 | 48.5 | 47.9 | 47.3 | 70.1 | 46.2 | 45.7 | 45.1 | 66.9 | 66.2 | 65.5 |
| Pontes Gestal-SP | 354030 | 38.7 | 38.7 | 38.7 | 38.7 | 77.5 | 77.5 | 77.5 | 38.8 | 77.6 | 116.4 | 38.8 |
| Populina-SP | 354040 | 137.4 | 184 | 184.8 | 208.8 | 163.1 | 187.1 | 188 | 165.2 | 142.2 | 119 | 119.4 |
| Porangaba-SP | 354050 | 36.6 | 59.8 | 70.3 | 80.5 | 79.1 | 99.9 | 87.3 | 107.2 | 105.5 | 103.8 | 102.3 |
| Porto Feliz-SP | 354060 | 42.5 | 40.2 | 37.9 | 53.5 | 57.1 | 56.7 | 58.3 | 61.8 | 63.3 | 57.2 | 56.8 |
| Porto Ferreira-SP | 354070 | 52.1 | 55.5 | 64.5 | 73.5 | 71.1 | 66.8 | 64.5 | 58.5 | 56.3 | 52.3 | 64.5 |
| Potim-SP | 354075 | 32.1 | 46.6 | 30.2 | 29.4 | 42.9 | 41.9 | 45.4 | 31 | 38.9 | 38.1 | 41.4 |
| Potirendaba-SP | 354080 | 45.3 | 44.7 | 50.5 | 31.2 | 37.1 | 36.7 | 66.6 | 77.8 | 71.1 | 76.3 | 98.8 |
| Pracinha-SP | 354085 | 113.4 | 107.6 | 102.4 | 98 | 93.9 | 90.2 | 86.8 | 111.4 | 134.4 | 130 | 125.9 |
| Pradópolis-SP | 354090 | 17.7 | 17.3 | 22.5 | 16.5 | 21.4 | 42 | 30.8 | 15.1 | 29.5 | 29 | 47.4 |
| Praia Grande-SP | 354100 | 32.2 | 35.1 | 30.5 | 32 | 35.5 | 36.1 | 61.2 | 62.5 | 72.3 | 77 | 80.2 |
| Pratânia-SP | 354105 | 43.7 | 43 | 21.2 | 83.7 | 103.3 | 81.6 | 60.5 | 39.8 | 59 | 77.7 | 57.6 |
| Presidente Alves-SP | 354110 | 0 | 0 | 23.7 | 23.7 | 23.8 | 0 | 24 | 24.1 | 48.4 | 24.3 | 48.7 |
| Presidente Bernardes-SP | 354120 | 85.1 | 92.8 | 93.5 | 101.3 | 94.7 | 95.3 | 110.7 | 89.2 | 89.8 | 97.9 | 98.6 |
| Presidente Epitácio-SP | 354130 | 26.3 | 30.9 | 26 | 4.7 | 9.4 | 14 | 34.7 | 34.6 | 34.4 | 36.5 | 34.1 |
| Presidente Prudente-SP | 354140 | 74.2 | 74.9 | 96.3 | 123 | 132.1 | 130.6 | 132.3 | 119.4 | 154.3 | 161.1 | 187.6 |
| Presidente Venceslau-SP | 354150 | 43.9 | 54.1 | 61.8 | 64.2 | 61.5 | 71.6 | 89.4 | 81.6 | 91.6 | 99 | 106.5 |
| Promissão-SP | 354160 | 53.3 | 49.9 | 57.4 | 110.8 | 120.2 | 113.5 | 135.7 | 136.7 | 132.7 | 128.8 | 114.9 |
| Quadra-SP | 354165 | 0 | 30.7 | 30.1 | 29.7 | 58.4 | 57.4 | 56.5 | 55.6 | 82.2 | 81.1 | 79.9 |
| Quatá-SP | 354170 | 54.4 | 46.2 | 68.6 | 75.6 | 82.4 | 74.3 | 73.7 | 73.1 | 87 | 86.3 | 92.8 |
| Queiroz-SP | 354180 | 36.4 | 35.5 | 34.7 | 34 | 33.3 | 65.4 | 96.2 | 125.8 | 216.2 | 212.5 | 179.1 |
| Queluz-SP | 354190 | 53.8 | 70.4 | 69 | 67.8 | 58.3 | 65.5 | 88.6 | 71.3 | 77.9 | 61.4 | 75.6 |
| Quintana-SP | 354200 | 49.7 | 49.2 | 48.7 | 64.4 | 63.8 | 79.1 | 94.1 | 93.3 | 92.5 | 91.8 | 91.1 |
| Rafard-SP | 354210 | 22.8 | 22.7 | 22.7 | 33.9 | 33.8 | 22.4 | 22.4 | 44.6 | 44.5 | 66.5 | 33.1 |
| Rancharia-SP | 354220 | 74.6 | 84.7 | 84.7 | 101.5 | 91.3 | 101.4 | 118.2 | 135 | 138.3 | 128.1 | 121.3 |
| Redenção da Serra-SP | 354230 | 100 | 75.3 | 100.8 | 101.1 | 101.4 | 101.8 | 102.1 | 128.1 | 128.6 | 154.8 | 103.5 |
| Regente Feijó-SP | 354240 | 59 | 69.1 | 73.8 | 89 | 88.3 | 67 | 76.7 | 81.2 | 90.7 | 95.1 | 99.4 |
| Reginópolis-SP | 354250 | 71.5 | 82.8 | 66.6 | 64.5 | 62.6 | 72.9 | 82.7 | 114.8 | 123 | 119.9 | 117 |
| Registro-SP | 354260 | 59.5 | 68.4 | 71.9 | 88 | 91.4 | 89.5 | 112.6 | 139.2 | 146.2 | 138.9 | 138.7 |
| Restinga-SP | 354270 | 30.6 | 30.1 | 44.4 | 43.8 | 43.2 | 71 | 84.1 | 55.3 | 68.3 | 67.4 | 66.6 |
| Ribeira-SP | 354280 | 28.8 | 29 | 29.1 | 29.1 | 58.5 | 29.4 | 58.9 | 59.1 | 59.3 | 89.2 | 59.7 |
| Ribeirão Bonito-SP | 354290 | 49 | 56.7 | 64.3 | 71.8 | 79.3 | 94.5 | 86 | 93.1 | 84.8 | 84.3 | 68.5 |
| Ribeirão Branco-SP | 354300 | 36.3 | 36.8 | 42.7 | 54.1 | 49.4 | 50.1 | 50.9 | 51.6 | 64 | 65 | 77.9 |
| Ribeirão Corrente-SP | 354310 | 69.8 | 46.1 | 68.5 | 67.9 | 67.3 | 66.7 | 66.2 | 65.6 | 65.1 | 64.6 | 64.1 |
| Ribeirão do Sul-SP | 354320 | 21.9 | 21.9 | 43.9 | 43.9 | 43.9 | 87.9 | 109.9 | 66 | 88 | 66 | 110.1 |
| Ribeirão dos Índios-SP | 354323 | 44.5 | 44.6 | 44.6 | 44.6 | 44.7 | 44.7 | 44.8 | 44.8 | 44.8 | 89.7 | 44.9 |
| Ribeirão Grande-SP | 354325 | 39.5 | 39.5 | 39.4 | 52.5 | 39.4 | 39.3 | 39.3 | 39.2 | 39.2 | 39.2 | 52.2 |
| Ribeirão Pires-SP | 354330 | 32.4 | 32.1 | 24.1 | 18.8 | 27.1 | 42.1 | 51 | 35.7 | 41.2 | 38.5 | 35.8 |
| Ribeirão Preto-SP | 354340 | 115.4 | 116.1 | 117.9 | 122.2 | 128.7 | 130.4 | 138.7 | 143.9 | 154.6 | 156.1 | 163.4 |
| Riversul-SP | 354350 | 46.1 | 46.8 | 79.1 | 48.2 | 48.9 | 33.1 | 50.3 | 34.1 | 34.6 | 52.7 | 53.5 |
| Rifaina-SP | 354360 | 57.2 | 28.5 | 56.8 | 56.6 | 56.4 | 56.2 | 84 | 83.7 | 83.4 | 55.4 | 55.3 |
| Rincão-SP | 354370 | 18.8 | 18.8 | 28.1 | 46.8 | 37.4 | 28 | 74.5 | 55.8 | 55.8 | 55.7 | 46.4 |
| Rinópolis-SP | 354380 | 29.3 | 29.4 | 49.1 | 49.2 | 49.3 | 49.4 | 59.4 | 59.6 | 79.6 | 109.7 | 90 |
| Rio Claro-SP | 354390 | 30.5 | 31.7 | 36.1 | 43.6 | 45.8 | 48.4 | 59.1 | 62.6 | 58.1 | 63 | 77.1 |
| Rio das Pedras-SP | 354400 | 24.1 | 27 | 26.5 | 32.5 | 28.7 | 34.4 | 30.8 | 30.2 | 32.7 | 26.3 | 46.1 |
| Rio Grande da Serra-SP | 354410 | 22.9 | 22.5 | 17.7 | 26.2 | 23.7 | 27.6 | 27.3 | 39.3 | 36.7 | 36.3 | 47.8 |
| Riolândia-SP | 354420 | 28.8 | 28.2 | 46.1 | 63.5 | 89.1 | 78.9 | 86.2 | 118.7 | 125.2 | 139.8 | 137.7 |
| Rosana-SP | 354425 | 85.5 | 97 | 113.9 | 111.2 | 129 | 147.5 | 166.8 | 181.6 | 185.6 | 206.9 | 211.6 |
| Roseira-SP | 354430 | 31.2 | 30.8 | 30.5 | 30.2 | 69.7 | 78.9 | 68.4 | 87.1 | 95.8 | 95 | 113 |
| Rubiácea-SP | 354440 | 73.6 | 72.5 | 71.5 | 105.8 | 104.4 | 137.5 | 101.8 | 100.5 | 99.3 | 130.8 | 161.7 |
| Rubinéia-SP | 354450 | 34.7 | 34.4 | 68.2 | 101.4 | 134.1 | 133 | 132 | 130.9 | 129.9 | 128.9 | 128 |
| Sabino-SP | 354460 | 18.9 | 18.8 | 18.7 | 37.2 | 37 | 36.8 | 36.7 | 36.5 | 54.4 | 36.1 | 53.9 |
| Sagres-SP | 354470 | 40.6 | 40.7 | 40.7 | 81.6 | 81.6 | 81.7 | 122.7 | 122.9 | 123 | 123.1 | 123.2 |
| Sales-SP | 354480 | 18.5 | 18.2 | 17.9 | 35.3 | 52.1 | 85.6 | 84.4 | 83.2 | 98.5 | 113.4 | 111.9 |
| Sales Oliveira-SP | 354490 | 47.3 | 46.7 | 64.6 | 73 | 63.2 | 62.5 | 79.6 | 70 | 77.9 | 68.6 | 59.4 |
| Salesópolis-SP | 354500 | 50.8 | 56.6 | 37.4 | 37.1 | 43 | 36.6 | 36.3 | 48 | 53.6 | 53.2 | 47 |
| Salmourão-SP | 354510 | 41.2 | 40.9 | 40.5 | 20.1 | 19.9 | 79 | 117.6 | 97.2 | 96.5 | 95.7 | 95 |
| Saltinho-SP | 354515 | 14.3 | 14.1 | 27.6 | 54.4 | 53.5 | 52.7 | 64.8 | 51.1 | 50.3 | 62 | 61.2 |
| Salto-SP | 354520 | 21.8 | 20.6 | 19.4 | 16.5 | 30.8 | 33.1 | 38.1 | 48.2 | 47.7 | 53.2 | 51 |
| Salto de Pirapora-SP | 354530 | 89.8 | 78.8 | 65.6 | 74.5 | 78.4 | 84.5 | 85.9 | 98.7 | 115.8 | 89.9 | 111.2 |
| Salto Grande-SP | 354540 | 44.8 | 55.7 | 55.5 | 55.3 | 66.1 | 76.8 | 87.4 | 97.9 | 75.9 | 97.2 | 86 |
| Sandovalina-SP | 354550 | 81.8 | 80.4 | 79.1 | 77.9 | 76.8 | 75.7 | 74.6 | 73.5 | 72.5 | 71.5 | 94.2 |
| Santa Adélia-SP | 354560 | 48.3 | 34.3 | 40.8 | 33.8 | 47.1 | 53.5 | 53.2 | 72.7 | 72.2 | 71.8 | 84.4 |
| Santa Albertina-SP | 354570 | 51.4 | 34.2 | 51.1 | 68 | 84.8 | 67.6 | 84.3 | 117.7 | 117.4 | 100.4 | 116.8 |
| Santa Bárbara d'Oeste-SP | 354580 | 12.1 | 12 | 17.9 | 19.4 | 23 | 28.3 | 26 | 25.8 | 26.8 | 25.6 | 30.1 |
| Santa Branca-SP | 354600 | 71.7 | 35.6 | 35.4 | 63.4 | 77.1 | 90.7 | 90.2 | 82.8 | 89.2 | 75.1 | 61.2 |
| Santa Clara d'Oeste-SP | 354610 | 46.7 | 46.8 | 46.8 | 46.9 | 93.9 | 93.9 | 94 | 94.2 | 94.3 | 94.3 | 94.4 |
| Santa Cruz da Conceição-SP | 354620 | 49.9 | 74 | 73.1 | 24.1 | 23.8 | 23.6 | 23.3 | 23.1 | 22.9 | 22.6 | 89.7 |
| Santa Cruz da Esperança-SP | 354625 | 50.8 | 50.4 | 99.9 | 99.2 | 98.4 | 146.5 | 193.8 | 192.3 | 190.9 | 142.2 | 94.2 |
| Santa Cruz das Palmeiras-SP | 354630 | 20.1 | 29.8 | 32.6 | 32.2 | 41.2 | 40.7 | 37.1 | 42.7 | 45.2 | 44.7 | 47.1 |
| Santa Cruz do Rio Pardo-SP | 354640 | 45.1 | 47 | 51.1 | 50.7 | 52.6 | 69.7 | 97.4 | 90.3 | 102.5 | 101.9 | 103.4 |
| Santa Ernestina-SP | 354650 | 0 | 17.5 | 35 | 52.7 | 52.8 | 17.6 | 88.4 | 124 | 106.5 | 106.7 | 124.8 |
| Santa Fé do Sul-SP | 354660 | 74.8 | 84.2 | 113.4 | 129 | 131.1 | 136.5 | 141.8 | 146.9 | 139.4 | 147.7 | 155.9 |
| Santa Gertrudes-SP | 354670 | 42.8 | 60.2 | 58.6 | 57.2 | 64.5 | 71.5 | 69.9 | 68.4 | 59.1 | 65.6 | 60.6 |
| Santa Isabel-SP | 354680 | 65.6 | 58.8 | 54.1 | 53.5 | 58.5 | 70.8 | 82.9 | 54.6 | 73.8 | 83.7 | 84.5 |
| Santa Lúcia-SP | 354690 | 47.8 | 47.6 | 47.3 | 35.3 | 46.9 | 46.6 | 69.6 | 46.2 | 57.5 | 57.2 | 79.7 |
| Santa Maria da Serra-SP | 354700 | 55.6 | 54.8 | 54.1 | 124.6 | 123 | 104.1 | 85.7 | 101.6 | 100.5 | 82.8 | 81.9 |
| Santa Mercedes-SP | 354710 | 69.1 | 69 | 103.4 | 103.2 | 103.1 | 102.9 | 102.8 | 102.6 | 102.5 | 136.5 | 102.2 |
| Santana da Ponte Pensa-SP | 354720 | 57.8 | 58.6 | 59.5 | 60.2 | 122.1 | 185.6 | 188.2 | 191 | 258.2 | 261.8 | 199.1 |
| Santana de Parnaíba-SP | 354730 | 30.6 | 25.9 | 34.1 | 42.7 | 51.8 | 52.1 | 35.4 | 47.9 | 53.6 | 50.2 | 64.5 |
| Santa Rita d'Oeste-SP | 354740 | 75.9 | 76.3 | 76.7 | 38.5 | 38.7 | 38.9 | 39.1 | 39.3 | 39.5 | 79.3 | 79.7 |
| Santa Rita do Passa Quatr-SP | 354750 | 74 | 73.8 | 81.1 | 110.3 | 121.2 | 131.9 | 131.7 | 135.1 | 124 | 131.1 | 134.5 |
| Santa Rosa de Viterbo-SP | 354760 | 29.2 | 28.9 | 36.8 | 44.5 | 44.1 | 51.7 | 55.1 | 54.6 | 54.1 | 53.6 | 60.8 |
| Santa Salete-SP | 354765 | 68.1 | 67.8 | 67.4 | 201.3 | 267.2 | 199.5 | 198.5 | 197.6 | 196.7 | 195.8 | 194.9 |
| Santo Anastácio-SP | 354770 | 33.3 | 33.3 | 52.4 | 52.4 | 62 | 62 | 66.9 | 76.5 | 66.9 | 71.8 | 76.6 |
| Santo André-SP | 354780 | 40.9 | 49.1 | 53.1 | 54 | 53.8 | 57.4 | 66 | 69.2 | 72.8 | 74.6 | 74 |
| Santo Antônio da Alegria-SP | 354790 | 78.8 | 78.1 | 61.9 | 92.1 | 106.6 | 105.7 | 89.9 | 89.2 | 103.2 | 131.8 | 116.3 |
| Santo Antônio de Posse-SP | 354800 | 29.1 | 38.3 | 61.4 | 65.4 | 64.7 | 59.4 | 49.7 | 40.2 | 48.6 | 39.4 | 65 |
| Santo Antônio do Aracangu-SP | 354805 | 91.3 | 64.6 | 89.5 | 101.4 | 125.7 | 112.2 | 111.2 | 110.3 | 121.6 | 96.5 | 95.7 |
| Santo Antônio do Jardim-SP | 354810 | 32.6 | 32.7 | 49.2 | 49.4 | 49.5 | 33.1 | 49.8 | 49.9 | 50 | 50.1 | 50.3 |
| Santo Antônio do Pinhal-SP | 354820 | 45.4 | 45.3 | 60.2 | 60 | 44.9 | 59.7 | 59.5 | 59.3 | 74 | 73.8 | 88.3 |
| Santo Expedito-SP | 354830 | 71 | 70.3 | 34.8 | 69 | 68.3 | 33.9 | 100.6 | 99.7 | 65.9 | 65.4 | 64.8 |
| Santópolis do Aguapeí-SP | 354840 | 70 | 69.2 | 114.1 | 112.9 | 111.8 | 132.8 | 131.5 | 151.9 | 172 | 170.4 | 168.9 |
| Santos-SP | 354850 | 70.6 | 76.6 | 82.3 | 83.2 | 97.5 | 107.2 | 114.3 | 120.2 | 131.9 | 153.7 | 169.3 |
| São Bento do Sapucaí-SP | 354860 | 46.8 | 46.7 | 55.9 | 46.5 | 55.7 | 64.9 | 64.8 | 55.5 | 64.6 | 64.5 | 82.9 |
| São Bernardo do Campo-SP | 354870 | 38.2 | 36.8 | 44.2 | 53.1 | 57.3 | 61.6 | 76.7 | 87.7 | 85.4 | 89.3 | 95.8 |
| São Caetano do Sul-SP | 354880 | 67.5 | 75.6 | 74.5 | 90.3 | 90.4 | 95 | 102.8 | 97.1 | 86.4 | 91 | 116.1 |
| São Carlos-SP | 354890 | 37.5 | 39.6 | 41.3 | 43.9 | 46.3 | 46.6 | 50.3 | 50.5 | 90.5 | 89.6 | 83.8 |
| São Francisco-SP | 354900 | 34.8 | 34.9 | 34.9 | 35 | 70.1 | 70.2 | 70.3 | 70.4 | 70.5 | 70.7 | 70.8 |
| São João da Boa Vista-SP | 354910 | 33.2 | 34.1 | 47.8 | 60.2 | 85.1 | 85.6 | 85 | 86.7 | 81.6 | 93.3 | 94.9 |
| São João das Duas Pontes-SP | 354920 | 37.8 | 37.9 | 38 | 38.1 | 76.5 | 76.7 | 76.9 | 77.1 | 77.3 | 116.2 | 155.4 |
| São João de Iracema-SP | 354925 | 166.6 | 165.5 | 164.4 | 163.4 | 162.4 | 107.6 | 160.5 | 106.4 | 158.6 | 157.8 | 156.9 |
| São João do Pau d'Alho-SP | 354930 | 46.1 | 46.3 | 46.4 | 46.5 | 46.6 | 46.8 | 46.9 | 47 | 47.1 | 47.3 | 47.4 |
| São Joaquim da Barra-SP | 354940 | 34.3 | 36 | 33.6 | 54 | 61.7 | 69.2 | 64.5 | 67.9 | 67.3 | 68.6 | 71.9 |
| São José da Bela Vista-SP | 354950 | 35.1 | 35 | 46.4 | 46.2 | 80.6 | 103.2 | 91.3 | 91 | 90.6 | 67.7 | 56.2 |
| São José do Barreiro-SP | 354960 | 47.8 | 95.6 | 71.8 | 71.9 | 71.9 | 144 | 144.1 | 144.2 | 144.3 | 120.4 | 144.5 |
| São José do Rio Pardo-SP | 354970 | 43.6 | 45.3 | 54.5 | 50.6 | 46.6 | 55.8 | 64.8 | 66.4 | 64.4 | 64.1 | 71.2 |
| São José do Rio Preto-SP | 354980 | 101.4 | 102.6 | 120.7 | 123.6 | 134.6 | 141.9 | 156.1 | 166.3 | 171.7 | 191.1 | 196.8 |
| São José dos Campos-SP | 354990 | 48 | 51.2 | 54.2 | 52.1 | 64.7 | 72.7 | 81.6 | 91.1 | 94.8 | 90.3 | 100.6 |
| São Lourenço da Serra-SP | 354995 | 43 | 56.6 | 62.8 | 48.3 | 47.7 | 53.9 | 66.7 | 59.3 | 65.2 | 64.5 | 76.6 |
| São Luís do Paraitinga-SP | 355000 | 56.3 | 56.3 | 46.9 | 56.3 | 56.3 | 75 | 65.6 | 74.9 | 74.9 | 74.9 | 84.2 |
| São Manuel-SP | 355010 | 46.3 | 46 | 45.8 | 40.5 | 55.4 | 55.2 | 52.4 | 47.2 | 49.5 | 54.2 | 56.4 |
| São Miguel Arcanjo-SP | 355020 | 12.5 | 28 | 31 | 30.9 | 30.9 | 30.8 | 73.7 | 95 | 97.8 | 100.7 | 109.6 |
| São Paulo-SP | 355030 | 65.3 | 70.9 | 77.8 | 84.8 | 92.9 | 100.8 | 108.2 | 108.5 | 112.9 | 114.3 | 114.8 |
| São Pedro-SP | 355040 | 22.1 | 12.5 | 15.4 | 15.2 | 33.1 | 26.8 | 32.4 | 52.5 | 66.4 | 74.3 | 99.1 |
| São Pedro do Turvo-SP | 355050 | 68.4 | 54.5 | 40.7 | 40.5 | 40.3 | 53.5 | 40 | 66.3 | 66 | 65.7 | 65.5 |
| São Roque-SP | 355060 | 20.4 | 23.9 | 22.3 | 22 | 22.9 | 23.7 | 42.1 | 42.7 | 41 | 45 | 38.9 |
| São Sebastião-SP | 355070 | 77.2 | 71.4 | 76.5 | 95.7 | 101.5 | 100.9 | 91.7 | 94.8 | 99.1 | 94 | 103.9 |
| São Sebastião da Grama-SP | 355080 | 24.1 | 32.2 | 32.3 | 40.4 | 64.8 | 64.9 | 81.3 | 89.6 | 89.8 | 90 | 90.1 |
| São Simão-SP | 355090 | 27.5 | 41 | 34 | 40.6 | 53.9 | 67 | 53.4 | 53.1 | 39.7 | 46.1 | 52.4 |
| São Vicente-SP | 355100 | 29.6 | 28.7 | 28.8 | 32.9 | 35.2 | 39.5 | 42.9 | 46.5 | 41.4 | 40.2 | 36.6 |
| Sarapuí-SP | 355110 | 11.1 | 11 | 10.8 | 53.4 | 42.2 | 41.6 | 41.1 | 40.7 | 70.3 | 39.7 | 39.3 |
| Sarutaiá-SP | 355120 | 26.8 | 26.9 | 53.9 | 81 | 54.1 | 54.2 | 81.5 | 27.2 | 81.9 | 82.1 | 82.3 |
| Sebastianópolis do Sul-SP | 355130 | 33.2 | 65.4 | 96.6 | 63.4 | 125 | 154.1 | 151.9 | 149.8 | 147.8 | 145.9 | 115.3 |
| Serra Azul-SP | 355140 | 18.6 | 53.8 | 52 | 67.3 | 65.3 | 63.5 | 61.8 | 67.6 | 80.5 | 78.6 | 83.7 |
| Serrana-SP | 355150 | 23.3 | 25.5 | 27.6 | 34.6 | 36.5 | 40.8 | 47.4 | 58.4 | 53 | 47.7 | 38.2 |
| Serra Negra-SP | 355160 | 37.7 | 41.1 | 40.7 | 65.9 | 54.5 | 57.6 | 64.2 | 56.6 | 59.6 | 55.6 | 55.2 |
| Sertãozinho-SP | 355170 | 29.2 | 27.9 | 33.7 | 35.9 | 37.1 | 36.7 | 40.4 | 41.6 | 57.5 | 60.1 | 65.9 |
| Sete Barras-SP | 355180 | 29.7 | 37.3 | 45 | 45.2 | 60.5 | 76 | 53.4 | 61.3 | 84.7 | 85 | 54.3 |
| Severínia-SP | 355190 | 58.1 | 57.3 | 56.6 | 62.2 | 67.7 | 79.1 | 84.3 | 71.4 | 58.9 | 58.3 | 63.5 |
| Silveiras-SP | 355200 | 17.1 | 33.9 | 50.5 | 66.9 | 49.8 | 49.5 | 65.5 | 65.1 | 64.7 | 112.4 | 95.8 |
| Socorro-SP | 355210 | 24.5 | 26.9 | 26.6 | 26.3 | 26.1 | 25.8 | 35.8 | 32.9 | 55.1 | 64.5 | 61.5 |
| Sorocaba-SP | 355220 | 55.5 | 56.2 | 60.3 | 65.9 | 69.8 | 72.8 | 77.3 | 87.9 | 93.5 | 86.1 | 89.2 |
| Sud Mennucci-SP | 355230 | 92.1 | 105.1 | 118.1 | 104.8 | 91.6 | 117.6 | 130.4 | 130.3 | 130.1 | 142.9 | 155.7 |
| Sumaré-SP | 355240 | 38.5 | 39 | 44.8 | 48 | 57.1 | 61.6 | 64.1 | 68.3 | 74.3 | 67.7 | 70 |
| Suzano-SP | 355250 | 28.2 | 30.1 | 32.3 | 30.1 | 43.9 | 61.3 | 58.5 | 42.1 | 39.2 | 39.1 | 48.9 |
| Suzanápolis-SP | 355255 | 29.9 | 29.3 | 57.7 | 56.8 | 55.9 | 55 | 81.3 | 80 | 52.6 | 103.7 | 102.3 |
| Tabapuã-SP | 355260 | 26.2 | 17.3 | 25.8 | 25.6 | 25.4 | 25.2 | 33.4 | 41.4 | 49.3 | 49 | 48.7 |
| Tabatinga-SP | 355270 | 47.6 | 47 | 66.4 | 59.1 | 58.5 | 70.8 | 70 | 81.9 | 81.1 | 74.1 | 79.5 |
| Taboão da Serra-SP | 355280 | 45.2 | 48 | 53.9 | 61.6 | 64.4 | 60.6 | 97.3 | 86.1 | 93.8 | 94.5 | 92.4 |
| Taciba-SP | 355290 | 69.5 | 68.9 | 68.3 | 67.7 | 100.8 | 83.3 | 66.1 | 65.6 | 65.1 | 64.6 | 64.1 |
| Taguaí-SP | 355300 | 19.2 | 27.9 | 36 | 43.8 | 51.2 | 58.2 | 72.9 | 86.8 | 84.8 | 98 | 95.8 |
| Taiaçu-SP | 355310 | 100.3 | 99.8 | 99.3 | 98.8 | 114.8 | 97.9 | 81.2 | 64.7 | 64.4 | 80.1 | 95.7 |
| Taiúva-SP | 355320 | 35.8 | 35.8 | 71.6 | 71.7 | 89.6 | 89.6 | 107.6 | 107.6 | 125.6 | 125.7 | 143.7 |
| Tambaú-SP | 355330 | 43.6 | 61 | 65.3 | 47.8 | 47.8 | 47.7 | 52 | 56.3 | 73.5 | 73.4 | 69 |
| Tanabi-SP | 355340 | 45.2 | 49 | 48.7 | 28.2 | 24 | 27.9 | 27.7 | 27.6 | 23.5 | 31.1 | 38.7 |
| Tapiraí-SP | 355350 | 24.1 | 24.2 | 24.4 | 61.2 | 86.2 | 61.9 | 74.7 | 75.2 | 63 | 63.3 | 89.2 |
| Tapiratiba-SP | 355360 | 38.2 | 30.6 | 38.3 | 30.7 | 30.7 | 46.1 | 69.2 | 69.2 | 84.7 | 84.7 | 92.5 |
| Taquaral-SP | 355365 | 179.2 | 143.3 | 250.5 | 250.4 | 214.4 | 178.6 | 214.1 | 142.7 | 106.9 | 142.5 | 142.3 |
| Taquaritinga-SP | 355370 | 23.7 | 36.3 | 41.6 | 48.6 | 52 | 50 | 71.2 | 70.9 | 58.3 | 58.1 | 61.4 |
| Taquarituba-SP | 355380 | 35.1 | 35.1 | 35 | 43.7 | 39.2 | 60.9 | 69.5 | 73.7 | 69.3 | 69.1 | 77.7 |
| Taquarivaí-SP | 355385 | 58.4 | 57.6 | 75.8 | 93.5 | 73.9 | 73.1 | 72.2 | 71.4 | 141.1 | 139.6 | 86.3 |
| Tarabai-SP | 355390 | 60.6 | 74.8 | 88.6 | 102.2 | 86.6 | 57.1 | 84.7 | 69.8 | 55.2 | 68.3 | 81.1 |
| Tarumã-SP | 355395 | 78.3 | 61.6 | 68.1 | 82 | 80.8 | 79.6 | 78.5 | 63.3 | 69.3 | 68.4 | 81 |
| Tatuí-SP | 355400 | 42 | 50.6 | 50 | 53 | 59.5 | 65.8 | 69.4 | 65.1 | 66.1 | 63.7 | 68.9 |
| Taubaté-SP | 355410 | 51.7 | 56.4 | 60.6 | 74.4 | 81.8 | 72.7 | 72.3 | 84.4 | 98.2 | 121.8 | 132.4 |
| Tejupá-SP | 355420 | 19.9 | 20.1 | 40.6 | 20.5 | 20.7 | 62.6 | 84.2 | 106.3 | 85.9 | 65 | 65.6 |
| Teodoro Sampaio-SP | 355430 | 60.1 | 68.9 | 68.4 | 27.2 | 45 | 49.2 | 62.3 | 70.7 | 87.9 | 96.1 | 82.5 |
| Terra Roxa-SP | 355440 | 70.1 | 81 | 68.8 | 79.6 | 78.9 | 78.3 | 55.5 | 66 | 65.5 | 65 | 86 |
| Tietê-SP | 355450 | 16.3 | 18.8 | 15.9 | 15.7 | 33.6 | 40.8 | 32.7 | 37.3 | 44.2 | 68 | 69.7 |
| Timburi-SP | 355460 | 36.7 | 73.6 | 73.7 | 73.9 | 111.1 | 37.1 | 74.4 | 111.9 | 112.1 | 74.9 | 75.1 |
| Torre de Pedra-SP | 355465 | 43.7 | 43.5 | 43.3 | 0 | 42.8 | 42.6 | 84.9 | 84.5 | 84 | 83.6 | 83.3 |
| Torrinha-SP | 355470 | 21.2 | 21 | 20.9 | 31.2 | 51.7 | 41.2 | 51.2 | 61.1 | 81.1 | 80.7 | 100.4 |
| Trabiju-SP | 355475 | 129.1 | 127.7 | 63.2 | 62.5 | 123.8 | 122.6 | 121.4 | 120.3 | 119.1 | 59 | 175.5 |
| Tremembé-SP | 355480 | 44.2 | 36.2 | 42.8 | 56.3 | 50.9 | 52.5 | 60.9 | 62.3 | 54.9 | 56.4 | 60 |
| Três Fronteiras-SP | 355490 | 54.5 | 54.2 | 53.9 | 53.7 | 53.4 | 53.1 | 52.9 | 70.2 | 69.8 | 52.1 | 69.2 |
| Tuiuti-SP | 355495 | 17 | 16.7 | 16.5 | 16.2 | 16 | 15.7 | 31 | 30.6 | 15.1 | 29.8 | 29.4 |
| Tupã-SP | 355500 | 72.4 | 78.4 | 92.2 | 81.4 | 92.1 | 95.1 | 105.7 | 105.6 | 105.5 | 131.4 | 139 |
| Tupi Paulista-SP | 355510 | 55.5 | 96.4 | 109.4 | 115.4 | 107.9 | 113.9 | 139.8 | 138.9 | 111.7 | 130.6 | 136.3 |
| Turiúba-SP | 355520 | 101.6 | 101.4 | 101.1 | 100.9 | 100.7 | 100.5 | 100.3 | 100 | 99.8 | 99.6 | 99.4 |
| Turmalina-SP | 355530 | 47.6 | 48.4 | 49.3 | 50.2 | 51 | 103.8 | 105.7 | 107.7 | 109.6 | 111.7 | 113.7 |
| Ubarana-SP | 355535 | 38.4 | 56.5 | 55.3 | 54.3 | 53.4 | 52.4 | 51.5 | 50.6 | 49.8 | 49 | 48.3 |
| Ubatuba-SP | 355540 | 52.3 | 57.8 | 65.6 | 63.5 | 55.4 | 65.3 | 70.3 | 94.8 | 100.5 | 99.2 | 107 |
| Ubirajara-SP | 355550 | 111.6 | 110.9 | 132.2 | 109.5 | 130.6 | 129.9 | 150.6 | 171.1 | 148.9 | 148.1 | 168.3 |
| Uchoa-SP | 355560 | 62.4 | 62.1 | 82.4 | 123 | 112.2 | 91.4 | 111.2 | 80.5 | 50.1 | 89.8 | 99.3 |
| União Paulista-SP | 355570 | 62.9 | 62 | 61 | 120.3 | 177.9 | 175.5 | 173.2 | 170.8 | 168.6 | 166.6 | 164.6 |
| Urânia-SP | 355580 | 22.1 | 33.1 | 44.2 | 66.2 | 77.2 | 77.1 | 66.1 | 99 | 109.9 | 131.8 | 131.8 |
| Uru-SP | 355590 | 76.3 | 77.2 | 156 | 157.6 | 159.2 | 161 | 162.7 | 164.5 | 166.3 | 168.1 | 169.9 |
| Urupês-SP | 355600 | 23.4 | 54.1 | 38.4 | 30.5 | 30.3 | 45.1 | 44.8 | 44.5 | 51.6 | 58.6 | 58.3 |
| Valentim Gentil-SP | 355610 | 27.7 | 27.1 | 44.2 | 43.3 | 59.5 | 58.3 | 49.1 | 64.2 | 78.9 | 85.3 | 61 |
| Valinhos-SP | 355620 | 24.8 | 28 | 27.4 | 33.1 | 42.1 | 38.7 | 40.5 | 35.6 | 57.8 | 58.4 | 54.3 |
| Valparaíso-SP | 355630 | 49.3 | 52.8 | 56.2 | 55.3 | 54.4 | 61.8 | 73 | 71.9 | 63 | 97 | 91.8 |
| Vargem-SP | 355635 | 11.6 | 11.3 | 22.2 | 10.9 | 21.4 | 41.9 | 41.2 | 30.4 | 19.9 | 29.4 | 28.9 |
| Vargem Grande do Sul-SP | 355640 | 30.3 | 32.6 | 34.8 | 34.5 | 34.3 | 41.3 | 41 | 50.3 | 57.1 | 49.7 | 51.7 |
| Vargem Grande Paulista-SP | 355645 | 31 | 32.5 | 31.8 | 28.9 | 36.9 | 51.1 | 50.1 | 47 | 52.1 | 53.2 | 52.2 |
| Várzea Paulista-SP | 355650 | 22.5 | 24 | 29.1 | 22.5 | 27.6 | 32.5 | 31.2 | 28.3 | 34.8 | 35.2 | 29.9 |
| Vera Cruz-SP | 355660 | 36.1 | 27.1 | 72.5 | 81.7 | 63.7 | 91.1 | 73.1 | 64.1 | 64.2 | 55.1 | 64.4 |
| Vinhedo-SP | 355670 | 33.9 | 34.6 | 46 | 50.9 | 68.8 | 60.1 | 43.4 | 71.3 | 86 | 84.3 | 87.9 |
| Viradouro-SP | 355680 | 45.9 | 56.9 | 56.4 | 50.4 | 66.7 | 60.7 | 54.8 | 65.2 | 75.6 | 80.4 | 85.2 |
| Vista Alegre do Alto-SP | 355690 | 30.2 | 29.2 | 42.5 | 41.3 | 40.2 | 52.3 | 38.2 | 49.7 | 48.5 | 47.4 | 46.4 |
| Vitória Brasil-SP | 355695 | 56.6 | 56.4 | 56.1 | 55.9 | 55.7 | 55.5 | 55.3 | 165.3 | 164.7 | 164.1 | 163.6 |
| Votorantim-SP | 355700 | 21.2 | 20.9 | 20.7 | 20.4 | 24.6 | 22.6 | 25.8 | 34 | 42.9 | 41.7 | 47.1 |
| Votuporanga-SP | 355710 | 42.4 | 61.7 | 69.1 | 78.7 | 172.7 | 181.1 | 172.7 | 172.1 | 252 | 264.8 | 263.5 |
| Zacarias-SP | 355715 | 129.6 | 127.4 | 167.1 | 164.5 | 121.6 | 119.8 | 157.4 | 155.2 | 153 | 151 | 149 |
| Chavantes-SP | 355720 | 32.2 | 40.3 | 40.3 | 40.3 | 40.3 | 40.3 | 40.3 | 56.4 | 64.4 | 56.4 | 48.3 |
| Estiva Gerbi-SP | 355730 | 39.8 | 19.7 | 38.9 | 48 | 57 | 75.2 | 74.4 | 73.6 | 72.9 | 72.1 | 62.5 |

Source: PROADESS (https://www.proadess.icict.fiocruz.br) and IBGE (<https://www.ibge.gov.br/explica/codigos-dos-municipios.php>)
