# Supplementary material for: Development and validation of an epidemiological risk score for neonatal death in a middle-income country
Source: Front Public Health. 2025 Nov 19;13:1675040. doi: 10.3389/fpubh.2025.1675040 (PMC12672502; doi:10.3389/fpubh.2025.1675040)
Supplement: Supplementary file 13 [file Table_13.docx]

**Parte superior do formulário**

**Parte inferior do formulário**

**Supplementary Material 13**. Population coverage (%) by private health plans or insurance, State of São Paulo, 2008–2018.

| Municipality | Code | 2008 | 2009 | 2010 | 2011 | 2012 | 2013 | 2014 | 2015 | 2016 | 2017 | 2018 |
| --- | --- | --- | --- | --- | --- | --- | --- | --- | --- | --- | --- | --- |
| Adamantina-SP | 350010 | 17.2 | 16.8 | 17.1 | 17.2 | 17.1 | 18.2 | 18.8 | 18.6 | 18.6 | 17.7 | 17.5 |
| Adolfo-SP | 350020 | 10.3 | 10.1 | 10.7 | 11.2 | 12 | 14.6 | 15.7 | 16.3 | 16.7 | 17.3 | 18 |
| Aguaí-SP | 350030 | 19.3 | 21.3 | 21.5 | 20.3 | 23.4 | 23.9 | 20.5 | 20.3 | 19.5 | 19.3 | 18.4 |
| Águas da Prata-SP | 350040 | 17.5 | 18.2 | 18.7 | 19.9 | 20.2 | 21.2 | 22.3 | 22 | 22 | 20.9 | 20.4 |
| Águas de Lindóia-SP | 350050 | 12.6 | 12.7 | 13.9 | 13.6 | 13.8 | 13.8 | 14.2 | 14.2 | 13.9 | 13.5 | 13.1 |
| Águas de Santa Bárbara-SP | 350055 | 12.5 | 16.8 | 17.2 | 16.5 | 12.5 | 13.3 | 18.8 | 18.5 | 18.3 | 15 | 14.4 |
| Águas de São Pedro-SP | 350060 | 49.4 | 49.2 | 49.5 | 47.6 | 47.2 | 49.1 | 49 | 50.7 | 47.7 | 46.9 | 45.3 |
| Agudos-SP | 350070 | 16.4 | 17 | 18.7 | 20.2 | 21.4 | 23 | 23.6 | 24.6 | 23.1 | 22.3 | 21.8 |
| Alambari-SP | 350075 | 8.8 | 9.5 | 13 | 12.5 | 14.5 | 15.7 | 18.7 | 16.5 | 16.5 | 13 | 12 |
| Alfredo Marcondes-SP | 350080 | 20.4 | 22.3 | 21.8 | 23.8 | 24.3 | 23.7 | 23.9 | 24.2 | 24.3 | 24.4 | 25.2 |
| Altair-SP | 350090 | 6 | 8 | 8.8 | 14.2 | 15.3 | 15.6 | 16.5 | 16.7 | 17.8 | 17 | 16.8 |
| Altinópolis-SP | 350100 | 11.2 | 12.6 | 13.3 | 14.2 | 14.3 | 14.9 | 15.1 | 16.3 | 16.4 | 16.5 | 17.6 |
| Alto Alegre-SP | 350110 | 6 | 6.1 | 6.2 | 11.7 | 11.1 | 12.3 | 12.5 | 14.4 | 10.3 | 9.4 | 8.9 |
| Alumínio-SP | 350115 | 84.7 | 72.8 | 40.7 | 41.5 | 47.8 | 50.1 | 49.3 | 47.4 | 46.6 | 43.3 | 42 |
| Álvares Florence-SP | 350120 | 5.9 | 9 | 10 | 10.7 | 11.6 | 12.7 | 13.7 | 10.5 | 11.8 | 12.4 | 12.9 |
| Álvares Machado-SP | 350130 | 18.3 | 19 | 19 | 19.1 | 19.7 | 19.6 | 20.4 | 19.5 | 19.9 | 20.5 | 20.7 |
| Álvaro de Carvalho-SP | 350140 | 2.2 | 2.2 | 2.4 | 1.8 | 2.2 | 2.5 | 3.8 | 3.5 | 2.5 | 2.1 | 1.8 |
| Alvinlândia-SP | 350150 | 3 | 2.9 | 3.4 | 3.5 | 3.7 | 4.4 | 4.8 | 4.5 | 4.4 | 4.4 | 4.7 |
| Americana-SP | 350160 | 53.6 | 55.5 | 60.3 | 63 | 64.1 | 52.4 | 53.8 | 52.8 | 51.5 | 52 | 51.1 |
| Américo Brasiliense-SP | 350170 | 42.1 | 42.7 | 44.3 | 45.9 | 48.3 | 46 | 43.1 | 42.6 | 39.8 | 39.3 | 37.4 |
| Américo de Campos-SP | 350180 | 8.7 | 12.1 | 18.9 | 22.4 | 24.9 | 23.1 | 24.6 | 24.7 | 23.5 | 23.8 | 20.3 |
| Amparo-SP | 350190 | 40.4 | 41.7 | 44.3 | 45.9 | 43.7 | 43.4 | 42.7 | 42.5 | 41.4 | 40.6 | 40.5 |
| Analândia-SP | 350200 | 10.4 | 10.8 | 11 | 12.6 | 12.5 | 12.2 | 12.7 | 13.2 | 13 | 13.5 | 13.6 |
| Andradina-SP | 350210 | 17.4 | 17.7 | 19.9 | 19.9 | 21.2 | 22.3 | 22.6 | 22.5 | 22.4 | 21.7 | 22.3 |
| Angatuba-SP | 350220 | 12.1 | 12 | 12.8 | 13.4 | 11.1 | 11.9 | 12.3 | 12.3 | 12.1 | 11.8 | 12.4 |
| Anhembi-SP | 350230 | 2.9 | 3.1 | 3.6 | 3.8 | 4.3 | 5.3 | 5.2 | 5.2 | 5.1 | 5 | 4.9 |
| Anhumas-SP | 350240 | 14.5 | 16 | 15.7 | 15.7 | 15.4 | 15 | 14.9 | 15.6 | 15.1 | 16.2 | 16.9 |
| Aparecida-SP | 350250 | 17.3 | 19.7 | 17.7 | 19 | 19.5 | 20.2 | 20.8 | 19.4 | 18 | 16.9 | 16.7 |
| Aparecida d'Oeste-SP | 350260 | 5 | 5.2 | 5.6 | 6 | 8.9 | 7.3 | 7.9 | 8.8 | 9.1 | 11 | 10.7 |
| Apiaí-SP | 350270 | 4.4 | 5.2 | 6 | 5.7 | 6.3 | 7.2 | 8.1 | 6.9 | 6.7 | 6.8 | 6.7 |
| Araçariguama-SP | 350275 | 28.8 | 27.7 | 27.5 | 27.6 | 25.8 | 24.7 | 24.5 | 24.8 | 23.4 | 20.1 | 19.8 |
| Araçatuba-SP | 350280 | 23.6 | 26.2 | 28.3 | 29.3 | 32.2 | 34.2 | 34 | 31.2 | 32.8 | 32.1 | 32.4 |
| Araçoiaba da Serra-SP | 350290 | 17.1 | 17.5 | 19.5 | 22.2 | 23.4 | 25.7 | 26.7 | 26.8 | 25.4 | 24.6 | 23.2 |
| Aramina-SP | 350300 | 11.5 | 17 | 18.5 | 20.3 | 20.7 | 20.3 | 21.9 | 31.9 | 32.1 | 32.6 | 31.8 |
| Arandu-SP | 350310 | 2.9 | 3.7 | 3.6 | 3.6 | 4 | 4.7 | 5.9 | 5.5 | 6.4 | 7.2 | 7.3 |
| Arapeí-SP | 350315 | 2.6 | 4.6 | 3.3 | 4 | 3.4 | 4.1 | 4.1 | 4.4 | 4 | 3.3 | 3.2 |
| Araraquara-SP | 350320 | 51.9 | 49.5 | 51.6 | 50.8 | 51.6 | 50.5 | 51 | 48.6 | 47.8 | 47.3 | 47.3 |
| Araras-SP | 350330 | 43.8 | 45.6 | 51.8 | 56 | 55.9 | 57.6 | 56.9 | 56 | 49.9 | 48.1 | 47.2 |
| Arco-SPÍris-SP | 350335 | 1.3 | 1.6 | 1.8 | 2.3 | 3.1 | 3.6 | 3 | 3.8 | 4.3 | 3.9 | 4.2 |
| Arealva-SP | 350340 | 7.5 | 7.8 | 8.5 | 8.4 | 9.1 | 10.5 | 10.8 | 11.1 | 11.8 | 12.9 | 11.9 |
| Areias-SP | 350350 | 6.4 | 7 | 6.3 | 7 | 7.5 | 7.8 | 7.9 | 8.1 | 7.3 | 5.7 | 6.1 |
| Areiópolis-SP | 350360 | 21.4 | 23 | 26.5 | 24.8 | 24.2 | 24.9 | 24.9 | 23.4 | 21.5 | 21.6 | 20.3 |
| Ariranha-SP | 350370 | 23.1 | 25.6 | 25.1 | 28.3 | 33.2 | 35.2 | 34 | 34.7 | 33.2 | 34.4 | 33.3 |
| Artur Nogueira-SP | 350380 | 31.3 | 33.7 | 38.8 | 31.2 | 31 | 35.3 | 37.3 | 27.1 | 25.3 | 24.3 | 24.3 |
| Arujá-SP | 350390 | 41 | 33.4 | 32.6 | 34.5 | 35.4 | 37.3 | 35.7 | 34.6 | 32.7 | 32.6 | 33.7 |
| Aspásia-SP | 350395 | 3.2 | 15.9 | 14.6 | 4.5 | 6.8 | 6.1 | 7.1 | 7.2 | 6.3 | 6.5 | 6.3 |
| Assis-SP | 350400 | 18 | 18.3 | 17.8 | 18.1 | 19.3 | 19.7 | 19.9 | 20.2 | 20.7 | 20.9 | 21.2 |
| Atibaia-SP | 350410 | 44.9 | 45.5 | 48.1 | 49 | 48.9 | 48.7 | 46.8 | 46.9 | 46 | 46.1 | 45.5 |
| Auriflama-SP | 350420 | 17 | 18.3 | 19.8 | 20.3 | 21.1 | 21.4 | 21.1 | 20.9 | 21.1 | 21.4 | 20.7 |
| Avaí-SP | 350430 | 8.3 | 8.6 | 9.9 | 8.7 | 9.3 | 10.1 | 10.6 | 10.2 | 9.7 | 9.5 | 9.3 |
| Avanhandava-SP | 350440 | 7 | 7.1 | 8 | 13.2 | 14.2 | 15.4 | 15.9 | 19.5 | 14.7 | 13.6 | 11.8 |
| Avaré-SP | 350450 | 14.8 | 16 | 16.4 | 16.2 | 17.4 | 17.5 | 18.2 | 18.2 | 18 | 17.7 | 17.6 |
| Bady Bassitt-SP | 350460 | 26 | 26.5 | 26.9 | 28.4 | 29.8 | 31.2 | 32.9 | 32.5 | 31.7 | 31.8 | 32.3 |
| Balbinos-SP | 350470 | 5.8 | 5.6 | 5.5 | 4.4 | 4.2 | 4.2 | 4.2 | 3.8 | 3.6 | 3.4 | 3.1 |
| Bálsamo-SP | 350480 | 24.3 | 25.1 | 26.4 | 26.3 | 29.5 | 29 | 29.5 | 28.6 | 28.5 | 27.2 | 26.8 |
| Bananal-SP | 350490 | 10.4 | 10.5 | 10.7 | 10.1 | 10.3 | 10.6 | 10.9 | 10.8 | 11 | 10.2 | 9.8 |
| Barão de Antonina-SP | 350500 | 1.3 | 1.4 | 1.3 | 1.3 | 1.4 | 1.3 | 1.7 | 1.5 | 1.3 | 1.6 | 1 |
| Barbosa-SP | 350510 | 4.7 | 5.9 | 5.1 | 6.7 | 8.8 | 10.5 | 10.1 | 7.7 | 7.8 | 8.8 | 4.8 |
| Bariri-SP | 350520 | 16.9 | 16.6 | 11.6 | 11.3 | 11.4 | 11.6 | 11.3 | 11.4 | 11 | 10.7 | 10.6 |
| Barra Bonita-SP | 350530 | 14.3 | 14.8 | 15.4 | 14.8 | 14.8 | 15.8 | 16.1 | 15.5 | 15.2 | 14.9 | 25.6 |
| Barra do Chapéu-SP | 350535 | 0.8 | 0.9 | 1 | 0.7 | 0.4 | 0.5 | 0.4 | 0.3 | 1 | 1 | 0.9 |
| Barra do Turvo-SP | 350540 | 1.2 | 3.1 | 3.8 | 4 | 4.3 | 2.9 | 2.4 | 2.5 | 2.4 | 2.5 | 2.4 |
| Barretos-SP | 350550 | 26.2 | 26.3 | 27.2 | 25.4 | 27 | 28.7 | 29.2 | 30.6 | 28.1 | 24.9 | 24.1 |
| Barrinha-SP | 350560 | 38.4 | 36.4 | 36.1 | 36 | 35.7 | 38 | 38.3 | 37 | 35.7 | 34.7 | 34.9 |
| Barueri-SP | 350570 | 58.5 | 57.2 | 57.6 | 58.9 | 59.7 | 62.3 | 67.1 | 64.2 | 58.8 | 72.9 | 89.5 |
| Bastos-SP | 350580 | 9.9 | 11.5 | 12.5 | 13.3 | 14.3 | 13.6 | 14.5 | 14.9 | 15 | 15.1 | 15 |
| Batatais-SP | 350590 | 23 | 23.6 | 24.3 | 23.6 | 24 | 24.3 | 24.1 | 23.7 | 22.7 | 22.2 | 21.2 |
| Bauru-SP | 350600 | 37.8 | 39.1 | 41.8 | 41.3 | 42.3 | 42 | 43.3 | 43.3 | 42.2 | 41.2 | 40.4 |
| Bebedouro-SP | 350610 | 39.8 | 40.1 | 41.9 | 42.8 | 38.4 | 39 | 38.3 | 38.1 | 37.6 | 36.9 | 36.3 |
| Bento de Abreu-SP | 350620 | 2.8 | 3.9 | 4.6 | 7.6 | 29.7 | 32.2 | 33.5 | 30.2 | 28.8 | 25.3 | 36.7 |
| Bernardino de Campos-SP | 350630 | 6.5 | 6.4 | 6.5 | 6.8 | 7.1 | 7.7 | 7.9 | 8 | 7.9 | 8 | 8 |
| Bertioga-SP | 350635 | 15.5 | 15.2 | 15.3 | 15 | 17.7 | 18.4 | 18.1 | 19 | 19.5 | 20.4 | 18.3 |
| Bilac-SP | 350640 | 9.3 | 10.5 | 10.8 | 10.9 | 12.3 | 12.6 | 12.8 | 13.1 | 12.9 | 12.9 | 12.9 |
| Birigui-SP | 350650 | 17.8 | 23.3 | 24.3 | 25 | 26.8 | 29.2 | 31.3 | 32.5 | 32 | 30.3 | 28.9 |
| Biritiba-SPMirim-SP | 350660 | 11.7 | 13 | 14.5 | 11 | 10.8 | 11.9 | 11.9 | 11.1 | 10.1 | 10.3 | 10.3 |
| Boa Esperança do Sul-SP | 350670 | 12.6 | 13.5 | 13.8 | 14 | 15.5 | 17.9 | 16.2 | 15.7 | 17.3 | 18.7 | 21.4 |
| Bocaina-SP | 350680 | 6.9 | 21.9 | 27.4 | 14.4 | 16.8 | 17.9 | 14.5 | 14.7 | 16.8 | 15.9 | 17.5 |
| Bofete-SP | 350690 | 8.5 | 7.7 | 8.3 | 8.3 | 9.1 | 9.4 | 9 | 9.1 | 8.4 | 8.3 | 8.4 |
| Boituva-SP | 350700 | 34 | 32.4 | 34.3 | 35 | 35.8 | 35.8 | 35.2 | 34.8 | 32.2 | 31.7 | 31.3 |
| Bom Jesus dos Perdões-SP | 350710 | 19 | 19.7 | 22.3 | 23.4 | 24.7 | 23.8 | 24 | 23.2 | 22.7 | 22.8 | 21.3 |
| Bom Sucesso de Itararé-SP | 350715 | 0.9 | 3.4 | 4 | 2.4 | 3.6 | 4.2 | 4.8 | 5.7 | 4.5 | 5.4 | 5.7 |
| Borá-SP | 350720 | 11.8 | 15.4 | 14.9 | 19.5 | 25.8 | 23.9 | 24 | 20.2 | 22.3 | 24.2 | 24.5 |
| Boracéia-SP | 350730 | 6.9 | 13.3 | 9.2 | 8.4 | 8.8 | 8.9 | 8.7 | 7.2 | 7.2 | 7.2 | 7.2 |
| Borborema-SP | 350740 | 12 | 12.1 | 11.7 | 11.6 | 11.1 | 11.6 | 12.2 | 11.8 | 12 | 11.6 | 11.4 |
| Borebi-SP | 350745 | 8.8 | 8.7 | 9.6 | 9.3 | 8.5 | 8.2 | 8.2 | 8.5 | 8.2 | 8.3 | 8.7 |
| Botucatu-SP | 350750 | 28 | 30.8 | 35.4 | 31.4 | 36.1 | 31.9 | 31 | 29.4 | 27.7 | 27.1 | 27.5 |
| Bragança Paulista-SP | 350760 | 35.7 | 37.3 | 38.3 | 39.4 | 41.3 | 41.9 | 40.8 | 40 | 38.2 | 38.8 | 37.9 |
| Braúna-SP | 350770 | 5.4 | 6.2 | 6.8 | 10.2 | 10.3 | 11.3 | 11.6 | 12 | 10 | 8.6 | 8.7 |
| Brejo Alegre-SP | 350775 | 6.2 | 6.3 | 9.4 | 17.9 | 20.3 | 21.1 | 22 | 37.5 | 21.8 | 12.4 | 9.2 |
| Brodowski-SP | 350780 | 22.8 | 23.4 | 23.3 | 22.6 | 24.2 | 25.5 | 25 | 24.2 | 24.1 | 24 | 23.7 |
| Brotas-SP | 350790 | 16.4 | 16.6 | 15.6 | 15.5 | 15.5 | 15.2 | 14.9 | 15.1 | 14.5 | 14.4 | 14.5 |
| Buri-SP | 350800 | 3.3 | 3.2 | 3.4 | 3.6 | 5 | 6 | 6.6 | 3.5 | 3 | 3 | 3.1 |
| Buritama-SP | 350810 | 11.8 | 12.3 | 13 | 13.8 | 14.5 | 16.1 | 17 | 19.3 | 17.9 | 17.2 | 15.4 |
| Buritizal-SP | 350820 | 37.9 | 37.4 | 36.4 | 36.2 | 37.4 | 37.7 | 38.6 | 39.1 | 37.9 | 37 | 36.3 |
| Cabrália Paulista-SP | 350830 | 6.8 | 6.8 | 7.1 | 6.1 | 7.1 | 7.8 | 8 | 8.6 | 8.7 | 8.5 | 8.9 |
| Cabreúva-SP | 350840 | 32.5 | 32.9 | 37.4 | 35.8 | 38.8 | 40.8 | 41.9 | 39.6 | 37.8 | 35.9 | 35.8 |
| Caçapava-SP | 350850 | 38.7 | 40.6 | 40.4 | 41.9 | 42.4 | 42.3 | 41.8 | 39.9 | 37.8 | 37.1 | 36.7 |
| Cachoeira Paulista-SP | 350860 | 22.6 | 25.5 | 26.3 | 26.8 | 27.2 | 28.2 | 28.4 | 27.3 | 26.1 | 21.5 | 20.9 |
| Caconde-SP | 350870 | 16.1 | 17.1 | 13 | 13.4 | 13.4 | 13.3 | 13.4 | 13.3 | 13.4 | 13.1 | 12.7 |
| Cafelândia-SP | 350880 | 20.2 | 20.3 | 20.9 | 20.7 | 21.8 | 21.4 | 21.8 | 21.2 | 20.7 | 20.8 | 20.4 |
| Caiabu-SP | 350890 | 3.5 | 3.7 | 4 | 4.1 | 4.4 | 4.9 | 5.2 | 5.2 | 5.7 | 5.9 | 6.3 |
| Caieiras-SP | 350900 | 41.7 | 41.4 | 37.3 | 36.4 | 38.9 | 38.5 | 39.3 | 37.5 | 33.9 | 33.7 | 31.9 |
| Caiuá-SP | 350910 | 3.6 | 4 | 4.6 | 4.7 | 5.5 | 6.2 | 6.8 | 6.6 | 6.6 | 7 | 7.3 |
| Cajamar-SP | 350920 | 45.5 | 47 | 51.3 | 36.8 | 39.8 | 40.4 | 42.8 | 41.2 | 41.2 | 44.6 | 43 |
| Cajati-SP | 350925 | 14.8 | 10.7 | 14.4 | 16.2 | 16.9 | 15.9 | 15.1 | 16 | 15.8 | 16.2 | 15 |
| Cajobi-SP | 350930 | 14.7 | 16 | 16.6 | 17.7 | 20 | 19.9 | 19.1 | 17.5 | 17.2 | 16.9 | 16.5 |
| Cajuru-SP | 350940 | 18.9 | 19.7 | 20 | 19.9 | 21.8 | 22.7 | 22.5 | 21.5 | 21.2 | 22.1 | 21.6 |
| Campina do Monte Alegre-SP | 350945 | 26.1 | 36.3 | 61.5 | 53.5 | 31.7 | 8.6 | 7.6 | 5.8 | 4.6 | 4.5 | 4.8 |
| Campinas-SP | 350950 | 49.9 | 48.8 | 51.9 | 51.8 | 53.6 | 54.6 | 57.4 | 51.9 | 49.8 | 48.7 | 48.2 |
| Campo Limpo Paulista-SP | 350960 | 36.6 | 38.9 | 43.2 | 41.2 | 42.3 | 43.5 | 44 | 41.6 | 39.2 | 39.2 | 38.3 |
| Campos do Jordão-SP | 350970 | 15.8 | 17.5 | 17.9 | 17.7 | 18.2 | 13.2 | 13.3 | 13.1 | 12.4 | 11.8 | 11.2 |
| Campos Novos Paulista-SP | 350980 | 2.4 | 2.4 | 2.4 | 2.8 | 2.8 | 3.2 | 3.4 | 3.7 | 3.5 | 3.6 | 3.7 |
| Cananéia-SP | 350990 | 4.8 | 4.9 | 4.7 | 5 | 5.2 | 5.5 | 5.7 | 5.6 | 5.8 | 5.7 | 5.5 |
| Canas-SP | 350995 | 7.4 | 7.5 | 7.7 | 7.7 | 7.8 | 7.9 | 10 | 9.8 | 9.6 | 9.6 | 8 |
| Cândido Mota-SP | 351000 | 6.9 | 7.4 | 8.6 | 9.4 | 10.5 | 11.5 | 12.4 | 13.1 | 14.9 | 15.4 | 15.9 |
| Cândido Rodrigues-SP | 351010 | 24.2 | 24.6 | 24.9 | 25.5 | 26.3 | 26.4 | 27 | 26.9 | 25.6 | 24.9 | 24.9 |
| Canitar-SP | 351015 | 9.8 | 10.7 | 12.7 | 16.5 | 17.6 | 17.4 | 16.4 | 15.7 | 15.8 | 15.2 | 14.9 |
| Capão Bonito-SP | 351020 | 6.4 | 6.3 | 7.1 | 7.6 | 8 | 8.7 | 8.8 | 8.8 | 8.2 | 8.1 | 8 |
| Capela do Alto-SP | 351030 | 7.7 | 7.7 | 8.3 | 10.5 | 11.3 | 11.8 | 12.3 | 12.6 | 12.2 | 11.7 | 11.2 |
| Capivari-SP | 351040 | 37.9 | 37.7 | 39 | 38.1 | 39.2 | 41.4 | 40.2 | 37.6 | 36.7 | 38 | 38.1 |
| Caraguatatuba-SP | 351050 | 21.5 | 27.3 | 28 | 21.7 | 22.8 | 23.8 | 24.5 | 23.8 | 22.5 | 21.7 | 20.6 |
| Carapicuíba-SP | 351060 | 34.8 | 35.6 | 36.8 | 37.7 | 38.2 | 39.9 | 40.1 | 37.6 | 35 | 34.4 | 33.5 |
| Cardoso-SP | 351070 | 7 | 10 | 13.8 | 17.7 | 15.7 | 16.1 | 16.6 | 16.2 | 15.6 | 16.4 | 14 |
| Casa Branca-SP | 351080 | 19.4 | 22.6 | 23.6 | 22.8 | 22.9 | 22.2 | 24 | 23.1 | 22.3 | 19.6 | 19.5 |
| Cássia dos Coqueiros-SP | 351090 | 5.7 | 6 | 6.7 | 7.1 | 7.7 | 8.1 | 8.6 | 8.5 | 7.9 | 8.2 | 8.4 |
| Castilho-SP | 351100 | 15.4 | 16.5 | 16.9 | 14.2 | 17.5 | 18.2 | 17.4 | 16.4 | 16.7 | 15.8 | 15.9 |
| Catanduva-SP | 351110 | 33.9 | 35 | 37.3 | 36.2 | 37.8 | 38.9 | 39 | 38.3 | 36.7 | 37.3 | 37.2 |
| Catiguá-SP | 351120 | 42.9 | 46.3 | 50.4 | 57.6 | 55.5 | 53.9 | 52.8 | 46.3 | 44.6 | 44.5 | 43.6 |
| Cedral-SP | 351130 | 23.9 | 24 | 25.2 | 25.6 | 26.4 | 27.2 | 28.7 | 28.9 | 28.3 | 28.6 | 28.5 |
| Cerqueira César-SP | 351140 | 8.3 | 10.2 | 9.6 | 9.3 | 9.5 | 10.5 | 11.2 | 9.1 | 7.8 | 7.8 | 7.2 |
| Cerquilho-SP | 351150 | 29.8 | 29.6 | 29.6 | 29.6 | 30.5 | 31.9 | 34.2 | 33.6 | 31.5 | 33.9 | 34.1 |
| Cesário Lange-SP | 351160 | 9.5 | 10.1 | 13.7 | 13.9 | 14.3 | 15.7 | 15.8 | 15.6 | 15 | 14.8 | 14.1 |
| Charqueada-SP | 351170 | 31.6 | 31.6 | 31.3 | 29.9 | 32.2 | 39.8 | 38.7 | 35.4 | 35.9 | 35.8 | 35.7 |
| Clementina-SP | 351190 | 4.4 | 5.1 | 5.3 | 5.7 | 6.3 | 7 | 7 | 7.3 | 6.6 | 6.6 | 7 |
| Colina-SP | 351200 | 23.5 | 23.4 | 25.9 | 32.5 | 36.8 | 36.6 | 38.9 | 39.4 | 35.3 | 37.7 | 35.8 |
| Colômbia-SP | 351210 | 14.1 | 11.4 | 18.3 | 8.7 | 11.4 | 14.1 | 21 | 24.3 | 21.7 | 20.4 | 22.9 |
| Conchal-SP | 351220 | 20.9 | 21.7 | 22.5 | 26.4 | 28.1 | 28.3 | 28.3 | 26.1 | 22.1 | 22.8 | 22.2 |
| Conchas-SP | 351230 | 5 | 5.1 | 5.4 | 5 | 5 | 5.4 | 5.5 | 5.7 | 5.8 | 5.7 | 5.9 |
| Cordeirópolis-SP | 351240 | 38.2 | 38.1 | 42.8 | 42.4 | 43.7 | 44.3 | 45.4 | 40.4 | 42.3 | 41.2 | 40.8 |
| Coroados-SP | 351250 | 9.1 | 9.8 | 11.9 | 14 | 15.7 | 17.7 | 18.8 | 24.9 | 19.3 | 16.4 | 16 |
| Coronel Macedo-SP | 351260 | 1.9 | 2.3 | 2.1 | 1.8 | 1.8 | 2.1 | 1.9 | 2.9 | 2.5 | 1.4 | 2.2 |
| Corumbataí-SP | 351270 | 20.5 | 21 | 22 | 24.2 | 26.1 | 29.5 | 29.4 | 28.6 | 27.7 | 28 | 28.1 |
| Cosmópolis-SP | 351280 | 32.6 | 35 | 33.8 | 38.7 | 45.3 | 38.3 | 37.1 | 30.2 | 26.7 | 25.6 | 25.9 |
| Cosmorama-SP | 351290 | 11.1 | 12.9 | 14.4 | 13.9 | 14 | 15.9 | 17.2 | 15.8 | 15.8 | 16.1 | 16.6 |
| Cotia-SP | 351300 | 37.4 | 37.7 | 38 | 39.4 | 40.8 | 41.1 | 40.9 | 39.3 | 34.6 | 33 | 32.5 |
| Cravinhos-SP | 351310 | 36.4 | 37.5 | 40.7 | 38.7 | 39.5 | 40.6 | 41.5 | 38.5 | 32.2 | 36.3 | 35.6 |
| Cristais Paulista-SP | 351320 | 17.4 | 17.8 | 18.4 | 19 | 19.1 | 19.6 | 19.6 | 20 | 20 | 20.1 | 19.8 |
| Cruzália-SP | 351330 | 3.5 | 4.3 | 4.6 | 5.9 | 6.4 | 8 | 7.3 | 5.7 | 6.5 | 6.6 | 6.9 |
| Cruzeiro-SP | 351340 | 49.6 | 46.5 | 51.8 | 52.2 | 49.8 | 51.9 | 49.3 | 43.2 | 40.6 | 36.1 | 39.4 |
| Cubatão-SP | 351350 | 54.8 | 51.9 | 44.4 | 43.1 | 39.4 | 44.2 | 43.8 | 39.2 | 33.1 | 31.3 | 32.3 |
| Cunha-SP | 351360 | 2.9 | 3.1 | 3.5 | 3.3 | 4.7 | 4.2 | 4 | 4.1 | 4 | 3.9 | 3.8 |
| Descalvado-SP | 351370 | 26 | 23.2 | 31.8 | 31.7 | 31.6 | 31.6 | 32.5 | 33 | 33 | 34.7 | 34.4 |
| Diadema-SP | 351380 | 48.8 | 47.8 | 48.5 | 48.1 | 46.9 | 47.6 | 46.9 | 44.3 | 41.5 | 40.9 | 41.4 |
| Dirce Reis-SP | 351385 | 5.9 | 5.5 | 6.6 | 8.9 | 10.3 | 9.1 | 7 | 6.7 | 6.9 | 6.6 | 6.2 |
| Divinolândia-SP | 351390 | 8.9 | 9.7 | 10.2 | 11 | 11.7 | 11.3 | 11 | 11 | 10.9 | 9.9 | 10 |
| Dobrada-SP | 351400 | 28.3 | 28.7 | 27.8 | 26 | 25.3 | 28.3 | 29 | 26.3 | 25.9 | 27.9 | 27.9 |
| Dois Córregos-SP | 351410 | 13.1 | 12.6 | 12.7 | 13.1 | 13.5 | 14 | 13.4 | 13 | 12.4 | 11.9 | 15.2 |
| Dolcinópolis-SP | 351420 | 4 | 5.1 | 8.8 | 10.7 | 14.1 | 13.1 | 13.4 | 15.2 | 14.5 | 14.8 | 12.7 |
| Dourado-SP | 351430 | 9.7 | 18.4 | 24 | 17.5 | 20.7 | 22.4 | 22.3 | 22.9 | 24.4 | 24.9 | 24.3 |
| Dracena-SP | 351440 | 18.6 | 19.2 | 19.8 | 19.5 | 20.6 | 21.2 | 21.3 | 17.4 | 16.7 | 16 | 15.5 |
| Duartina-SP | 351450 | 14.5 | 14.3 | 15 | 14.3 | 14.7 | 15.3 | 16.1 | 16.2 | 15.7 | 15.6 | 15.5 |
| Dumont-SP | 351460 | 37.7 | 39.6 | 40.2 | 40.8 | 41.8 | 43.5 | 44.4 | 44.2 | 43.5 | 41.9 | 41.3 |
| Echaporã-SP | 351470 | 12.6 | 13 | 13.9 | 9.8 | 9.9 | 10.6 | 11.1 | 10.6 | 10.8 | 10.7 | 11.5 |
| Eldorado-SP | 351480 | 5.5 | 5.6 | 5.5 | 5.4 | 5.4 | 5.2 | 5.1 | 4.9 | 4.7 | 4.6 | 4.5 |
| Elias Fausto-SP | 351490 | 24.2 | 24.5 | 26.8 | 27.7 | 26.9 | 23.8 | 17.6 | 17.2 | 21.3 | 20.3 | 21.1 |
| Elisiário-SP | 351492 | 20.7 | 20.9 | 27.3 | 33.9 | 33.6 | 32.1 | 33.9 | 31.8 | 30.3 | 30.4 | 29.9 |
| Embaúba-SP | 351495 | 16.2 | 18.3 | 22.2 | 27.5 | 31.4 | 29.9 | 28.4 | 24.4 | 24.1 | 24 | 25.6 |
| Embu das Artes-SP | 351500 | 26.6 | 27.4 | 27.4 | 28.7 | 28.8 | 29.4 | 29.1 | 27.1 | 24.6 | 23.7 | 22.7 |
| Embu-SPGuaçu-SP | 351510 | 15.2 | 15.3 | 13.5 | 14.7 | 15.1 | 16.3 | 15.8 | 14.5 | 13.3 | 13 | 12.9 |
| Emilianópolis-SP | 351512 | 9 | 10.1 | 10 | 10.6 | 11.3 | 11.8 | 11.5 | 11.8 | 12.1 | 12.3 | 12.3 |
| Engenheiro Coelho-SP | 351515 | 15 | 17.7 | 19.5 | 20 | 20.3 | 20.3 | 20.5 | 18.3 | 18.6 | 19.1 | 19.1 |
| Espírito Santo do Pinhal-SP | 351518 | 43.2 | 39.9 | 40.5 | 41.5 | 42.2 | 42.5 | 42 | 40.9 | 40.8 | 39.9 | 38.6 |
| Espírito Santo do Turvo-SP | 351519 | 1.2 | 2.4 | 4.3 | 4.4 | 4.1 | 5.9 | 6 | 6 | 6.5 | 5.7 | 5.5 |
| Estrela d'Oeste-SP | 351520 | 6.1 | 8.7 | 11.3 | 11.6 | 11.8 | 12.5 | 13.1 | 13 | 12.6 | 12.5 | 13.5 |
| Estrela do Norte-SP | 351530 | 29.5 | 34.1 | 33.4 | 36.1 | 34.7 | 26.1 | 25.5 | 24.3 | 23.6 | 24 | 24.7 |
| Euclides da Cunha Paulist-SP | 351535 | 2.2 | 2.5 | 2.4 | 6.1 | 8.9 | 11.2 | 11 | 9.1 | 7.2 | 7.1 | 6 |
| Fartura-SP | 351540 | 3.9 | 3.7 | 3.7 | 3.8 | 3.9 | 3.9 | 3.9 | 3.9 | 3.6 | 3.4 | 3.5 |
| Fernandópolis-SP | 351550 | 21.8 | 22.5 | 22.6 | 19.8 | 20 | 20.6 | 21.5 | 21.2 | 20.9 | 21.3 | 21.6 |
| Fernando Prestes-SP | 351560 | 27.7 | 28.3 | 30.1 | 32.6 | 34.8 | 33.7 | 32.4 | 32.3 | 31.1 | 30.9 | 30.9 |
| Fernão-SP | 351565 | 1.1 | 2.9 | 3.8 | 3.5 | 2.5 | 2.6 | 2.8 | 3.2 | 2.8 | 3.3 | 3.5 |
| Ferraz de Vasconcelos-SP | 351570 | 18.5 | 17.3 | 17.7 | 18.7 | 19.6 | 21.1 | 21.4 | 20.2 | 18.6 | 18.2 | 18 |
| Flora Rica-SP | 351580 | 4 | 4 | 3.5 | 3.3 | 3.6 | 4.6 | 4.9 | 5.1 | 4.9 | 7.1 | 7.3 |
| Floreal-SP | 351590 | 21 | 25.9 | 25.6 | 23.5 | 22.8 | 23.4 | 22.8 | 27.1 | 23 | 22.4 | 20.7 |
| Flórida Paulista-SP | 351600 | 2.9 | 2.9 | 3.2 | 3.2 | 3.7 | 4 | 4.7 | 5.4 | 5.4 | 7.7 | 8.2 |
| Florínia-SP | 351610 | 2 | 2.4 | 2.4 | 3.7 | 4.2 | 4.6 | 5.4 | 5.6 | 8.1 | 8.7 | 10.5 |
| Franca-SP | 351620 | 32.8 | 32.7 | 33.6 | 34 | 33.8 | 34.3 | 35.3 | 35 | 34.9 | 34.1 | 33.1 |
| Francisco Morato-SP | 351630 | 17.4 | 19.3 | 20.6 | 22.1 | 24.1 | 25.4 | 26.5 | 24.9 | 23.2 | 21.9 | 20.5 |
| Franco da Rocha-SP | 351640 | 24.5 | 26 | 26.2 | 26.9 | 27.6 | 28.3 | 28.1 | 26.4 | 24.1 | 23 | 21.8 |
| Gabriel Monteiro-SP | 351650 | 5.2 | 8.9 | 10 | 12.2 | 17.6 | 18.4 | 19.7 | 20.6 | 20.4 | 21.5 | 21.3 |
| Gália-SP | 351660 | 7.8 | 8.1 | 8.7 | 9.3 | 9.8 | 10.8 | 11.3 | 11.8 | 11.9 | 12 | 12.4 |
| Garça-SP | 351670 | 11.4 | 11.6 | 12 | 12.1 | 12.5 | 14.4 | 14.7 | 13.5 | 13.2 | 13.2 | 12.9 |
| Gastão Vidigal-SP | 351680 | 13.7 | 22.6 | 20.1 | 19.1 | 21.1 | 22.7 | 20 | 20.4 | 22.9 | 22.5 | 12.4 |
| Gavião Peixoto-SP | 351685 | 27.4 | 28.4 | 28.3 | 30.9 | 38.9 | 40.9 | 40.5 | 36.2 | 35 | 35.7 | 35.6 |
| General Salgado-SP | 351690 | 17.8 | 19 | 19.5 | 20.6 | 23.5 | 25.5 | 24.8 | 24.5 | 25.8 | 25.6 | 25.3 |
| Getulina-SP | 351700 | 19.4 | 19.6 | 20.1 | 22.1 | 23.1 | 23.7 | 23.3 | 24.5 | 20.3 | 19.3 | 18.1 |
| Glicério-SP | 351710 | 9.6 | 10.3 | 12.3 | 18.2 | 19 | 19.3 | 18.7 | 27 | 15.5 | 10.3 | 8.6 |
| Guaiçara-SP | 351720 | 31.7 | 31.7 | 31.3 | 33.4 | 35.6 | 34.1 | 32.9 | 36.6 | 29.6 | 27.7 | 26.8 |
| Guaimbê-SP | 351730 | 14.9 | 15.2 | 16.1 | 16.7 | 18.1 | 20.5 | 18.4 | 19.1 | 16 | 14.5 | 14.2 |
| Guaíra-SP | 351740 | 25 | 26.3 | 27.1 | 29.9 | 34.7 | 37.2 | 38.4 | 41.1 | 41.4 | 34.7 | 39.8 |
| Guapiaçu-SP | 351750 | 25.8 | 27.1 | 24.4 | 24.8 | 27.2 | 27.9 | 28.8 | 29.9 | 29.4 | 29 | 28.9 |
| Guapiara-SP | 351760 | 1.3 | 1.3 | 1.6 | 1.6 | 1.8 | 2 | 2.1 | 1.5 | 1.7 | 1.8 | 1.8 |
| Guará-SP | 351770 | 35.5 | 33.7 | 35.6 | 31.6 | 25.6 | 34.9 | 34.6 | 30.5 | 28.8 | 27.4 | 25.9 |
| Guaraçaí-SP | 351780 | 7.4 | 7.7 | 8 | 8 | 9.3 | 11.5 | 11.2 | 10.8 | 11.3 | 11.3 | 12.8 |
| Guaraci-SP | 351790 | 14.3 | 13.6 | 12.9 | 6 | 9.2 | 11.1 | 12.5 | 19.7 | 20.4 | 20.8 | 20.2 |
| Guarani d'Oeste-SP | 351800 | 3.7 | 5 | 6.3 | 7.1 | 7.5 | 7.3 | 7.9 | 7.6 | 7.4 | 8.2 | 8.3 |
| Guarantã-SP | 351810 | 9.6 | 10.1 | 10.3 | 11.3 | 13.3 | 13.4 | 13.1 | 13.6 | 12.8 | 12.7 | 12.4 |
| Guararapes-SP | 351820 | 9.3 | 9.7 | 12.1 | 13.1 | 22.5 | 24.8 | 23.5 | 25.2 | 24.9 | 26.6 | 28.1 |
| Guararema-SP | 351830 | 19.4 | 21.9 | 23 | 22.4 | 21.2 | 20.8 | 21 | 20 | 17.8 | 17.1 | 18.1 |
| Guaratinguetá-SP | 351840 | 23.6 | 26.3 | 27.5 | 27.4 | 26.9 | 27.3 | 27.9 | 24.2 | 22.3 | 21.8 | 21.6 |
| Guareí-SP | 351850 | 2.4 | 2.4 | 3.1 | 3.3 | 3.8 | 3.9 | 2.8 | 2.8 | 2.7 | 2.7 | 2.6 |
| Guariba-SP | 351860 | 44.5 | 44.2 | 41.3 | 37.9 | 39.1 | 38.7 | 41.2 | 38.3 | 38.1 | 36.9 | 35.6 |
| Guarujá-SP | 351870 | 34 | 34.2 | 36.7 | 37.1 | 36.8 | 38.3 | 38.6 | 37.8 | 36.8 | 36.1 | 34.3 |
| Guarulhos-SP | 351880 | 42.3 | 43.1 | 46.1 | 45.8 | 44.3 | 44.5 | 43.1 | 41.4 | 39.1 | 38.7 | 38.8 |
| Guatapará-SP | 351885 | 19.6 | 23.2 | 22.4 | 26 | 27.5 | 29 | 30.3 | 28.7 | 28.7 | 27.8 | 28.6 |
| Guzolândia-SP | 351890 | 16 | 20.8 | 35.3 | 39.8 | 54.7 | 34.6 | 29.4 | 33.6 | 25.7 | 19.1 | 15.3 |
| Herculândia-SP | 351900 | 6.8 | 7.5 | 8.5 | 8.9 | 9.1 | 9.2 | 9.1 | 8.9 | 8.6 | 8.5 | 7.9 |
| Holambra-SP | 351905 | 31.7 | 32.2 | 35 | 33.5 | 33.3 | 33.5 | 36 | 30.8 | 29.9 | 29.2 | 29.5 |
| Hortolândia-SP | 351907 | 25.7 | 26.5 | 30.7 | 32.2 | 35 | 36.3 | 38.2 | 33.6 | 31.8 | 31.3 | 31.2 |
| Iacanga-SP | 351910 | 8.3 | 8.5 | 9.1 | 8.5 | 8.5 | 9 | 9.1 | 8.7 | 21.6 | 39.3 | 22.4 |
| Iacri-SP | 351920 | 5.5 | 6.2 | 9.1 | 10.2 | 10.7 | 10.1 | 11.3 | 11.1 | 11.2 | 11.2 | 10.8 |
| Iaras-SP | 351925 | 5.6 | 6.2 | 5.9 | 4.6 | 4.2 | 4.2 | 7.8 | 6.9 | 6.8 | 6.4 | 6.3 |
| Ibaté-SP | 351930 | 18.8 | 19 | 19.5 | 19.1 | 21.1 | 23.3 | 22.8 | 21.8 | 21.8 | 20.6 | 20.8 |
| Ibirá-SP | 351940 | 14.8 | 15.2 | 18.8 | 21.4 | 23.7 | 23.9 | 25 | 22.5 | 22.3 | 21.8 | 21.5 |
| Ibirarema-SP | 351950 | 6.8 | 7.3 | 8 | 8.4 | 8.6 | 9.6 | 9.8 | 9.2 | 9.5 | 9.2 | 9.2 |
| Ibitinga-SP | 351960 | 23.6 | 21.9 | 20 | 24.3 | 23.7 | 22.7 | 22.3 | 19.1 | 18.8 | 19.1 | 18.7 |
| Ibiúna-SP | 351970 | 10 | 10.3 | 10.7 | 11.3 | 11.9 | 12.7 | 13.6 | 13.9 | 14.5 | 13 | 12.9 |
| Icém-SP | 351980 | 18 | 20.5 | 24.6 | 26.6 | 27.3 | 26.7 | 26.6 | 24.6 | 24.8 | 26.9 | 24.5 |
| Iepê-SP | 351990 | 10.4 | 10.7 | 10.8 | 11.1 | 11.6 | 12.6 | 11.8 | 12.3 | 12.6 | 12.5 | 12 |
| Igaraçu do Tietê-SP | 352000 | 6.3 | 7.3 | 7.7 | 7.2 | 7.5 | 8 | 8.2 | 8 | 7.6 | 7.8 | 21.8 |
| Igarapava-SP | 352010 | 21.5 | 25.2 | 26 | 27.1 | 26.5 | 23.8 | 23.9 | 30.4 | 29.9 | 30.7 | 30.8 |
| Igaratá-SP | 352020 | 9.2 | 9.3 | 9.9 | 9.7 | 9.8 | 10.6 | 10.9 | 12.5 | 12.4 | 12.1 | 11 |
| Iguape-SP | 352030 | 8.1 | 8.1 | 8 | 7.6 | 7.7 | 7.8 | 7.9 | 5.5 | 5.4 | 5.1 | 5 |
| Ilhabela-SP | 352040 | 8.5 | 8.2 | 7.9 | 8.1 | 8.5 | 9.1 | 9.7 | 9.8 | 10.3 | 10.3 | 9.8 |
| Ilha Comprida-SP | 352042 | 4.9 | 4.9 | 4.9 | 4.6 | 4.7 | 4.9 | 5.1 | 5.3 | 5.1 | 4.9 | 4.3 |
| Ilha Solteira-SP | 352044 | 27.2 | 28.1 | 28.9 | 28.9 | 34.3 | 30 | 30.7 | 29.8 | 29.2 | 29.2 | 28.7 |
| Indaiatuba-SP | 352050 | 39.6 | 39.6 | 43.2 | 43.6 | 45.2 | 46.3 | 45.2 | 43 | 41 | 38.6 | 38.8 |
| Indiana-SP | 352060 | 11.8 | 12.5 | 12.9 | 13.7 | 14.4 | 14.5 | 15.6 | 16.1 | 15.7 | 15.3 | 15.8 |
| Indiaporã-SP | 352070 | 8.7 | 9.9 | 12.2 | 12.4 | 14.8 | 14.2 | 12.8 | 12.8 | 12.2 | 14.3 | 13.3 |
| Inúbia Paulista-SP | 352080 | 5.2 | 5.5 | 6.3 | 5.8 | 5.8 | 5.4 | 5.8 | 5.6 | 5.3 | 5.7 | 5.7 |
| Ipaussu-SP | 352090 | 20.3 | 19.6 | 20.6 | 20.7 | 21.5 | 21.2 | 19.9 | 18.2 | 18.1 | 17.8 | 17.9 |
| Iperó-SP | 352100 | 17.9 | 16.7 | 18.9 | 20.6 | 20.6 | 20.8 | 20 | 17.4 | 15.7 | 14.9 | 13.8 |
| Ipeúna-SP | 352110 | 28.1 | 28.1 | 30.4 | 29.4 | 32.1 | 38.1 | 28 | 25.6 | 23.6 | 23.1 | 23.3 |
| Ipiguá-SP | 352115 | 12.4 | 12.9 | 13.9 | 14.4 | 15.8 | 18.6 | 18.9 | 19.1 | 19.2 | 18.7 | 19 |
| Iporanga-SP | 352120 | 3.2 | 3.3 | 3.4 | 2.9 | 2.8 | 3 | 3 | 2.9 | 2.8 | 2.4 | 2.2 |
| Ipuã-SP | 352130 | 32.3 | 27.9 | 27.9 | 26 | 27.9 | 33.7 | 32.3 | 33.3 | 33.1 | 34 | 33.5 |
| Iracemápolis-SP | 352140 | 53.5 | 52.6 | 53.4 | 52.6 | 53.3 | 54.7 | 54.7 | 53 | 51.2 | 50.3 | 49.5 |
| Irapuã-SP | 352150 | 6.1 | 6.7 | 7.5 | 12 | 12.4 | 13.3 | 13.8 | 13.2 | 13.9 | 14.6 | 15.6 |
| Irapuru-SP | 352160 | 8.9 | 8.8 | 8.7 | 8 | 9.2 | 9.4 | 9.8 | 7.9 | 8.1 | 9 | 9.1 |
| Itaberá-SP | 352170 | 1.9 | 2 | 2.1 | 2.8 | 3 | 3.8 | 3.8 | 4.4 | 4.3 | 4.6 | 5 |
| Itaí-SP | 352180 | 6 | 6.5 | 6.4 | 6.2 | 6.2 | 7.3 | 7.8 | 8.1 | 8.1 | 7.8 | 7.9 |
| Itajobi-SP | 352190 | 14.3 | 14.7 | 15.5 | 16.1 | 17.4 | 17.8 | 18.5 | 20 | 19.4 | 19.9 | 20.7 |
| Itaju-SP | 352200 | 5.3 | 5.3 | 4.8 | 4.8 | 4.9 | 4.7 | 4.7 | 4.5 | 4.4 | 4.6 | 4.4 |
| Itanhaém-SP | 352210 | 13.9 | 14.1 | 14.6 | 14.1 | 14.8 | 15.4 | 15.7 | 16.2 | 16.1 | 16.2 | 15.5 |
| Itaóca-SP | 352215 | 1.8 | 2 | 1.7 | 2.5 | 2.2 | 2.1 | 2.3 | 2.1 | 2.3 | 2.3 | 2.2 |
| Itapecerica da Serra-SP | 352220 | 22 | 22.3 | 23 | 24.2 | 25.1 | 26.1 | 26.3 | 24.9 | 23.9 | 22.9 | 21.7 |
| Itapetininga-SP | 352230 | 16.7 | 18 | 18.8 | 18.7 | 20.2 | 20.5 | 20.2 | 19.6 | 19.4 | 18.9 | 19.3 |
| Itapeva-SP | 352240 | 12.2 | 12.1 | 13.1 | 13.9 | 15.7 | 17.2 | 18.1 | 16.2 | 16.9 | 18 | 18.1 |
| Itapevi-SP | 352250 | 24.6 | 24.8 | 27.1 | 28.6 | 28.4 | 28.8 | 29 | 27.4 | 25.4 | 24.8 | 24.2 |
| Itapira-SP | 352260 | 26.5 | 26.9 | 27.5 | 29.8 | 30.7 | 30.3 | 30.2 | 30.2 | 28.5 | 28.6 | 28.5 |
| Itapirapuã Paulista-SP | 352265 | 1.6 | 1.7 | 1.6 | 1.9 | 2.3 | 2.1 | 2.1 | 1.6 | 1.7 | 1.6 | 2 |
| Itápolis-SP | 352270 | 13.5 | 13.9 | 13.8 | 15.6 | 16 | 16 | 17.5 | 18.1 | 17.7 | 17.5 | 17.1 |
| Itaporanga-SP | 352280 | 2.9 | 3.5 | 3.4 | 3.4 | 3.4 | 3.6 | 3.8 | 3.7 | 3.5 | 3 | 3 |
| Itapuí-SP | 352290 | 6.7 | 6.8 | 8 | 7.9 | 7.8 | 8.5 | 8.2 | 7.8 | 6.9 | 6.6 | 6.5 |
| Itapura-SP | 352300 | 6.4 | 7 | 8.8 | 10.2 | 12.6 | 11.7 | 12.1 | 10.8 | 9.2 | 10.1 | 10 |
| Itaquaquecetuba-SP | 352310 | 22 | 18.5 | 19.1 | 20.6 | 21.5 | 22.7 | 23 | 21.7 | 20.6 | 20.2 | 20.9 |
| Itararé-SP | 352320 | 4.8 | 4.6 | 4.7 | 4.9 | 5.6 | 6.7 | 7.1 | 7.1 | 8.9 | 8.7 | 8.8 |
| Itariri-SP | 352330 | 9.2 | 9 | 8.8 | 7.8 | 7.4 | 7 | 6.7 | 7.5 | 6.3 | 6.1 | 5.9 |
| Itatiba-SP | 352340 | 37.4 | 40.7 | 45.6 | 39.3 | 41.4 | 40.8 | 39.5 | 36.8 | 34.1 | 33.9 | 33.4 |
| Itatinga-SP | 352350 | 15.4 | 17.5 | 19.1 | 22.7 | 26.7 | 26.8 | 26.7 | 26.3 | 23.8 | 23.6 | 23.2 |
| Itirapina-SP | 352360 | 11.1 | 12.6 | 14.5 | 14.9 | 16 | 14.8 | 16 | 15.9 | 15.6 | 15 | 14 |
| Itirapuã-SP | 352370 | 13.5 | 13.5 | 13.6 | 14.5 | 15.4 | 15.3 | 14.9 | 19.9 | 15.8 | 15.3 | 15.4 |
| Itobi-SP | 352380 | 17 | 20.4 | 21.5 | 21.9 | 26.3 | 27.1 | 34 | 32.8 | 28.2 | 19.5 | 20.2 |
| Itu-SP | 352390 | 38.9 | 40.3 | 43.6 | 43.8 | 45.1 | 44.9 | 44.4 | 42.4 | 41 | 37.6 | 37.6 |
| Itupeva-SP | 352400 | 41.6 | 41.3 | 45.4 | 43.5 | 44.3 | 45.9 | 46.6 | 44.5 | 44.2 | 44.1 | 43.8 |
| Ituverava-SP | 352410 | 26.2 | 30.6 | 32.3 | 31.8 | 33 | 33.9 | 33.3 | 33 | 33 | 33 | 33.1 |
| Jaborandi-SP | 352420 | 14.7 | 15.3 | 17.8 | 19.7 | 23 | 21.8 | 22.2 | 20.3 | 19 | 19.6 | 18.6 |
| Jaboticabal-SP | 352430 | 48.5 | 47.9 | 48 | 45.4 | 48.5 | 46 | 45.4 | 44.5 | 43.3 | 42.6 | 40.7 |
| Jacareí-SP | 352440 | 41.6 | 43 | 46.7 | 46.9 | 45.9 | 45.9 | 45.8 | 45.5 | 42 | 42.8 | 41 |
| Jaci-SP | 352450 | 34 | 31.8 | 29 | 31.4 | 33.2 | 32 | 32.1 | 30.5 | 28.9 | 28.4 | 28.1 |
| Jacupiranga-SP | 352460 | 7.9 | 8.6 | 9.6 | 9.4 | 9.5 | 9.6 | 9.5 | 9 | 8.9 | 8.7 | 8.5 |
| Jaguariúna-SP | 352470 | 35.8 | 33.3 | 35.3 | 35.4 | 35.9 | 36.7 | 38.1 | 37.7 | 35.3 | 35 | 33.4 |
| Jales-SP | 352480 | 14.5 | 16.5 | 18.5 | 19.7 | 20.4 | 20.7 | 21.3 | 21.7 | 21.9 | 22.2 | 22.3 |
| Jambeiro-SP | 352490 | 42.2 | 40.2 | 46.2 | 31.2 | 27.6 | 45.6 | 31.9 | 25.6 | 20.2 | 23.1 | 21.4 |
| Jandira-SP | 352500 | 30 | 30.8 | 33.3 | 35 | 34.3 | 35.8 | 36.3 | 34 | 31.5 | 29.9 | 29.5 |
| Jardinópolis-SP | 352510 | 29.3 | 27.1 | 29.1 | 28.1 | 29.2 | 29.9 | 29.6 | 29.1 | 28.9 | 28.2 | 27.9 |
| Jarinu-SP | 352520 | 18.3 | 19.2 | 21.3 | 24.2 | 26.7 | 29.5 | 31.3 | 30 | 28.7 | 30.5 | 30 |
| Jaú-SP | 352530 | 16.1 | 16 | 17.8 | 17.1 | 17.6 | 18.2 | 18.2 | 17.7 | 17.4 | 16.9 | 16.6 |
| Jeriquara-SP | 352540 | 13 | 15 | 15.3 | 15.3 | 16 | 16 | 16.8 | 16.6 | 16.8 | 18.5 | 19 |
| Joanópolis-SP | 352550 | 15.6 | 16.2 | 16.9 | 17.6 | 17.6 | 17.8 | 16.5 | 16.8 | 16 | 15.5 | 14.3 |
| João Ramalho-SP | 352560 | 22.9 | 24.5 | 25.6 | 24.6 | 22.7 | 21.7 | 16.8 | 19.7 | 19 | 20 | 24.1 |
| José Bonifácio-SP | 352570 | 20.1 | 20.9 | 21.1 | 22.2 | 24.5 | 26.2 | 27.1 | 25.7 | 25.1 | 26.2 | 24.3 |
| Júlio Mesquita-SP | 352580 | 5.2 | 5.6 | 6.5 | 7.3 | 8.8 | 10.9 | 10.5 | 10.2 | 10.1 | 10.2 | 10.2 |
| Jumirim-SP | 352585 | 13.4 | 12.9 | 15.1 | 15 | 15.1 | 22.2 | 21 | 21.6 | 23.8 | 25.2 | 26 |
| Jundiaí-SP | 352590 | 64.7 | 66.3 | 70.5 | 64.3 | 65.3 | 66.2 | 65 | 61.6 | 60.2 | 60.1 | 59.9 |
| Junqueirópolis-SP | 352600 | 10.9 | 11.4 | 11.5 | 11.7 | 13.6 | 14.5 | 17.2 | 16.1 | 16.4 | 18 | 17.6 |
| Juquiá-SP | 352610 | 7.5 | 7.4 | 7.5 | 6.4 | 7.5 | 7.4 | 7.7 | 7.6 | 7.4 | 7.4 | 7.3 |
| Juquitiba-SP | 352620 | 7.5 | 6.5 | 6.6 | 7.8 | 8.1 | 8 | 7.7 | 7.6 | 7.5 | 7.1 | 6.9 |
| Lagoinha-SP | 352630 | 3.8 | 4.2 | 4.9 | 4.5 | 5 | 4.9 | 4.7 | 4.8 | 4.9 | 4.9 | 4.9 |
| Laranjal Paulista-SP | 352640 | 20.9 | 22.1 | 21.1 | 18.7 | 20.3 | 23 | 23.5 | 23.3 | 23 | 22.8 | 22.5 |
| Lavínia-SP | 352650 | 3.9 | 3.9 | 3.5 | 3.3 | 3.7 | 4.6 | 4.7 | 4 | 3.9 | 3.5 | 4 |
| Lavrinhas-SP | 352660 | 13.6 | 13.5 | 16.1 | 17.2 | 16.6 | 18.1 | 18.1 | 16.3 | 15.4 | 13.7 | 15.5 |
| Leme-SP | 352670 | 31.6 | 30.7 | 31.6 | 33.9 | 34.7 | 35.3 | 35.3 | 35.7 | 31.4 | 31.7 | 31.1 |
| Lençóis Paulista-SP | 352680 | 37.2 | 34 | 36 | 37 | 34 | 35.6 | 35.6 | 39.1 | 36.1 | 35.6 | 27.8 |
| Limeira-SP | 352690 | 40.7 | 42.4 | 44.6 | 45.5 | 47.2 | 48.4 | 47.9 | 46 | 45 | 44.4 | 44.7 |
| Lindóia-SP | 352700 | 6.6 | 7.7 | 9 | 8.9 | 8.7 | 8.4 | 8.8 | 9.4 | 8.7 | 8.9 | 9 |
| Lins-SP | 352710 | 52.5 | 53.3 | 54.1 | 52 | 52.5 | 51.6 | 50.4 | 50.1 | 47.2 | 44.8 | 43.8 |
| Lorena-SP | 352720 | 26.4 | 25.2 | 25 | 24.7 | 26.3 | 28.4 | 29 | 27.3 | 25.3 | 24.5 | 24.1 |
| Lourdes-SP | 352725 | 11.3 | 14.1 | 17 | 19.6 | 20.4 | 23.3 | 23.7 | 33 | 22.6 | 17.2 | 11 |
| Louveira-SP | 352730 | 44 | 44.4 | 48.7 | 45.7 | 47.9 | 53 | 49.8 | 45.3 | 41.4 | 40.2 | 39.3 |
| Lucélia-SP | 352740 | 6.3 | 6.4 | 6.3 | 6.6 | 7 | 7.2 | 7.3 | 7.3 | 7.6 | 7.7 | 7.7 |
| Lucianópolis-SP | 352750 | 4.6 | 4.1 | 4.6 | 4.3 | 5.5 | 6.4 | 6.4 | 6.7 | 7 | 7.1 | 7 |
| Luís Antônio-SP | 352760 | 14.8 | 22.9 | 17.9 | 31.9 | 35.4 | 40.9 | 54.7 | 38.9 | 36 | 34.5 | 32.5 |
| Luiziânia-SP | 352770 | 3.4 | 3.5 | 3.6 | 6.8 | 7.1 | 7.4 | 7.1 | 6.9 | 4.1 | 3.6 | 3.4 |
| Lupércio-SP | 352780 | 4.9 | 4.9 | 5.3 | 5.9 | 7.8 | 8.3 | 8.6 | 8.2 | 8.5 | 7.5 | 7.5 |
| Lutécia-SP | 352790 | 3.6 | 3.5 | 3.8 | 4.8 | 4.8 | 5.4 | 6.7 | 7.2 | 7.4 | 7.4 | 7.4 |
| Macatuba-SP | 352800 | 15.1 | 16.3 | 20.8 | 20.4 | 18.5 | 21.6 | 22 | 22.2 | 22.2 | 22.3 | 16.7 |
| Macaubal-SP | 352810 | 15 | 20 | 20.2 | 21.8 | 24.9 | 28.7 | 30.3 | 25.5 | 24.7 | 24.3 | 22 |
| Macedônia-SP | 352820 | 5 | 7.1 | 7.5 | 5.7 | 6.3 | 7.4 | 7.7 | 6.7 | 6.7 | 6.7 | 7 |
| Magda-SP | 352830 | 12.5 | 13.1 | 14.9 | 15.3 | 16.7 | 18.4 | 19.4 | 17.7 | 19.9 | 20.8 | 17.7 |
| Mairinque-SP | 352840 | 35.8 | 34.4 | 37.7 | 40.6 | 42.5 | 44.2 | 45.4 | 43.5 | 43.7 | 38.2 | 36.7 |
| Mairiporã-SP | 352850 | 23.1 | 22.5 | 23.7 | 24.5 | 24.7 | 24 | 24.1 | 23.3 | 21.2 | 20.2 | 18.9 |
| Manduri-SP | 352860 | 4.2 | 5.5 | 5.1 | 5.8 | 5.8 | 6.2 | 6.7 | 6.6 | 7.2 | 8.1 | 7.9 |
| Marabá Paulista-SP | 352870 | 2.9 | 3.4 | 4.5 | 5.3 | 5.2 | 5.2 | 4.7 | 4.4 | 4.5 | 4.1 | 3.7 |
| Maracaí-SP | 352880 | 4.3 | 5.3 | 7.8 | 8.6 | 9.2 | 10 | 10.5 | 11.3 | 13.7 | 14 | 14.7 |
| Marapoama-SP | 352885 | 13.1 | 8.8 | 10.3 | 12.1 | 16.2 | 15.7 | 16.1 | 17.9 | 17.4 | 17.8 | 17.7 |
| Mariápolis-SP | 352890 | 2.5 | 2.6 | 2.6 | 2.5 | 2.7 | 3 | 2.8 | 2.6 | 2.7 | 3.1 | 3.4 |
| Marília-SP | 352900 | 31.1 | 31.2 | 33 | 33.3 | 34.9 | 36.7 | 37.1 | 34.4 | 33.6 | 33.1 | 32.9 |
| Marinópolis-SP | 352910 | 2.4 | 2.8 | 3.4 | 3.3 | 2.4 | 1.9 | 2.1 | 2.2 | 2 | 2.7 | 2.6 |
| Martinópolis-SP | 352920 | 11.3 | 11.6 | 11.7 | 12.2 | 12.7 | 12.8 | 13 | 13.7 | 13.7 | 13.5 | 13.4 |
| Matão-SP | 352930 | 46.3 | 46.2 | 43.4 | 44.7 | 45.5 | 46.7 | 43.7 | 42 | 40.7 | 40.8 | 40.3 |
| Mauá-SP | 352940 | 41.6 | 41.4 | 39.9 | 40.6 | 41.6 | 44.8 | 43.8 | 41.6 | 40.4 | 38.4 | 38.5 |
| Mendonça-SP | 352950 | 13.4 | 13.3 | 13.4 | 14.9 | 17.9 | 20.8 | 19.3 | 19.7 | 20.3 | 21.3 | 22.2 |
| Meridiano-SP | 352960 | 11.6 | 15.1 | 15.6 | 20.6 | 17.3 | 17.5 | 16.1 | 15.5 | 15.9 | 16.4 | 16.7 |
| Mesópolis-SP | 352965 | 3.6 | 5.6 | 6.3 | 8.1 | 13.4 | 13.3 | 14.2 | 15.7 | 15.5 | 17.6 | 19.6 |
| Miguelópolis-SP | 352970 | 11.8 | 12.9 | 14.1 | 15 | 21.1 | 23.6 | 22.8 | 22.5 | 25.5 | 25.9 | 28.1 |
| Mineiros do Tietê-SP | 352980 | 5.5 | 5.6 | 5.9 | 5.7 | 6.4 | 7.1 | 7.5 | 6.9 | 6.3 | 6.3 | 10.7 |
| Miracatu-SP | 352990 | 4.1 | 5.1 | 5.4 | 5.5 | 6.4 | 5.4 | 5.6 | 5.5 | 5.2 | 5.6 | 5.3 |
| Mira Estrela-SP | 353000 | 6.1 | 6.7 | 8.3 | 8 | 8.4 | 8.6 | 8.9 | 8.7 | 8.1 | 8.6 | 7.6 |
| Mirandópolis-SP | 353010 | 8.9 | 9.1 | 9.3 | 9 | 10.1 | 10.8 | 11 | 10.6 | 10.3 | 10 | 13.6 |
| Mirante do Paranapanema-SP | 353020 | 8 | 11.7 | 19.5 | 21 | 22.4 | 26.4 | 26.2 | 24.5 | 22.3 | 21.2 | 20.6 |
| Mirassol-SP | 353030 | 32.5 | 33.1 | 35.3 | 35.6 | 37.7 | 38.3 | 38.8 | 38.1 | 36.6 | 35.4 | 34.9 |
| Mirassolândia-SP | 353040 | 9.7 | 10.1 | 10.7 | 10.8 | 11.6 | 12 | 13.8 | 14 | 13.6 | 13.2 | 13 |
| Mococa-SP | 353050 | 35 | 27.9 | 28.2 | 30.8 | 31.3 | 31.7 | 32.5 | 29.9 | 29.9 | 31.9 | 31.4 |
| Mogi das Cruzes-SP | 353060 | 36.8 | 38.5 | 41.4 | 35.6 | 35.1 | 36.8 | 36.4 | 34.5 | 32.5 | 31.5 | 30.9 |
| Mogi Guaçu-SP | 353070 | 41.6 | 41.2 | 41.4 | 42.8 | 37.3 | 44.2 | 43.8 | 42.1 | 39.5 | 38.7 | 37.1 |
| Mogi Mirim-SP | 353080 | 33.4 | 34 | 33.8 | 35.3 | 37.1 | 38.2 | 37.9 | 36.8 | 34.8 | 34.4 | 34.1 |
| Mombuca-SP | 353090 | 18.4 | 16.6 | 16.4 | 14.8 | 16 | 22.9 | 22.4 | 22.4 | 21.4 | 24.2 | 24.4 |
| Monções-SP | 353100 | 18.7 | 19.4 | 23.2 | 28.8 | 33.8 | 43.4 | 37.7 | 32.9 | 32.4 | 31.5 | 14.9 |
| Mongaguá-SP | 353110 | 13 | 12.7 | 12.8 | 12.7 | 13.2 | 14 | 14.7 | 15.3 | 15.2 | 15.1 | 14.2 |
| Monte Alegre do Sul-SP | 353120 | 17.2 | 17.2 | 20.3 | 23 | 22.5 | 25.3 | 25.9 | 29.1 | 30.6 | 32.6 | 34.3 |
| Monte Alto-SP | 353130 | 48.2 | 50.5 | 48.8 | 48.5 | 49.2 | 47.5 | 45.9 | 42.9 | 42.5 | 41.7 | 42.8 |
| Monte Aprazível-SP | 353140 | 26.8 | 29.1 | 29.7 | 30.8 | 33.9 | 36.1 | 45 | 36.3 | 32 | 31.5 | 31.6 |
| Monte Azul Paulista-SP | 353150 | 27.7 | 29 | 30.5 | 31.6 | 29.9 | 30.4 | 30.6 | 30.3 | 29.7 | 26.1 | 28.2 |
| Monte Castelo-SP | 353160 | 9.8 | 13.2 | 15.2 | 15.6 | 18.6 | 19.9 | 20.9 | 17.8 | 17.9 | 18.2 | 17.9 |
| Monteiro Lobato-SP | 353170 | 9.2 | 11 | 12.1 | 12.7 | 13 | 13.1 | 13.4 | 12.1 | 11.5 | 11.7 | 11.7 |
| Monte Mor-SP | 353180 | 25.4 | 25.2 | 28.8 | 29.7 | 32.2 | 33.7 | 35.7 | 32 | 30.1 | 29.6 | 29.3 |
| Morro Agudo-SP | 353190 | 30.8 | 25.4 | 31.6 | 28.5 | 33.2 | 32 | 33.6 | 35.4 | 33.4 | 32.1 | 30.4 |
| Morungaba-SP | 353200 | 30.3 | 33.3 | 30.2 | 30.7 | 31.4 | 33.3 | 32.4 | 28.4 | 27.2 | 28 | 27.2 |
| Motuca-SP | 353205 | 41.3 | 39.3 | 39.6 | 40 | 38.7 | 37.7 | 37.1 | 37.6 | 37.2 | 35.6 | 33.9 |
| Murutinga do Sul-SP | 353210 | 8 | 7.9 | 7.7 | 7.6 | 8.1 | 9 | 8.6 | 9 | 9.5 | 10.2 | 10.2 |
| Nantes-SP | 353215 | 4.8 | 5 | 6 | 6.5 | 6.4 | 7.3 | 7 | 7.5 | 7.9 | 8.5 | 8.4 |
| Narandiba-SP | 353220 | 21 | 22.7 | 20.9 | 24.2 | 21.2 | 15 | 15.4 | 14.3 | 14 | 13.7 | 12.9 |
| Natividade da Serra-SP | 353230 | 3.7 | 3.8 | 4.4 | 4.9 | 5.3 | 5.5 | 5.3 | 4.9 | 4.6 | 4.8 | 4.7 |
| Nazaré Paulista-SP | 353240 | 8.5 | 10.7 | 13.4 | 12.4 | 13.8 | 14.1 | 15.5 | 15.9 | 15.3 | 14.9 | 14.8 |
| Neves Paulista-SP | 353250 | 26.3 | 26.1 | 26.6 | 26.6 | 27.9 | 28.8 | 29.7 | 29.5 | 28.7 | 29.1 | 29.2 |
| Nhandeara-SP | 353260 | 21.3 | 23.6 | 23.8 | 24.2 | 24.8 | 26.2 | 27 | 25.2 | 25.6 | 24.9 | 23 |
| Nipoã-SP | 353270 | 13.8 | 15.2 | 16.5 | 17.6 | 23.4 | 28.6 | 47.8 | 29 | 15.8 | 18.6 | 18.5 |
| Nova Aliança-SP | 353280 | 14.9 | 14.7 | 15.8 | 17.5 | 18.9 | 20 | 20.8 | 20.3 | 20 | 20.1 | 19.3 |
| Nova Campina-SP | 353282 | 6.8 | 6.8 | 7.5 | 9.2 | 9.3 | 9.7 | 9.5 | 8 | 6 | 8 | 7.7 |
| Nova Canaã Paulista-SP | 353284 | 1.3 | 1.7 | 1.9 | 2.2 | 2 | 2.3 | 4 | 4.5 | 5 | 5.9 | 5.4 |
| Nova Castilho-SP | 353286 | 10.1 | 11.4 | 10.3 | 7.9 | 9.1 | 10.7 | 9.4 | 11.8 | 14.5 | 15.8 | 10.8 |
| Nova Europa-SP | 353290 | 45.7 | 44.7 | 42.3 | 41.7 | 42.4 | 44.3 | 42.7 | 41.8 | 40.3 | 39.8 | 38.1 |
| Nova Granada-SP | 353300 | 17.7 | 18.9 | 21.9 | 22.9 | 24.2 | 25.4 | 25.4 | 24.4 | 24.3 | 25 | 24.4 |
| Nova Guataporanga-SP | 353310 | 5.7 | 8 | 9.1 | 9.2 | 12.8 | 15 | 15 | 13.8 | 14.5 | 12.7 | 14 |
| Nova Independência-SP | 353320 | 19.8 | 28.8 | 35.5 | 26.8 | 33.8 | 33.7 | 34.5 | 32.1 | 32.1 | 30.6 | 30.3 |
| Novais-SP | 353325 | 10.4 | 12.6 | 14.3 | 18.9 | 25.8 | 22.9 | 23.3 | 18.9 | 17.8 | 17.7 | 16.8 |
| Nova Luzitânia-SP | 353330 | 8.7 | 12.2 | 11.9 | 14.8 | 16 | 18.9 | 19 | 18.7 | 20.4 | 20.1 | 11.9 |
| Nova Odessa-SP | 353340 | 56.7 | 59 | 57.4 | 52.6 | 51.3 | 39.4 | 40.4 | 39.3 | 37.8 | 37.7 | 37.4 |
| Novo Horizonte-SP | 353350 | 18 | 20.6 | 21.5 | 21.3 | 21 | 23.2 | 25.9 | 28.6 | 28 | 27.4 | 27.7 |
| Nuporanga-SP | 353360 | 34.1 | 34.5 | 35 | 36.3 | 36.2 | 36.2 | 35.2 | 39.2 | 40.6 | 40 | 38.6 |
| Ocauçu-SP | 353370 | 6.3 | 7.2 | 7.8 | 8.5 | 11.5 | 11.1 | 10.8 | 10.6 | 10.5 | 9.4 | 9.1 |
| Óleo-SP | 353380 | 3 | 3.1 | 3.3 | 3.9 | 3.7 | 3.4 | 4.4 | 4.5 | 4.2 | 3.7 | 3.6 |
| Olímpia-SP | 353390 | 30.9 | 32.3 | 33.3 | 33.3 | 34.2 | 36.2 | 36.8 | 36.4 | 35.7 | 36.6 | 36.4 |
| Onda Verde-SP | 353400 | 22.3 | 15.1 | 18.8 | 19.8 | 21.3 | 22.6 | 23.4 | 32.5 | 29.9 | 28.6 | 27.5 |
| Oriente-SP | 353410 | 27.9 | 25.7 | 28.3 | 27.9 | 28.8 | 31.9 | 29.8 | 26.9 | 25.6 | 27.1 | 27.6 |
| Orindiúva-SP | 353420 | 79.5 | 76.9 | 77.2 | 73.5 | 69.3 | 48.3 | 54.6 | 53.2 | 50.6 | 42.6 | 37.5 |
| Orlândia-SP | 353430 | 55.1 | 53.8 | 56.8 | 51.5 | 52.2 | 50.8 | 50.6 | 48.3 | 46.7 | 45.1 | 43.8 |
| Osasco-SP | 353440 | 48 | 48.1 | 48.6 | 48.7 | 48.7 | 50 | 50.2 | 48.2 | 46 | 45.8 | 45 |
| Oscar Bressane-SP | 353450 | 8.1 | 8.5 | 9.1 | 8.7 | 9.2 | 9 | 10.5 | 9.8 | 8.8 | 9 | 8.8 |
| Osvaldo Cruz-SP | 353460 | 21.2 | 22.1 | 22.8 | 22.3 | 23 | 20.3 | 20.8 | 21.1 | 20.8 | 20.5 | 20 |
| Ourinhos-SP | 353470 | 17.4 | 18.6 | 19.5 | 19.6 | 20.2 | 21 | 21.7 | 20.8 | 20.5 | 18.9 | 18.8 |
| Ouroeste-SP | 353475 | 8.6 | 10.2 | 15.1 | 19.4 | 21.1 | 19.8 | 22.4 | 20.9 | 20.2 | 19.9 | 17.2 |
| Ouro Verde-SP | 353480 | 5.5 | 6 | 6.7 | 5.2 | 5.4 | 6.7 | 7.1 | 6.1 | 5.8 | 7.2 | 8 |
| Pacaembu-SP | 353490 | 5.4 | 5.4 | 5.4 | 5.4 | 6.2 | 6.5 | 7.4 | 7.7 | 7.6 | 8.3 | 8.3 |
| Palestina-SP | 353500 | 17.4 | 21.5 | 26.3 | 27.6 | 30.3 | 33.6 | 31.7 | 31.5 | 29.4 | 31.4 | 29.3 |
| Palmares Paulista-SP | 353510 | 38.8 | 44.8 | 41.3 | 42.1 | 42.9 | 44.2 | 30.6 | 30.8 | 29.1 | 30.1 | 31.2 |
| Palmeira d'Oeste-SP | 353520 | 4.5 | 5.6 | 5.7 | 6.2 | 6.7 | 6.5 | 7.3 | 7.6 | 7.8 | 8.1 | 8.4 |
| Palmital-SP | 353530 | 6.8 | 6.8 | 7.1 | 7.4 | 7.8 | 8.6 | 8.6 | 15.3 | 16.1 | 16.4 | 16.7 |
| Panorama-SP | 353540 | 5.5 | 5.6 | 6.1 | 6.3 | 6.1 | 6.4 | 6.2 | 5.9 | 5.7 | 5.6 | 5.4 |
| Paraguaçu Paulista-SP | 353550 | 10 | 11 | 11.3 | 11.3 | 11.3 | 11.7 | 11.5 | 11.9 | 11.9 | 12.2 | 12.5 |
| Paraibuna-SP | 353560 | 14.6 | 16.9 | 17.1 | 17.8 | 17.4 | 18.1 | 21.2 | 18.4 | 18 | 17.4 | 17 |
| Paraíso-SP | 353570 | 26.5 | 29.3 | 31.2 | 32.6 | 36.8 | 37.3 | 35.7 | 35.9 | 35 | 36.3 | 35.7 |
| Paranapanema-SP | 353580 | 7.1 | 7.6 | 7.6 | 7.9 | 8.3 | 7.9 | 9 | 9.1 | 8.9 | 9 | 8.7 |
| Paranapuã-SP | 353590 | 4 | 6.1 | 7.8 | 8.5 | 10.6 | 10.9 | 11.8 | 12.2 | 10.6 | 10.3 | 9.8 |
| Parapuã-SP | 353600 | 18.3 | 17.6 | 19.6 | 9.3 | 9.8 | 7.7 | 8.2 | 8.7 | 8.6 | 8.9 | 8.7 |
| Pardinho-SP | 353610 | 5.2 | 5.4 | 6.4 | 7.5 | 9.6 | 12 | 13.5 | 14.4 | 12.7 | 13.1 | 12.8 |
| Pariquera-SPAçu-SP | 353620 | 5.9 | 5.9 | 6.5 | 6.2 | 6.6 | 6.5 | 6.6 | 6 | 5.9 | 5.9 | 6 |
| Parisi-SP | 353625 | 10.1 | 11.9 | 15 | 13.9 | 13.8 | 15 | 15.3 | 13.9 | 14.8 | 15.3 | 14.7 |
| Patrocínio Paulista-SP | 353630 | 16.7 | 17.2 | 18.8 | 19.2 | 19.2 | 20.1 | 20.2 | 20.4 | 19.5 | 19.5 | 19.2 |
| Paulicéia-SP | 353640 | 3.2 | 3.7 | 3.5 | 3.6 | 3.9 | 4.1 | 5 | 5.4 | 5.1 | 5.3 | 5 |
| Paulínia-SP | 353650 | 41.6 | 40.4 | 41.7 | 46 | 51 | 47.7 | 48.5 | 48.4 | 44.7 | 43.2 | 43.7 |
| Paulistânia-SP | 353657 | 1.7 | 2.1 | 2.4 | 2.1 | 2.1 | 2.3 | 2.8 | 3.1 | 3.3 | 4.1 | 4.1 |
| Paulo de Faria-SP | 353660 | 15.3 | 19.6 | 23.3 | 25.5 | 26.9 | 26.3 | 24.9 | 25.2 | 25.2 | 27.7 | 25.1 |
| Pederneiras-SP | 353670 | 24.6 | 25.7 | 30.3 | 30.6 | 31.9 | 33 | 33.4 | 31.1 | 28.6 | 27.1 | 26.3 |
| Pedra Bela-SP | 353680 | 6.7 | 7.1 | 7.5 | 9.3 | 10.3 | 10.7 | 11.3 | 12.5 | 12.5 | 11.6 | 10 |
| Pedranópolis-SP | 353690 | 14.1 | 15.4 | 15.9 | 14.2 | 14.2 | 15.5 | 15.6 | 14.6 | 14 | 13 | 11.9 |
| Pedregulho-SP | 353700 | 19.8 | 20.9 | 21.7 | 22.5 | 21.2 | 22 | 22.4 | 22.1 | 21.9 | 21.4 | 20.9 |
| Pedreira-SP | 353710 | 27.3 | 28 | 28.7 | 28.4 | 28.1 | 28 | 28.6 | 28.5 | 28.3 | 27 | 26 |
| Pedrinhas Paulista-SP | 353715 | 8.8 | 9 | 9.4 | 9.5 | 10 | 10.7 | 10.9 | 10.5 | 10.9 | 11.2 | 11.6 |
| Pedro de Toledo-SP | 353720 | 8.4 | 6.8 | 6.5 | 6 | 5.3 | 5.5 | 5.4 | 4.9 | 4.6 | 4.5 | 4 |
| Penápolis-SP | 353730 | 17.2 | 17.4 | 17.7 | 20.1 | 19.6 | 19.9 | 19.8 | 21.5 | 18.3 | 17.5 | 16.7 |
| Pereira Barreto-SP | 353740 | 16.3 | 17.2 | 18 | 19 | 23.1 | 21.4 | 22.7 | 21.7 | 21 | 21 | 19.8 |
| Pereiras-SP | 353750 | 3.3 | 3.2 | 3.2 | 3.5 | 3.8 | 4.3 | 4.6 | 4.7 | 4.6 | 4.5 | 4.3 |
| Peruíbe-SP | 353760 | 17.3 | 16 | 16.3 | 15.6 | 15.3 | 15.4 | 15.9 | 17 | 16.9 | 16.9 | 16.1 |
| Piacatu-SP | 353770 | 9 | 13.4 | 18.4 | 30.3 | 8.1 | 8 | 8.9 | 9.2 | 8.8 | 9 | 9.1 |
| Piedade-SP | 353780 | 8 | 8.6 | 9 | 9.5 | 10.5 | 11.7 | 12.9 | 12.8 | 12.6 | 12 | 11.1 |
| Pilar do Sul-SP | 353790 | 4.9 | 5 | 6.6 | 7.4 | 8.4 | 11.5 | 11.8 | 12.1 | 11.6 | 11.6 | 11.2 |
| Pindamonhangaba-SP | 353800 | 28.6 | 28.4 | 31.4 | 32.8 | 30.9 | 32.1 | 31.1 | 30.4 | 27 | 26.8 | 26.9 |
| Pindorama-SP | 353810 | 26.1 | 26.9 | 27 | 29.2 | 32.6 | 31.6 | 37 | 39.5 | 38.7 | 31.3 | 31.8 |
| Pinhalzinho-SP | 353820 | 16 | 18 | 18.6 | 19.2 | 20.5 | 19.9 | 18 | 18.8 | 18.5 | 18.4 | 15.7 |
| Piquerobi-SP | 353830 | 9.2 | 9.1 | 8.8 | 8.5 | 8.5 | 8.8 | 9.2 | 8.8 | 9.6 | 9.8 | 9.9 |
| Piquete-SP | 353850 | 16.3 | 16.6 | 16.6 | 16 | 16.3 | 17.1 | 17.7 | 17.5 | 15.1 | 13 | 12.5 |
| Piracaia-SP | 353860 | 20.5 | 21.1 | 22.2 | 22.4 | 23.3 | 23.8 | 22.8 | 22.7 | 21.8 | 20.3 | 19.9 |
| Piracicaba-SP | 353870 | 49.6 | 49.1 | 51.2 | 51.4 | 53.8 | 56.5 | 55.8 | 52.7 | 50.5 | 50.9 | 51.2 |
| Piraju-SP | 353880 | 9.7 | 9.9 | 9.9 | 10 | 10.1 | 10.5 | 10.6 | 10.5 | 10.2 | 9.3 | 9.7 |
| Pirajuí-SP | 353890 | 17.4 | 17.2 | 17.6 | 17.5 | 18.8 | 18.9 | 19.3 | 19.2 | 19 | 19.5 | 18.4 |
| Pirangi-SP | 353900 | 16.4 | 17.3 | 17.9 | 19 | 19.3 | 19.5 | 19.2 | 19.2 | 18.4 | 19.5 | 20 |
| Pirapora do Bom Jesus-SP | 353910 | 15.7 | 26.3 | 32 | 26.6 | 22.6 | 19.9 | 17.1 | 15.8 | 15.4 | 14.5 | 14.5 |
| Pirapozinho-SP | 353920 | 20.6 | 21.8 | 21.1 | 21.8 | 22.1 | 20.9 | 20.9 | 20.8 | 20.7 | 20.5 | 20.3 |
| Pirassununga-SP | 353930 | 30.5 | 31.1 | 36 | 35.6 | 36.7 | 36.9 | 36.8 | 35 | 34.4 | 32.8 | 34.3 |
| Piratininga-SP | 353940 | 20 | 21.5 | 21.8 | 21.1 | 22.1 | 24.3 | 26.3 | 26 | 25.5 | 25.1 | 25.2 |
| Pitangueiras-SP | 353950 | 35.4 | 39.4 | 42.5 | 34.8 | 36 | 37.7 | 39.7 | 38.4 | 30.8 | 29.3 | 29 |
| Planalto-SP | 353960 | 14.1 | 16.2 | 14.9 | 16.1 | 19.8 | 24.1 | 29.4 | 25.8 | 92.3 | 77.8 | 71.9 |
| Platina-SP | 353970 | 9.4 | 9.3 | 10.5 | 8.8 | 8.1 | 8.9 | 7.3 | 7.7 | 6.5 | 8.3 | 9.1 |
| Poá-SP | 353980 | 29.5 | 27.1 | 27.3 | 27.3 | 26.8 | 27.9 | 28.3 | 26.5 | 25.5 | 25 | 24.4 |
| Poloni-SP | 353990 | 19.9 | 21.5 | 23.6 | 23.8 | 25.1 | 26 | 29.6 | 24.2 | 22.6 | 22.7 | 21.3 |
| Pompéia-SP | 354000 | 44.3 | 41.6 | 41.4 | 39.5 | 44 | 45.5 | 45 | 44.6 | 44 | 44 | 42.9 |
| Pongaí-SP | 354010 | 17.1 | 17.5 | 19 | 19.4 | 19.5 | 20 | 20.3 | 20.6 | 20.3 | 20 | 20.8 |
| Pontal-SP | 354020 | 33.2 | 38.3 | 37.6 | 30.6 | 42.6 | 39 | 35.5 | 34.7 | 35.2 | 35.4 | 34.1 |
| Pontalinda-SP | 354025 | 1.8 | 2.1 | 3 | 4.4 | 4.4 | 5.9 | 8 | 4.6 | 4.1 | 3.4 | 3.1 |
| Pontes Gestal-SP | 354030 | 27.7 | 25.5 | 25.8 | 28.2 | 28.4 | 24.3 | 24.5 | 24.2 | 24.5 | 24.6 | 20 |
| Populina-SP | 354040 | 4.2 | 5.2 | 8.2 | 8.1 | 10.6 | 10.7 | 12.5 | 13.6 | 13.2 | 22.3 | 20.7 |
| Porangaba-SP | 354050 | 5.2 | 5.4 | 5.4 | 5.8 | 5.6 | 5.9 | 6 | 6.4 | 6.7 | 6.6 | 6.3 |
| Porto Feliz-SP | 354060 | 21.7 | 23.7 | 25.1 | 25.2 | 27.3 | 28.3 | 29.3 | 28.9 | 27.8 | 27.4 | 25.8 |
| Porto Ferreira-SP | 354070 | 46.3 | 47.7 | 43.7 | 44.4 | 45.5 | 43.3 | 43.1 | 42.3 | 39.2 | 39.6 | 39 |
| Potim-SP | 354075 | 4 | 4 | 4.4 | 4.5 | 5.3 | 7.4 | 7.7 | 7.1 | 6.7 | 5.8 | 5.6 |
| Potirendaba-SP | 354080 | 30.1 | 31.2 | 35.3 | 34.7 | 39 | 38.7 | 39.7 | 38.2 | 37.9 | 36.4 | 37.6 |
| Pracinha-SP | 354085 | 0.6 | 0.7 | 0.9 | 4.2 | 3.2 | 2.3 | 1.4 | 1.4 | 1.1 | 1.2 | 1.2 |
| Pradópolis-SP | 354090 | 63.4 | 60.6 | 51 | 47.4 | 48 | 49.1 | 49.1 | 44.9 | 44.2 | 43.8 | 42.1 |
| Praia Grande-SP | 354100 | 28.6 | 28.2 | 29.9 | 30.4 | 30.6 | 31.6 | 32.1 | 32.4 | 31.1 | 30.9 | 30.3 |
| Pratânia-SP | 354105 | 7 | 8.3 | 8.1 | 8.1 | 9 | 9.5 | 9 | 7.9 | 7.8 | 7.7 | 7.6 |
| Presidente Alves-SP | 354110 | 7.1 | 7.3 | 9.1 | 8.8 | 10.6 | 10.5 | 10.4 | 9.9 | 11 | 12.3 | 11.9 |
| Presidente Bernardes-SP | 354120 | 17 | 18.6 | 16.2 | 19.6 | 18 | 19.2 | 20.1 | 20.6 | 20.9 | 21.5 | 21.9 |
| Presidente Epitácio-SP | 354130 | 15 | 14.9 | 14.8 | 13.8 | 13.6 | 14.6 | 15.1 | 15.1 | 14.5 | 14.5 | 14.3 |
| Presidente Prudente-SP | 354140 | 34.2 | 35 | 36.1 | 35.6 | 35.9 | 36 | 36.8 | 37.3 | 37.4 | 37.2 | 37.3 |
| Presidente Venceslau-SP | 354150 | 19 | 19.1 | 19.7 | 19.8 | 19.9 | 20.7 | 21.1 | 20.4 | 20.1 | 20 | 19.9 |
| Promissão-SP | 354160 | 21.7 | 25.2 | 26.7 | 30.2 | 30 | 29.7 | 29.6 | 35.8 | 27.6 | 25.1 | 23.5 |
| Quadra-SP | 354165 | 2.6 | 2.8 | 3.4 | 3.8 | 4.1 | 5.3 | 5.1 | 5.5 | 5.8 | 5.6 | 6 |
| Quatá-SP | 354170 | 31.2 | 33.3 | 33.8 | 32.8 | 30.8 | 30 | 27.6 | 28.7 | 27.6 | 27.7 | 32.6 |
| Queiroz-SP | 354180 | 1.5 | 2 | 1.9 | 1.8 | 2.3 | 3.2 | 3.3 | 3.2 | 2.8 | 3 | 2.9 |
| Queluz-SP | 354190 | 9.4 | 8.8 | 9.4 | 9.7 | 9.2 | 10.1 | 11.5 | 10.3 | 8.7 | 6.2 | 6.5 |
| Quintana-SP | 354200 | 25.1 | 22.3 | 24.7 | 23.5 | 23.6 | 24.6 | 23.5 | 22.2 | 21.8 | 22.9 | 23 |
| Rafard-SP | 354210 | 30.9 | 31.8 | 33.2 | 31.9 | 33.4 | 37.1 | 36.3 | 34.6 | 33.8 | 35.8 | 36.9 |
| Rancharia-SP | 354220 | 16 | 16.5 | 17.2 | 17.2 | 17.3 | 18 | 18.3 | 18.6 | 18.5 | 18.9 | 19.7 |
| Redenção da Serra-SP | 354230 | 5 | 5.7 | 6.8 | 7.2 | 7.4 | 7.4 | 7.9 | 6.5 | 6 | 5.9 | 6.8 |
| Regente Feijó-SP | 354240 | 19.4 | 20.4 | 21.3 | 22.3 | 23 | 23.4 | 26.2 | 25 | 24.3 | 24.1 | 24.7 |
| Reginópolis-SP | 354250 | 9 | 8.9 | 9.1 | 8 | 8.3 | 8.9 | 9.2 | 9 | 10.6 | 14.5 | 11 |
| Registro-SP | 354260 | 18.2 | 18.8 | 19.5 | 19.4 | 20.2 | 19.7 | 19.7 | 18.8 | 18.7 | 18.3 | 17.9 |
| Restinga-SP | 354270 | 15.6 | 16.5 | 16.5 | 17.4 | 16.7 | 17.2 | 18.3 | 17.9 | 17.7 | 17.4 | 16.4 |
| Ribeira-SP | 354280 | 2.9 | 3 | 3 | 3 | 2.6 | 2.4 | 2.7 | 2.7 | 3.6 | 4.2 | 4.2 |
| Ribeirão Bonito-SP | 354290 | 13 | 13.8 | 13.8 | 13.6 | 14.7 | 15.6 | 15.4 | 15.5 | 15.8 | 15.8 | 15.8 |
| Ribeirão Branco-SP | 354300 | 2 | 1.9 | 1.9 | 2.2 | 3 | 3.8 | 4.2 | 2.8 | 2.4 | 2.9 | 3 |
| Ribeirão Corrente-SP | 354310 | 9.8 | 9.5 | 9.6 | 9.9 | 10.1 | 10.4 | 10.8 | 11.2 | 11.5 | 11.7 | 11.7 |
| Ribeirão do Sul-SP | 354320 | 3.3 | 3.5 | 3.4 | 3.8 | 4.3 | 7.1 | 7.5 | 7.5 | 8.3 | 8.3 | 9.1 |
| Ribeirão dos Índios-SP | 354323 | 6.5 | 6.7 | 6.3 | 6.3 | 7 | 6.5 | 7 | 7.1 | 7.5 | 7.6 | 8.5 |
| Ribeirão Grande-SP | 354325 | 5.3 | 6.8 | 7.1 | 7.5 | 10.2 | 11 | 10.8 | 8.1 | 4.8 | 4.4 | 4.5 |
| Ribeirão Pires-SP | 354330 | 49.1 | 48 | 48.2 | 48.7 | 47.8 | 49.1 | 49.7 | 47 | 47.2 | 46.5 | 45.8 |
| Ribeirão Preto-SP | 354340 | 37.1 | 40.3 | 42.4 | 41.9 | 42.6 | 43 | 43.5 | 43.3 | 42.4 | 41.8 | 41.4 |
| Riversul-SP | 354350 | 1.5 | 1.5 | 1.8 | 2.4 | 2.7 | 2.4 | 2.5 | 2.7 | 2.5 | 2.3 | 2.4 |
| Rifaina-SP | 354360 | 19.5 | 21.4 | 23.7 | 23.9 | 21.1 | 21.4 | 22.8 | 23.9 | 22.3 | 22.2 | 21 |
| Rincão-SP | 354370 | 37.5 | 33.2 | 34.8 | 37.8 | 38.5 | 38.5 | 34.7 | 33.6 | 31.8 | 30.1 | 29.6 |
| Rinópolis-SP | 354380 | 4.5 | 4.8 | 5.6 | 6.2 | 6.7 | 6 | 6.4 | 6.4 | 6.4 | 6.6 | 6.3 |
| Rio Claro-SP | 354390 | 40.9 | 39.6 | 43 | 43.9 | 45.6 | 46.8 | 47.2 | 44.2 | 43.7 | 43.9 | 44.2 |
| Rio das Pedras-SP | 354400 | 31.9 | 29.8 | 32.1 | 32.4 | 35.4 | 40.2 | 39.3 | 42.7 | 41.3 | 41.6 | 42.3 |
| Rio Grande da Serra-SP | 354410 | 29.9 | 30.5 | 31.9 | 33.4 | 32.2 | 33.3 | 33.3 | 33.3 | 30.3 | 29.2 | 28.8 |
| Riolândia-SP | 354420 | 5.3 | 9 | 14.7 | 18.8 | 19.4 | 17.7 | 20.5 | 19.2 | 17 | 16.4 | 12.9 |
| Rosana-SP | 354425 | 6.9 | 7.4 | 7.6 | 7.5 | 9.5 | 15.5 | 11.5 | 7.3 | 6.8 | 6.7 | 6.7 |
| Roseira-SP | 354430 | 11.8 | 12.1 | 13.1 | 14 | 14.4 | 17.5 | 17 | 16.4 | 15.8 | 15.3 | 14.8 |
| Rubiácea-SP | 354440 | 2.1 | 2.7 | 5.8 | 6.7 | 8.8 | 10.5 | 10.6 | 11.8 | 10.5 | 14.4 | 18.8 |
| Rubinéia-SP | 354450 | 4.9 | 5.9 | 6 | 5.9 | 6.1 | 5.6 | 5.7 | 6.3 | 6.3 | 6 | 6.8 |
| Sabino-SP | 354460 | 33.7 | 34 | 32.9 | 31.3 | 31.9 | 31.1 | 31.2 | 29.6 | 28.9 | 27.6 | 26.7 |
| Sagres-SP | 354470 | 4.7 | 4.7 | 4.7 | 3.5 | 3.4 | 3.1 | 3.3 | 4.7 | 5.1 | 4.9 | 5.1 |
| Sales-SP | 354480 | 6 | 6.2 | 6.1 | 7 | 7.2 | 7.5 | 11.3 | 11.1 | 11.5 | 12.7 | 13.4 |
| Sales Oliveira-SP | 354490 | 37.4 | 37.7 | 39.5 | 34.6 | 36.2 | 36.6 | 40.3 | 39.9 | 38.8 | 37.9 | 28.4 |
| Salesópolis-SP | 354500 | 11.2 | 12.2 | 13.9 | 12.4 | 13.4 | 15 | 13.5 | 14 | 13.3 | 13.3 | 12.4 |
| Salmourão-SP | 354510 | 1.6 | 1.7 | 2 | 2.2 | 2.4 | 2.4 | 2.9 | 4.7 | 2.6 | 4.1 | 5.2 |
| Saltinho-SP | 354515 | 31.5 | 31.5 | 33.7 | 34.3 | 37.1 | 47.5 | 46.7 | 44.6 | 42.9 | 44.1 | 44.2 |
| Salto-SP | 354520 | 30.8 | 30.5 | 32.9 | 34.2 | 37.8 | 40.7 | 41.4 | 40.4 | 39.3 | 38.8 | 39.7 |
| Salto de Pirapora-SP | 354530 | 17.3 | 17.4 | 20.8 | 21.5 | 19.3 | 21.9 | 22.4 | 22.5 | 20.6 | 19.6 | 18.6 |
| Salto Grande-SP | 354540 | 5.1 | 5.8 | 5.4 | 5.9 | 8.1 | 7.4 | 8 | 7.7 | 8.4 | 7.6 | 7.7 |
| Sandovalina-SP | 354550 | 21.2 | 24.3 | 23.4 | 39.7 | 51.3 | 58.5 | 79 | 74 | 73.6 | 73.7 | 67.2 |
| Santa Adélia-SP | 354560 | 22.9 | 27.3 | 27 | 30.7 | 37 | 39 | 39.8 | 39.9 | 38 | 39.4 | 37.9 |
| Santa Albertina-SP | 354570 | 9.3 | 14.4 | 16.2 | 19.1 | 23.9 | 24.1 | 25.5 | 27.6 | 26.7 | 28.3 | 27.3 |
| Santa Bárbara d'Oeste-SP | 354580 | 36.6 | 38.1 | 40.8 | 42.3 | 42.8 | 44.9 | 45.3 | 43.4 | 42.1 | 42.3 | 41.6 |
| Santa Branca-SP | 354600 | 20.1 | 25 | 27.2 | 29.1 | 27 | 23.4 | 23.4 | 22.1 | 21 | 20.5 | 17.7 |
| Santa Clara d'Oeste-SP | 354610 | 3 | 3.9 | 5 | 5 | 4.6 | 5.1 | 5.5 | 5.7 | 6.5 | 6.2 | 6.4 |
| Santa Cruz da Conceição-SP | 354620 | 14.3 | 16.4 | 17.5 | 19.1 | 20.1 | 23.5 | 24.7 | 23.8 | 22.8 | 23.2 | 23.2 |
| Santa Cruz da Esperança-SP | 354625 | 5.8 | 7.4 | 8.4 | 8.8 | 8.8 | 9.3 | 9.5 | 9.9 | 9.2 | 8.5 | 8.7 |
| Santa Cruz das Palmeiras-SP | 354630 | 15.1 | 33.1 | 35.2 | 31.2 | 36.5 | 37.9 | 42.8 | 41.5 | 35.3 | 31.7 | 32.1 |
| Santa Cruz do Rio Pardo-SP | 354640 | 15.3 | 15.6 | 17.1 | 18.1 | 18.5 | 19.8 | 20.4 | 21.1 | 20.7 | 20.6 | 20.5 |
| Santa Ernestina-SP | 354650 | 34.5 | 38.1 | 35.7 | 38.3 | 41.2 | 49.4 | 52.1 | 45.2 | 46.5 | 42.6 | 42.5 |
| Santa Fé do Sul-SP | 354660 | 12.7 | 13.6 | 14.6 | 14 | 14.5 | 15.3 | 17.4 | 17.8 | 17.9 | 17.6 | 18.1 |
| Santa Gertrudes-SP | 354670 | 31 | 31.2 | 31.9 | 34.8 | 35.5 | 35.8 | 35.5 | 33 | 32.3 | 31.9 | 32.1 |
| Santa Isabel-SP | 354680 | 13.7 | 13.7 | 13.4 | 15.2 | 20.3 | 18.4 | 18.9 | 18 | 17.1 | 16.5 | 15.5 |
| Santa Lúcia-SP | 354690 | 42.8 | 45.2 | 48.1 | 50.5 | 51.7 | 49 | 44.4 | 45.2 | 43.3 | 43 | 42.4 |
| Santa Maria da Serra-SP | 354700 | 7.3 | 8.2 | 10.2 | 10.7 | 12.3 | 13.8 | 13 | 8.8 | 9.8 | 9.9 | 14.5 |
| Santa Mercedes-SP | 354710 | 3.7 | 4.7 | 4.8 | 4.8 | 7.4 | 7.5 | 7.2 | 7.8 | 7.5 | 7.9 | 8.5 |
| Santana da Ponte Pensa-SP | 354720 | 3.2 | 3.3 | 3.9 | 3.9 | 3.9 | 4.6 | 5 | 5.3 | 6.2 | 6.7 | 7 |
| Santana de Parnaíba-SP | 354730 | 41.4 | 42.2 | 45.3 | 43.5 | 44 | 45.4 | 46.7 | 44.8 | 42.3 | 41.2 | 40.5 |
| Santa Rita d'Oeste-SP | 354740 | 1.7 | 2.9 | 4.1 | 6 | 8.9 | 8.2 | 8.2 | 8.9 | 8.3 | 9.4 | 9 |
| Santa Rita do Passa Quatr-SP | 354750 | 36.8 | 39.8 | 40.2 | 41.2 | 40.6 | 39.6 | 35.8 | 34.2 | 33.4 | 32.9 | 33.1 |
| Santa Rosa de Viterbo-SP | 354760 | 28.6 | 35.5 | 35.7 | 36.7 | 37.9 | 38.8 | 40.3 | 35.6 | 33 | 33.8 | 33.2 |
| Santa Salete-SP | 354765 | 9 | 9.9 | 6.8 | 5 | 7.7 | 8.4 | 8.9 | 9.7 | 10 | 9.3 | 10.7 |
| Santo Anastácio-SP | 354770 | 18 | 18.7 | 18.7 | 19 | 19.3 | 19.5 | 19.6 | 19.5 | 19.6 | 19.4 | 19.3 |
| Santo André-SP | 354780 | 57.6 | 56.7 | 56.5 | 57.4 | 56.5 | 59 | 59.2 | 59.3 | 57.6 | 56.7 | 57.1 |
| Santo Antônio da Alegria-SP | 354790 | 4.2 | 4.6 | 4.6 | 4.9 | 4.8 | 4.8 | 4.9 | 4.8 | 4.4 | 4.5 | 4.8 |
| Santo Antônio de Posse-SP | 354800 | 10.9 | 12.3 | 15.6 | 20.2 | 21.2 | 23.7 | 25.6 | 22.4 | 22.2 | 20.8 | 21.3 |
| Santo Antônio do Aracangu-SP | 354805 | 1.7 | 2.4 | 4.2 | 4 | 7.6 | 13.4 | 13.2 | 11.6 | 10.5 | 9.8 | 14.6 |
| Santo Antônio do Jardim-SP | 354810 | 16.9 | 18.4 | 19.4 | 19.4 | 20.4 | 22.9 | 22.9 | 22.3 | 21.8 | 21.1 | 20.9 |
| Santo Antônio do Pinhal-SP | 354820 | 5.9 | 7.1 | 7.1 | 8 | 8.7 | 7.4 | 7.3 | 7.8 | 7.6 | 7.3 | 7.7 |
| Santo Expedito-SP | 354830 | 13.5 | 12.9 | 12.6 | 12.4 | 13.8 | 15.6 | 16.2 | 15.1 | 14.9 | 14.5 | 14.6 |
| Santópolis do Aguapeí-SP | 354840 | 2.6 | 3.6 | 3.7 | 4.3 | 5.4 | 7.4 | 7.6 | 7.9 | 8.4 | 8.5 | 8.1 |
| Santos-SP | 354850 | 64.2 | 63.1 | 63.6 | 63.4 | 63.8 | 64.2 | 65.5 | 63.9 | 61.5 | 60.6 | 59.8 |
| São Bento do Sapucaí-SP | 354860 | 4.9 | 5.6 | 6 | 6 | 7.6 | 7.9 | 7.3 | 6.9 | 7 | 6.6 | 6.7 |
| São Bernardo do Campo-SP | 354870 | 56.1 | 54.3 | 56 | 55.4 | 55.1 | 57.6 | 57.3 | 55.5 | 53 | 51.8 | 52 |
| São Caetano do Sul-SP | 354880 | 106 | 89 | 85.3 | 65.1 | 66.7 | 68.6 | 66.3 | 65.3 | 66.5 | 58.7 | 61.2 |
| São Carlos-SP | 354890 | 38.6 | 38.6 | 38.4 | 37.6 | 40.6 | 40.6 | 40.9 | 40.8 | 40.1 | 39.3 | 39.6 |
| São Francisco-SP | 354900 | 14.8 | 11.5 | 8.1 | 8.9 | 7.1 | 6 | 5.9 | 6.3 | 6.7 | 6.6 | 6.5 |
| São João da Boa Vista-SP | 354910 | 34 | 36 | 37.2 | 37.8 | 39 | 40.5 | 41.2 | 40.3 | 39.5 | 39 | 38 |
| São João das Duas Pontes-SP | 354920 | 3.7 | 4.4 | 5.4 | 5 | 5.7 | 7.1 | 8.3 | 8 | 8.2 | 7.6 | 8.2 |
| São João de Iracema-SP | 354925 | 8.4 | 8.6 | 9.8 | 10.1 | 10.2 | 11 | 12.1 | 11.1 | 11 | 10.8 | 11.5 |
| São João do Pau d'Alho-SP | 354930 | 2.1 | 2.5 | 3.1 | 6 | 15.9 | 21.5 | 22.3 | 21.3 | 23.4 | 21.2 | 19.7 |
| São Joaquim da Barra-SP | 354940 | 48.7 | 43.6 | 45.5 | 42.4 | 30.3 | 45.2 | 43.5 | 42.6 | 40.7 | 39.6 | 37.3 |
| São José da Bela Vista-SP | 354950 | 17.8 | 17.6 | 17.5 | 17 | 16.4 | 17.8 | 15.5 | 14.9 | 15.3 | 15.1 | 13.9 |
| São José do Barreiro-SP | 354960 | 6.5 | 8.3 | 7.3 | 8.1 | 9.4 | 7.9 | 7.4 | 8 | 6.9 | 4.5 | 4.2 |
| São José do Rio Pardo-SP | 354970 | 34.1 | 39.2 | 39.8 | 41 | 42.3 | 43.4 | 43.7 | 43.3 | 43.8 | 34.7 | 33.6 |
| São José do Rio Preto-SP | 354980 | 41.4 | 43 | 43.6 | 43.7 | 44.6 | 44.8 | 46 | 45.9 | 45.3 | 44.3 | 44 |
| São José dos Campos-SP | 354990 | 50.1 | 51.4 | 51.4 | 49 | 47.7 | 48 | 47.3 | 45.4 | 43.5 | 43.2 | 42.4 |
| São Lourenço da Serra-SP | 354995 | 24 | 19.1 | 19.4 | 19.1 | 15 | 14.5 | 13.9 | 13.2 | 11.3 | 10.9 | 10.8 |
| São Luís do Paraitinga-SP | 355000 | 8.2 | 10.2 | 11.2 | 10.7 | 12.3 | 15.4 | 15.7 | 14.2 | 13.8 | 13.1 | 12.2 |
| São Manuel-SP | 355010 | 31.9 | 30.6 | 31.8 | 29.8 | 29.5 | 29.8 | 28.7 | 27.3 | 26.3 | 25.8 | 26 |
| São Miguel Arcanjo-SP | 355020 | 4.9 | 4.8 | 6.7 | 7.1 | 7.8 | 7.9 | 7.5 | 6.2 | 6.5 | 6.6 | 6.7 |
| São Paulo-SP | 355030 | 54.6 | 52.9 | 53.2 | 54.3 | 54.8 | 55.5 | 55.3 | 53.3 | 49.8 | 47.8 | 47.2 |
| São Pedro-SP | 355040 | 22.8 | 23.4 | 23.6 | 23.7 | 24.3 | 25.3 | 26.1 | 24.9 | 23.5 | 22.9 | 22.5 |
| São Pedro do Turvo-SP | 355050 | 172.3 | 236.8 | 236.7 | 248.5 | 196.7 | 120.4 | 108.9 | 95 | 84.7 | 78.3 | 72.8 |
| São Roque-SP | 355060 | 31 | 30 | 30.4 | 30.9 | 31 | 32 | 33.7 | 33 | 34.3 | 27.3 | 26.5 |
| São Sebastião-SP | 355070 | 19.2 | 18.6 | 18.6 | 17.9 | 18.4 | 20.1 | 19.8 | 16.6 | 17.1 | 17.2 | 17.3 |
| São Sebastião da Grama-SP | 355080 | 10.5 | 12.1 | 13.2 | 13 | 13.5 | 13.5 | 14 | 13 | 13 | 12.9 | 13 |
| São Simão-SP | 355090 | 19.9 | 24.6 | 23.6 | 24.9 | 25.8 | 25.7 | 27 | 24.9 | 23.8 | 23.9 | 23 |
| São Vicente-SP | 355100 | 35.1 | 35.8 | 37.8 | 38.6 | 38.7 | 39.8 | 39.8 | 37.6 | 34.8 | 33.8 | 34.3 |
| Sarapuí-SP | 355110 | 4.3 | 5 | 5 | 5.3 | 5.3 | 6 | 6.2 | 6.5 | 6.4 | 6.4 | 6.4 |
| Sarutaiá-SP | 355120 | 3.7 | 4.8 | 5.4 | 7.4 | 8.2 | 6.4 | 5.2 | 4.6 | 3.1 | 2.7 | 3.7 |
| Sebastianópolis do Sul-SP | 355130 | 47.9 | 36.2 | 29.2 | 34.1 | 35 | 38.5 | 40.7 | 36.9 | 36.9 | 39 | 37.8 |
| Serra Azul-SP | 355140 | 12.3 | 14.6 | 14.6 | 14 | 14.2 | 14.5 | 14.1 | 15.2 | 13.9 | 13.5 | 13 |
| Serrana-SP | 355150 | 31.2 | 33 | 32.4 | 32.2 | 32.5 | 33.6 | 32.6 | 35.6 | 34 | 33.1 | 32.5 |
| Serra Negra-SP | 355160 | 17.9 | 18.4 | 18.9 | 19.4 | 19.7 | 20.1 | 20.4 | 20.4 | 20.1 | 19.4 | 18.9 |
| Sertãozinho-SP | 355170 | 51.6 | 49.5 | 50 | 51.3 | 49.9 | 51 | 51 | 47.2 | 45.5 | 42.6 | 41.9 |
| Sete Barras-SP | 355180 | 5.6 | 6 | 6.1 | 6.1 | 6.6 | 6.7 | 6.7 | 6.3 | 6 | 5.8 | 5.8 |
| Severínia-SP | 355190 | 18.9 | 22.5 | 24.8 | 26.4 | 27.3 | 26.6 | 28 | 27.8 | 25.8 | 25.4 | 25.2 |
| Silveiras-SP | 355200 | 5.5 | 5.4 | 6 | 6.3 | 6.5 | 6.7 | 6.7 | 6.8 | 5.8 | 5.4 | 5.3 |
| Socorro-SP | 355210 | 12.3 | 12.8 | 13.3 | 13 | 13.1 | 13.3 | 11.8 | 11.7 | 11.2 | 10.7 | 10.4 |
| Sorocaba-SP | 355220 | 42.9 | 40.5 | 43 | 44.2 | 46.1 | 49 | 49 | 46.6 | 44.8 | 42.6 | 42.4 |
| Sud Mennucci-SP | 355230 | 4.1 | 4.5 | 5.3 | 6.9 | 30.4 | 26.8 | 27.1 | 27.9 | 27.2 | 28.4 | 27.3 |
| Sumaré-SP | 355240 | 31.9 | 32.4 | 35.9 | 37 | 39.4 | 39.3 | 41.9 | 38.4 | 36 | 35.4 | 35.2 |
| Suzano-SP | 355250 | 29.9 | 26.8 | 28.9 | 29 | 28.8 | 30.1 | 30.2 | 28.7 | 27.2 | 27.2 | 26.8 |
| Suzanápolis-SP | 355255 | 10.2 | 12.4 | 22.6 | 25.9 | 32.8 | 45 | 33.8 | 31.9 | 29.8 | 30.7 | 30.2 |
| Tabapuã-SP | 355260 | 24.5 | 23.8 | 24.6 | 30.7 | 30.4 | 31 | 31.1 | 28.4 | 26.2 | 25.6 | 25.4 |
| Tabatinga-SP | 355270 | 18.2 | 17.8 | 17.4 | 27.4 | 27.9 | 28.9 | 20.2 | 21.9 | 19.2 | 18 | 17.1 |
| Taboão da Serra-SP | 355280 | 31.9 | 31.5 | 31.9 | 33.6 | 34.1 | 35.5 | 35.9 | 33.6 | 31.1 | 30.1 | 29.6 |
| Taciba-SP | 355290 | 11.5 | 12.2 | 12 | 12.4 | 12.5 | 12.9 | 13.5 | 13.5 | 13.3 | 12.7 | 12.8 |
| Taguaí-SP | 355300 | 1.8 | 1.6 | 1.6 | 1.8 | 1.8 | 1.6 | 1.5 | 1.7 | 1.5 | 1.4 | 1.5 |
| Taiaçu-SP | 355310 | 19.8 | 19.3 | 18.2 | 17.7 | 17.6 | 17.6 | 16.8 | 16.7 | 16.1 | 16 | 15 |
| Taiúva-SP | 355320 | 30.3 | 32.8 | 33.1 | 30.1 | 28.8 | 27.1 | 25.6 | 23.9 | 23.5 | 23.4 | 23.4 |
| Tambaú-SP | 355330 | 20.9 | 21.7 | 22.7 | 22.4 | 25.3 | 25.5 | 25.4 | 24.8 | 23.1 | 23.3 | 22.6 |
| Tanabi-SP | 355340 | 21.3 | 23.1 | 24.8 | 25.4 | 26.7 | 26.8 | 28.8 | 27.7 | 27 | 27.4 | 27.4 |
| Tapiraí-SP | 355350 | 2.5 | 2.3 | 4.8 | 4.6 | 5 | 5.8 | 6.4 | 6.9 | 7.2 | 7.5 | 7.1 |
| Tapiratiba-SP | 355360 | 15.1 | 17.4 | 16.5 | 17.5 | 18.3 | 18.4 | 16.5 | 16 | 16.1 | 20.5 | 19.8 |
| Taquaral-SP | 355365 | 17.8 | 18.9 | 19.1 | 21.7 | 18.3 | 18.5 | 17 | 16.9 | 16.4 | 16.4 | 16.2 |
| Taquaritinga-SP | 355370 | 24.1 | 25.5 | 26.9 | 26.6 | 27.3 | 27.8 | 28.5 | 28.5 | 28.2 | 28 | 27.4 |
| Taquarituba-SP | 355380 | 3.6 | 4.1 | 4.3 | 4.2 | 4.3 | 4.9 | 5 | 4.8 | 4.8 | 4.9 | 5 |
| Taquarivaí-SP | 355385 | 1.9 | 2 | 2 | 5.5 | 7.2 | 10.4 | 10.6 | 9.5 | 9.3 | 10.3 | 11.6 |
| Tarabai-SP | 355390 | 11.2 | 13.3 | 11.9 | 13.1 | 14.9 | 11.1 | 11.5 | 10.8 | 10.3 | 10.3 | 10.1 |
| Tarumã-SP | 355395 | 4.1 | 4.5 | 6 | 6.9 | 6.9 | 7.9 | 8.4 | 8.3 | 11.6 | 11.8 | 13.1 |
| Tatuí-SP | 355400 | 27 | 26.9 | 29.5 | 29.5 | 29.2 | 29.5 | 28.1 | 27.6 | 26.3 | 25.5 | 25 |
| Taubaté-SP | 355410 | 36.3 | 36.5 | 40.1 | 41.8 | 39.8 | 41.9 | 40.7 | 37 | 34.5 | 34.3 | 33.4 |
| Tejupá-SP | 355420 | 1.1 | 0.8 | 1 | 0.9 | 1.1 | 1.4 | 1.2 | 1.2 | 1 | 0.9 | 1 |
| Teodoro Sampaio-SP | 355430 | 9.2 | 11.2 | 14.4 | 18.4 | 20.7 | 23.9 | 23.5 | 20.1 | 18 | 17.8 | 19.4 |
| Terra Roxa-SP | 355440 | 16.4 | 13.3 | 21.6 | 13.3 | 20.6 | 21 | 21.3 | 20.8 | 20.3 | 20.9 | 20.5 |
| Tietê-SP | 355450 | 32.9 | 32.2 | 33.2 | 30.2 | 29.9 | 32.7 | 33.5 | 33.8 | 33.4 | 34 | 33.8 |
| Timburi-SP | 355460 | 2.1 | 2.3 | 2.4 | 2.4 | 2.3 | 2.3 | 2.5 | 2.3 | 2.6 | 2.7 | 3.2 |
| Torre de Pedra-SP | 355465 | 3.1 | 3.1 | 3.3 | 3 | 2.9 | 2.5 | 3.4 | 3.8 | 3.9 | 4.2 | 3.8 |
| Torrinha-SP | 355470 | 4.9 | 5 | 5.2 | 5.5 | 5.8 | 6.4 | 6.4 | 6.3 | 6.4 | 6.9 | 7.7 |
| Trabiju-SP | 355475 | 14.7 | 14.6 | 16.9 | 19.3 | 19.8 | 21.8 | 19.4 | 19.1 | 19.2 | 18.9 | 19.7 |
| Tremembé-SP | 355480 | 21.2 | 21.7 | 22.7 | 24.3 | 24.6 | 26.4 | 25.7 | 24.5 | 23 | 22.8 | 22.3 |
| Três Fronteiras-SP | 355490 | 4.6 | 5.6 | 6.4 | 6 | 6.7 | 7.5 | 10.1 | 10.7 | 11.4 | 11.7 | 11.8 |
| Tuiuti-SP | 355495 | 11.4 | 13.8 | 15.8 | 17 | 16.3 | 16 | 15.9 | 15 | 14.5 | 14.1 | 12.8 |
| Tupã-SP | 355500 | 17.8 | 19 | 20.1 | 20.3 | 20.5 | 18.9 | 19.5 | 19.7 | 19.5 | 19.2 | 18.9 |
| Tupi Paulista-SP | 355510 | 13.4 | 14 | 14.6 | 15.3 | 16.3 | 16.9 | 17.4 | 16 | 15.9 | 16.5 | 16.8 |
| Turiúba-SP | 355520 | 24.1 | 24.2 | 25.8 | 26.4 | 27 | 29.9 | 27.7 | 27.6 | 28.2 | 27.1 | 21.3 |
| Turmalina-SP | 355530 | 5.1 | 6.1 | 9 | 10.5 | 12.7 | 13.2 | 12.5 | 13 | 11.6 | 12.8 | 11.1 |
| Ubarana-SP | 355535 | 8.8 | 10 | 9.6 | 10.2 | 12.2 | 13.9 | 14.2 | 14.7 | 15.1 | 15.7 | 11.8 |
| Ubatuba-SP | 355540 | 14.3 | 14.4 | 14 | 12.9 | 11.5 | 11.4 | 11.8 | 12.2 | 12.4 | 12 | 11.9 |
| Ubirajara-SP | 355550 | 3.3 | 3.5 | 3.4 | 3.4 | 3.7 | 3.7 | 3.9 | 3.9 | 3.8 | 3.8 | 4.1 |
| Uchoa-SP | 355560 | 29.9 | 24.8 | 26 | 25.5 | 27.4 | 28 | 30.1 | 25.2 | 26.9 | 29.9 | 26.2 |
| União Paulista-SP | 355570 | 6.8 | 7.8 | 12.1 | 12 | 20 | 22.8 | 29.3 | 16.9 | 9.6 | 11 | 11.2 |
| Urânia-SP | 355580 | 6.9 | 7.4 | 10.1 | 12.3 | 12.2 | 11.7 | 13.1 | 13.3 | 13.3 | 13.6 | 13 |
| Uru-SP | 355590 | 10.8 | 11.5 | 12.6 | 12 | 14.2 | 15.6 | 16.7 | 15.8 | 15.6 | 17.3 | 16.8 |
| Urupês-SP | 355600 | 17.8 | 18.1 | 21 | 22.5 | 24 | 25 | 26.6 | 25.9 | 26.2 | 26.2 | 26.8 |
| Valentim Gentil-SP | 355610 | 7.8 | 9.9 | 12.3 | 15.2 | 14.8 | 15.4 | 16.4 | 14.7 | 15.1 | 15.5 | 15.8 |
| Valinhos-SP | 355620 | 50 | 50.6 | 53.7 | 53.9 | 55.5 | 57.4 | 58 | 54.9 | 53.6 | 52.4 | 51.4 |
| Valparaíso-SP | 355630 | 7.4 | 7.8 | 8.2 | 10.2 | 17 | 18.6 | 19.9 | 18.3 | 17.4 | 19.9 | 26.4 |
| Vargem-SP | 355635 | 15.6 | 18 | 22 | 24.6 | 25.6 | 23.8 | 22.3 | 21.8 | 21.5 | 20.7 | 20.9 |
| Vargem Grande do Sul-SP | 355640 | 20.8 | 24.5 | 27.5 | 28.1 | 31.4 | 30 | 32.8 | 31.9 | 27.7 | 26.4 | 26.2 |
| Vargem Grande Paulista-SP | 355645 | 24.2 | 23.2 | 26.2 | 27.4 | 27.2 | 27.3 | 27.4 | 27 | 25.3 | 24.4 | 22 |
| Várzea Paulista-SP | 355650 | 40.5 | 43.4 | 48.8 | 45.5 | 47.8 | 50.6 | 50.1 | 46.9 | 45.4 | 44.8 | 44.3 |
| Vera Cruz-SP | 355660 | 9.5 | 10.3 | 10.7 | 11.3 | 12.5 | 13.7 | 14.4 | 13.6 | 14.4 | 14.5 | 15.1 |
| Vinhedo-SP | 355670 | 60.1 | 60.6 | 61.8 | 56.5 | 56.8 | 58 | 55.2 | 53 | 49.8 | 48.1 | 47.5 |
| Viradouro-SP | 355680 | 35.9 | 38.9 | 42.6 | 33 | 35.4 | 34.1 | 35 | 36.7 | 35.6 | 35.5 | 35.1 |
| Vista Alegre do Alto-SP | 355690 | 14.2 | 15.2 | 11.1 | 13.4 | 15.4 | 14.6 | 14 | 14.2 | 13.8 | 13.4 | 12.8 |
| Vitória Brasil-SP | 355695 | 2.4 | 3.3 | 3.8 | 4.4 | 6.9 | 6.8 | 7 | 7.7 | 6.6 | 6.7 | 6.8 |
| Votorantim-SP | 355700 | 36.6 | 35.5 | 38.3 | 36.4 | 39.1 | 40.9 | 41.4 | 39.9 | 38.1 | 34.8 | 35.5 |
| Votuporanga-SP | 355710 | 28.9 | 31.5 | 32.9 | 32.4 | 33.2 | 35 | 36 | 31.9 | 31.1 | 31.3 | 31.9 |
| Zacarias-SP | 355715 | 8.9 | 11.5 | 12.4 | 12 | 14.9 | 15.3 | 17 | 15.7 | 15.4 | 15.7 | 11.9 |
| Chavantes-SP | 355720 | 10.8 | 11.1 | 13.6 | 16.3 | 18.1 | 19.2 | 18.7 | 18.4 | 18.7 | 18.1 | 17.5 |
| Estiva Gerbi-SP | 355730 | 27.6 | 28.4 | 28.8 | 31.5 | 30 | 32.1 | 32.2 | 31.3 | 28.8 | 29.4 | 28.8 |

Source: PROADESS (https://www.proadess.icict.fiocruz.br) and IBGE (<https://www.ibge.gov.br/explica/codigos-dos-municipios.php>)
