# Supplementary material for: Development and validation of an epidemiological risk score for neonatal death in a middle-income country
Source: Front Public Health. 2025 Nov 19;13:1675040. doi: 10.3389/fpubh.2025.1675040 (PMC12672502; doi:10.3389/fpubh.2025.1675040)
Supplement: Supplementary file 14 [file Table_14.docx]

**Supplementary Material 14. Absolute and relative frequency of the neonatal risk score among live births with complete data for the risk score model – internal validation. State of São Paulo, 2009–2018.**

| **Score** | **N** | **%** |
| --- | --- | --- |
| 0 | 28,231 | 0.5 |
| 1 | 241,758 | 4.3 |
| 2 | 850,030 | 15.0 |
| 3 | 1,393,910 | 24.6 |
| 4 | 1,141,973 | 20.2 |
| 5 | 597,934 | 10.6 |
| 6 | 434,642 | 7.7 |
| 7 | 339,247 | 6.0 |
| 8 | 154,489 | 2.7 |
| 9 | 85,712 | 1.5 |
| 10 | 100,703 | 1.8 |
| 11 | 86,149 | 1.5 |
| 12 | 55,623 | 1.0 |
| 13 | 56,954 | 1.0 |
| 14 | 58,302 | 1.0 |
| 15 | 29,299 | 0.5 |
| 16 | 6,251 | 0.1 |
| 17 | 2,019 | 0.0 |
| 18 | 1,688 | 0.0 |
| 19 | 1,045 | 0.0 |
| 20 | 666 | 0.0 |
| 21 | 646 | 0.0 |
| 22 | 328 | 0.0 |
| 23 | 177 | 0.0 |
| 24 | 145 | 0.0 |
| 25 | 79 | 0.0 |
| 26 | 11 | 0.0 |
| Total | 5,668,011 | 100.0 |
