# Supplementary material for: Development and validation of an epidemiological risk score for neonatal death in a middle-income country
Source: Front Public Health. 2025 Nov 19;13:1675040. doi: 10.3389/fpubh.2025.1675040 (PMC12672502; doi:10.3389/fpubh.2025.1675040)
Supplement: Supplementary file 15 [file Table_15.docx]

**Supplementary Material 15. Absolute and relative frequency of neonatal deaths, and odds ratios by neonatal risk score – internal validation. State of São Paulo, 2009–2018.**

| **Score** | **N deaths** | **% deaths** | **Odds ratio** |
| --- | --- | --- | --- |
| 0 | 41 | 0.2 | 1.0 |
| 1 | 296 | 0.1 | 0.8 |
| 2 | 930 | 0.1 | 0.8 |
| 3 | 1,545 | 0.1 | 0.8 |
| 4 | 1,317 | 0.1 | 0.8 |
| 5 | 938 | 0.2 | 1.1 |
| 6 | 1,026 | 0.2 | 1.6 |
| 7 | 1,107 | 0.3 | 2.3 |
| 8 | 832 | 0.5 | 3.7 |
| 9 | 1,164 | 1.4 | 9.5 |
| 10 | 2,256 | 2.2 | 15.8 |
| 11 | 2,774 | 3.2 | 22.9 |
| 12 | 3,480 | 6.3 | 45.9 |
| 13 | 6,597 | 11.6 | 90.1 |
| 14 | 7,901 | 13.6 | 107.8 |
| 15 | 4,308 | 14.7 | 118.5 |
| 16 | 1,199 | 19.2 | 163.2 |
| 17 | 781 | 38.7 | 433.8 |
| 18 | 695 | 41.2 | 481.2 |
| 19 | 495 | 47.4 | 618.8 |
| 20 | 361 | 54.2 | 813.8 |
| 21 | 412 | 63.8 | 1,210.6 |
| 22 | 211 | 64.3 | 1,240.0 |
| 23 | 141 | 79.7 | 2,693.0 |
| 24 | 121 | 83.5 | 3,466.5 |
| 25 | 66 | 83.5 | 3,490.7 |
| 26 | 11 | 100.0 | - |
| **Total** | **41,005** | **-** | **-** |
